# Therapeutic efficacy of TMTP1-modified EVs in overcoming bone metastasis and immune resistance in PIK3CA mutant NSCLC

**DOI:** 10.1038/s41419-025-07685-y

**Published:** 2025-05-06

**Authors:** Liwen Liu, Tanghesi Wuyun, Xin Sun, Yu Zhang, Geqi Cha, Ling Zhao

**Affiliations:** 1 Department of Radiology, Harbin Medical University Cancer Hospital, Harbin, China; 2 The Second Department of Respiratory, Harbin Medical University Cancer Hospital, Harbin, China; 3 Department of Radiation Oncology, Harbin Medical University Cancer Hospital, Harbin, China

**Keywords:** Cancer, Cell biology

## Abstract

Non-small cell lung cancer (NSCLC) with PIK3CA mutations demonstrates significant challenges in treatment due to enhanced bone metastasis and immune checkpoint resistance. This study investigates the efficacy of tumor-targeting peptide 1-modified cancer stem cell-derived extracellular vesicles (TMTP1-TSRP-EVs) in reshaping the tumor microenvironment and reversing immune checkpoint resistance in NSCLC. By integrating TMTP1-TSRP into EVs, we aim to specifically deliver therapeutic agents to NSCLC cells, focusing on inhibiting the PI3K/Akt/mTOR pathway, a crucial driver of oncogenic activity and immune evasion in PIK3CA-mutated cells. Our comprehensive in vitro and in vivo analyses show that TMTP1-TSRP-EVs significantly inhibit tumor growth, reduce PD-L1 expression, and enhance CD8^+^ T cell infiltration, effectively reversing the immune-suppressive microenvironment. Moreover, the in vivo models confirm that our approach not only suppresses bone metastases but also overcomes primary resistance to immune checkpoint inhibitors by modulating the expression of key immunological markers. These findings suggest that targeted delivery of TMTP1-TSRP-EVs could provide a novel therapeutic strategy for treating PIK3CA-mutant NSCLC, offering significant improvements over traditional therapies by directly targeting the molecular pathogenesis of tumor resistance and metastasis.

**Figure:**
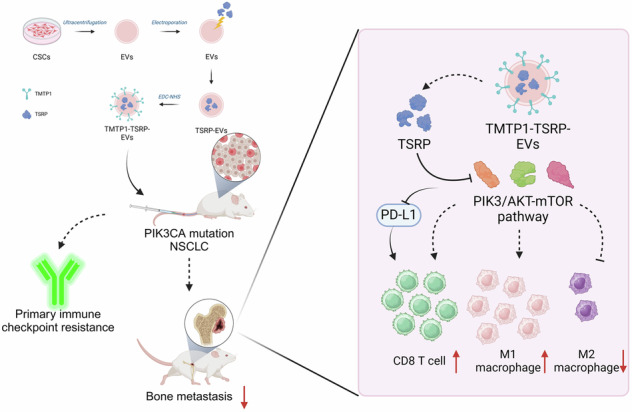
Molecular Mechanisms Reshaping the TME to Halt PI3K-Mutant Bone Metastasis of NSCLC and Overcoming Primary ICI Resistance. (Created by BioRender).

Molecular Mechanisms Reshaping the TME to Halt PI3K-Mutant Bone Metastasis of NSCLC and Overcoming Primary ICI Resistance. (Created by BioRender).

## Introduction

Non-small cell lung cancer (NSCLC) is a significant contributor to cancer-related mortality worldwide, with high incidence and mortality rates [1–3]. Recent studies have highlighted the crucial role of the tumor microenvironment (TME) in the initiation, progression, and development of resistance to treatment in tumors [4, 5]. The TME comprises various components such as tumor cells, immune cells, stromal cells, and blood vessels. These components interact through intricate signaling networks to regulate tumor growth and dissemination [6, 7]. Particularly, alterations in the immune microenvironment significantly impact immune evasion by tumors and resistance to treatment [8–10]. While traditional cancer therapies mainly target tumor cells, emerging research indicates that modulating the TME can significantly enhance treatment efficacy [11]. Consequently, targeting the TME has become a vital direction in cancer research [12–14].

The abnormal activation of the PI3K/Akt/mTOR signaling pathway has been extensively studied in various cancers, particularly in NSCLC [15–17]. Mutations in the PIK3CA gene represent a common mechanism of activation in this pathway, closely linked to tumor cell proliferation, survival, invasion, and metastasis [18–20]. Studies have shown that PIK3CA mutations not only promote tumor growth and metastasis but also correlate closely with immune evasion and resistance to immune checkpoint inhibitors (ICIs) [21]. PIK3CA mutations impact the tumor immune microenvironment through various mechanisms, including facilitating the infiltration of immune-suppressive cells and impairing the function of effector T cells [22]. These findings suggest that targeted therapeutic strategies against PIK3CA mutations hold promise in overcoming the limitations of current treatment methods and enhancing treatment efficacy.

Cancer stem cells (CSCs) play a crucial role in tumor initiation, progression, and treatment resistance [23]. CSCs possess the ability to self-renew and differentiate, forming new tumor cells to sustain tumor growth and metastasis [24, 25]. Recent research has unveiled that CSCs regulate the TME by secreting extracellular vesicles (EVs) [26–28]. EVs, membrane-bound vesicles with diameters ranging from 30 to 150 nm, contain biological molecules such as proteins, RNA, and miRNA, enabling intercellular information transfer [29–31]. Utilizing CSC-derived EVs as drug carriers enhances drug targeting and therapeutic efficacy, offering novel avenues and approaches for anti-cancer therapy [32–34].

The transformable specific-responsive peptide (TSRP) is a novel peptide capable of specifically recognizing and targeting tumor cells, inhibiting tumor growth and metastasis by regulating key signaling pathways [35]. TSRP can suppress tumor cell proliferation and migration by downregulating the activity of the PI3K/Akt/mTOR signaling pathway. Furthermore, TSRP enhances tumor immune surveillance by increasing the infiltration of CD8^+^ T cells and inhibiting the expression of PD-L1, thereby reversing immune checkpoint resistance [35]. These findings provide a strong theoretical basis for the application of TSRP in NSCLC.

This study aims to investigate the mechanism of action of Tumor-Targeting Peptide 1-modified cancer stem cell-derived extracellular vesicles encapsulating transformable specific-responsive peptide (TMTP1-TSRP-EVs) in reshaping the TME, preventing bone metastasis of PI3K-mutant NSCLC, and reversing primary immune checkpoint resistance. Initially, we utilized bioinformatics analysis and CRISPR/Cas9 technology to establish PIK3CA mutant and wild-type NSCLC cell lines, identifying the key pathways and genes targeted by TSRP therapy. Subsequently, we isolated CSCs and their secreted EVs through sphere formation assays, followed by the construction of TMTP1-TSRP-EVs using chemical conjugation and electroporation techniques. Finally, both in vitro and in vivo experiments were conducted to validate the reshaping effect of the TME and the impact on bone metastasis of PI3K-mutant NSCLC and immune checkpoint resistance. This study not only aims to elucidate the molecular mechanisms underlying the interaction between PI3K mutations and the immune microenvironment but also provides novel insights and approaches for the targeted therapy of NSCLC, holding significant scientific and clinical implications. By comprehensively understanding the mechanism of action of TSRP and its clinical potential, we aspire to offer more effective treatment strategies for NSCLC patients, ultimately improving their prognosis and survival rates.

## Materials and methods

### Acquisition and analysis of NSCLC gene expression and mutation data

The single-nucleotide polymorphism (SNP) mutation data of TCGA-Lung Adenocarcinoma (LUAD) and TCGA-Lung Squamous Cell Carcinoma (LUSC) datasets were retrieved from the Cancer Genome Atlas (TCGA) database (https://portal.gdc.cancer.gov/). Specifically, the “Masked Somatic Mutation” data was selected for download, encompassing a total of 1100 cases of NSCLC patients (including TCGA-LUAD and TCGA-LUSC), providing insights into the somatic mutation landscape. Additionally, RNA-seq data (NSCLC-Counts data) from the TCGA-NSCLC dataset, comprising all available NSCLC samples (*N* = 1016) and normal tissues (*N* = 108), were obtained. Clinical information essential for the study, such as age, gender, TNM staging, histological grading, and overall survival (OS) status, was also acquired from TCGA. In addition, we obtained somatic gene mutation information for 100 samples of LUSC-KR from the International Cancer Genome Consortium (ICGC) database (https://dcc.icgc.org/) up to November 27, 2019. Only patients with complete clinical data were included, while those with missing information on TNM staging, gender, age, and survival status were excluded.

All bioinformatics statistical analyses were carried out using the R software version 4.2.1 and the corresponding software packages. For the analysis and visualization of TCGA mutation annotation format (MAF) files, the “maftools” package in R was utilized. Similarly, the analysis and visualization of mutation data from the IICGC were conducted using the “GenVisR” package in R. Subsequently, the Perl programming language was employed to extract highly mutated genes from TCGA and ICGC databases, and the intersection was obtained using the “venn” tool to identify genes with high mutation frequencies. Following this, based on the mutation severity of the mutated genes, the samples were categorized into wild-type and mutant groups. The relationship between these intersected genes and tumor mutation burden (TMB) was then assessed and visualized using the “ggpubr” package in R.

### TMB calculation

TMB refers to the total number of somatic mutations detected per million bases (mutations per Mb). In this study, we utilized whole-exome sequencing (WES) data of NSCLC from the TCGA database to calculate TMB scores. The Perl language was employed to compute the mutation frequency for each sample, defined as the number of mutations in each sample divided by the exome length (38 million).

### Analysis of the tumor immune microenvironment in NSCLC samples

Using the TIMER database, we inferred the abundance of infiltrating CD4^+^ T cells, CD8^+^ T cells, B cells, macrophages, neutrophils, and dendritic cells (DC) in NSCLC samples.

### Cell culture

The human NSCLC cell lines A549 (CCL-185) and H1703 (CRL-5889) were both obtained from ATCC (USA), while human CD8^+^ T cells (1506) were sourced from LDEBIO (China). A549, H1703, and CD8^+^ T cells were cultured in RPMI-1640 medium (A1049101, Gibco, USA) containing 10% FBS (12484028, Gibco, USA) and 1% penicillin/streptomycin (15140148, Gibco, USA). Before experimentation, CD8^+^ T cells were stimulated for 24 h with 2 µL of CD3/CD28 activator (11161D, Gibco, USA). When investigating the impact of primary immune checkpoint resistance, Nivolumab (200 μg/mL; T9907, TargetMol, USA) was introduced into the cell culture medium. For functional validation of TSRP, 2 mg/mL of TSRP (LSPPRYPCKLVFFPLGVRGKKWWKK-Dip-K-NH2) purchased from Hangzhou Dangang Biochemical Technology Co., Ltd. (China) was added to the cell culture medium for subsequent experiments. In mechanistic studies, 4 μg/mL of SC79 (HY-18749) obtained from MedChemExpress (USA) was added to the cell culture medium [36].

293 T cell line was obtained from ATCC (CRL-3216) and cultured in DMEM medium (11965092, Gibco, USA) containing 10% FBS, 10 μg/mL streptomycin, and 100 U/mL penicillin. The cells were maintained in a humidified cell culture incubator (Heracell™ Vios 160i CR CO_2_ incubator, 51033770, Thermo Scientific™, Germany) at 37 °C with 5% CO_2_. Passaging was performed when the cell growth reached 80%–90% confluency [37].

### Cell co-culture

NSCLC cells A549/H1703 were co-cultured in vitro with CD8^+^ T cells at a ratio of 1:5. The co-culture was continued for 48 h, followed by the collection of supernatant and NSCLC cells. Flow cytometry cell sorting of NSCLC cells was performed using the EpCAM antibody for further experiments [38–41].

The cell co-culture groups were as follows: (1) DMSO + PBS group: CD8^+^ T cells + A549/H1703 cells co-cultured with DMSO + PBS; (2) SC79 + PBS group: CD8^+^ T cells + A549/H1703 cells co-cultured with 4 μg/mL SC79 + PBS; (3) SC79 + TSRP group: CD8^+^ T cells + A549/H1703 cells co-cultured with 4 μg/mL SC79 + 2 mg/mL TSRP; (4) EVs group: CD8^+^ T cells + A549/H1703 cells co-cultured with CSCs-EVs; (5) TMTP1-EVs group: CD8^+^ T cells + A549/H1703 cells co-cultured with TMTP1-EVs; (6) TMTP1-EVs group: CD8^+^ T cells + A549/H1703 cells co-cultured with TMTP1-EVs; (7) TSRP-EVs group: CD8^+^ T cells + A549/H1703 cells co-cultured with TSRP-EVs; (8) TMTP1-TSRP-EVs group: CD8^+^ T cells + A549/H1703 cells co-cultured with TMTP1-TSRP-EVs. Prior to the co-culture of CD8^+^ T cells and A549/H1703 cells, 20 μg/mL of EVs from each group were added to the A549/H1703 cell culture medium for 24 h of co-incubation. DMSO, SC79, PBS, and TSRP solutions were added to the culture medium during the co-culture of CD8^+^ T cells and A549/H1703 cells.

### Generation of cells overexpressing wild-type PIK3CA and mutant PIK3CA overexpression cells

Cells overexpressing PIK3CA were generated using lentiviral transfection technology. The lentiviral vectors used were constructed by Genechem (Shanghai, China). The lentivirus overexpressing PIK3CA was packaged in the pLenti-RFP vector. These vectors were transduced into cells via lentivirus transduction and selected with puromycin (400051, Sigma–Aldrich, USA) to generate stable cells overexpressing PIK3CA. Using the CRISPR/Cas9 editing system, we generated the PIK3CA-E545K (c.1633 G > A) mutation. The mutation vector used was pCIG PIK3CA-E545K (73055, Addgene, USA), while the wild-type vector was pCIG PIK3CA Wildtype (73056, Addgene, USA). Targeted mutagenesis of p.E545K was performed in A549 or H1703 cells using the QuickChange II site-directed mutagenesis kit (200523, Agilent, USA) to produce the PIK3CA p.E545K variant and the PIK3CA wild-type (Fig. S1). Surviving cells were obtained through restrictive dilution cloning, and PIK3CA-E545K heterozygous and homozygous mutant cells were identified via DNA sequencing. Specifically, we obtained A549 wild-type and homozygous mutant cells (A549 WT/^−^; A549^−^/MUT) and H1703 wild-type and heterozygous mutant cells (H1703 WT/WT; H1703 WT/MUT).

### Fluorescence in situ hybridization (FISH)

FISH analysis was performed on formalin-fixed, paraffin-embedded NSCLC tissues using the Abbott-Vysis HER2/CEP17 dual-color probe (Abbott, USA, 05N56-020). Initially, tissue sections embedded in paraffin and deparaffinized with xylene were rehydrated in different ethanol gradients (100%, 85%, and 70%). Subsequently, the sections were treated with proteinase K solution (200 μg/mL; 25530015, Invitrogen, USA) and pepsin (0.005% in 0.01 M HCl solution; P7012, Sigma–Aldrich, USA) for further processing.

The slides were dehydrated in different ethanol gradients (70%, 85%, and 100%), and the probe mixture was added to the slides. Subsequently, coverslips were immediately placed on top, and the edges were sealed with rubber cement. The slides were denatured at 85 °C for 5 min and then incubated overnight at 37 °C. Following hybridization, FISH signals from 20 to 30 cells were counted. The standard definition for PIK3CA amplification was set as FISH signal ≥2.2 compared to the control probe. Fluorescence images were captured using an Olympus BX43 microscope (Olympus, Tokyo, Japan) under FITC and Texas Red wavelengths, and image processing was carried out using Gene Data Manager 7.2.7.33397.

### Characterization of TSRP

TSRP peptide was subjected to characterization experiments by adding 15 μg/mL of MMP-2 (HY-P73296, MedChemExpress, USA) into the culture medium.

For the HPLC analysis, a 4.6 × 250 nm Sinochrom ODS-BP column (5 μm) was utilized. The mobile phase consisted of 0.1% trifluoroacetic acid in 100% acetonitrile as solvent A and 0.1% trifluoroacetic acid in 100% water as solvent B. The retention time (RT) was observed at a wavelength of 220 nm with a flow rate of 1.0 mL/min.

Turbidity testing involved measuring the absorbance of different solution groups at 500 nm wavelength over a duration of 5 h, with readings taken every 15 s. Ultimately, data was collected using GraphPad software.

### CCK-8 experiment for cell viability assessment

CD8^+^ T cells were digested, resuspended, and adjusted to a concentration of 1 × 10^5^ cells/mL. Subsequently, 100 μL of cell suspension was seeded into a 96-well plate for standard cultivation. After cell adhesion, drugs were added for treatment, followed by overnight incubation. At 0, 12, 24, and 48 h post-incubation, cell viability was evaluated according to the instructions of the CCK-8 assay kit (C0041, Beyotime, Shanghai). During each assessment, 10 μL of CCK-8 detection solution was added, and the plate was then incubated in a cell culture incubator for 4 h. The absorbance at 450 nm was measured using an enzyme-linked immunosorbent assay (ELISA) reader to calculate cell viability, utilizing the formula: Cell Viability = (ΔA_sample - ΔA_blank) / (ΔA_control - ΔA_blank), where ΔA_sample represents the absorbance difference of the sample, ΔA_blank is the absorbance difference of the blank, and ΔA_control is the absorbance difference of the control group.

### Transwell experiment for assessing cell migration ability

NSCLC cells transfected for 48 h were collected and suspended in a serum-free medium at a concentration of 10^5^ cells per well. Subsequently, 200 μL of cell suspension (2 × 10^4^ cells/well) was seeded in the upper chamber of Transwell plates, while 800 μL of medium containing 20% FBS was added to the lower chamber. Following a 24-h incubation at 37 °C, the Transwell plates were removed, washed twice with PBS, fixed with formaldehyde for 10 min, and then rinsed three times with distilled water. The cells were stained with 0.1% crystal violet, incubated at room temperature for 30 min, and washed twice with PBS, and the migrated cells were photographed using an inverted light microscope (CKX53, Olympus, Japan). Cell counting and analysis of cancer cell migration ability were performed using ImageJ software.

### TUNEL staining

NSCLC cells from each group were fixed with 4% paraformaldehyde at room temperature for 15 min and then permeabilized with 0.25% Triton X-100 for 20 min. Samples were blocked with 5% bovine serum albumin (BSA, 36101ES25, Yeasen Biotechnology (Shanghai) Co., Ltd., China) and subsequently stained with TUNEL (C1086, Beyotime Biotechnology Co., Ltd, Shanghai, China) reagent. DAPI staining solution (C1002, Beyotime Biotechnology Co., Ltd, Shanghai, China) was applied to counterstain in the dark. Apoptotic cell images were captured under a confocal microscope (LSM 880, Carl Zeiss AG, Germany). TUNEL-positive cells (green fluorescence) indicated apoptotic cells, while DAPI-labeled cell nuclei emitting blue fluorescence represented the total cell count. The apoptotic cell rate was determined by calculating the ratio of apoptotic cells to total cells in five different fields per group, expressed as a percentage: apoptotic cell rate = (number of apoptotic cells/total cell count) × 100%.

### In vivo animal experimentation

In this animal study, female non-obese diabetic/severe combined immunodeficiency (NOD-SCID) mice aged 6–8 weeks (obtained from Huxley-Janda Experimental Animals Co., Ltd, Hunan, China) were utilized to establish a metastatic tumor model by directly injecting 1 × 10^7^ pretreated and co-cultured A549/H1703 cells into the left ventricle of the mouse heart. The specific procedure involved anesthetizing the mice, making two transverse incisions towards the axilla to create a “Y” shaped incision, and accessing the thoracic cavity. Gently displacing the lung tissue revealed the heart, where a cell suspension was slowly injected into the left ventricle using a micro syringe or injection needle [42]. To monitor tumor growth in real time, tumor growth was monitored promptly and documented photographically, and mice forming bone metastatic lesions were selected for further experiments. Tumor volume was assessed every other day, and 1 × 10^5^ human peripheral blood mononuclear cells (hPBMCs; PCS-800-011, ATCC, USA) were intravenously injected via the tail vein (Fig. S2). On days 13 and 23, flow cytometry was employed to measure the concentration of human CD45 in the mouse blood [42–44].

Bioluminescence imaging data were acquired using the in vivo imaging system (IVIS) Spectrum CT system (PerkinElmer, USA). Micro-CT data were obtained utilizing the vivaCT 80 system (Scanco, Switzerland). For the EdU labeling assay, each mouse was intraperitoneally injected with 100 μg of EdU (C10640, Thermo Fisher, USA) 24 h prior to bone retrieval. The Click Plus EdU 647 imaging kit (C10419, Thermo Fisher, USA) was employed for EdU staining. Tumor cells were stained with GFP antibody, and the ratio of EdU-positive cells to GFP-positive cells was calculated [45].

The mice were randomly divided into 22 groups, each consisting of 6 mice: (1) PBS group; (2) TSRP group; (3) DMSO + PBS group; (4) SC79 + PBS group; (5) SC79 + TSRP group; (6) Blank group; (7) EVs group; (8) TMTP1-EVs group; (9) TSRP + DIR group; (10) TSRP-EVs group; (11) TMTP1-TSRP-EVs group. Each group was injected with A549 cells or H1703 cells for modeling. When the diameter of the metastatic tumor reached 100 mm^3^, 80 µL of PBS/TSRP (14 mg/kg) or 100 µL of 100 µg EVs labeled with DIR (40757ES25, YEASEN, China) was intravenously injected into the mice [46, 47]. EVs were administered three times per week, while PBS/TSRP was injected every two days for a total of three injections. After injections, whole-body fluorescence imaging was conducted at 2, 4, 6, 8, and 24 h using a near-infrared dual-zone small animal IVIS (Photon). At 24 h post-injection, the liver, spleen, kidneys, heart, lungs, and tumors were dissected, and the fluorescence intensity of each organ or tissue was measured. Mice in the SC79 + PBS group and SC79 + TSRP group received intraperitoneal injections of a 10 mg/kg SC79 solution (an AKT pathway activator), while the DMSO + PBS group received DMSO solution, administered three times per week [48]. The biodistribution of EVs was monitored for 30 days using the Kodak imaging system, and DiR-labeled EVs were analyzed using the Kodak Image System to determine their distribution [49]. In studying the impact of primary immune checkpoint resistance, mice were treated with Nivolumab (10 mg/kg, three times a week) via tail vein injection [49, 50].

Intra-tumoral tissue imaging: The collected tumor tissue was subjected to ex vivo fluorescence imaging, fixed in 4% paraformaldehyde for 24 h, placed in a 15% sucrose PBS solution for 24 h until sedimentation, and then transferred to 30% sucrose for another 24 h until sedimentation. Subsequently, the tumor tissue was frozen and sliced into 20 μm sections, followed by staining with 1 mg/mL DAPI for 10 min at room temperature. After washing twice with PBS (pH 7.4), the sections were immediately examined under a laser scanning confocal microscope (LSM 700, Carl Zeiss Microscopy, Germany) [51, 52].

### Histological staining techniques

For Hematoxylin and Eosin (H&E) staining, tissue samples are first fixed and then sectioned. The paraffin is removed by slicing the wax blocks in xylene, followed by dehydration in 100%, 95%, and 70% ethanol, rehydration, and either mounting or rinsing with water. The prepared sections are immersed in Hematoxylin staining solution (H8070, Solarbio, Beijing, China) for 5–10 min at room temperature. Subsequently, the sections are quickly differentiated in 1% hydrochloric acid ethanol solution for 10 s, rinsed with distilled water, dehydrated in 95% ethanol, and placed in Eosin staining solution (G1100, Solarbio, Beijing) for 5–10 min. After standard dehydration, clearing, and mounting, the slides are observed under an optical microscope.

### Immunohistochemistry (IHC) staining

The antibody list is provided in Table S1. Mouse tumor tissue is fixed in 4% paraformaldehyde overnight, followed by embedding in paraffin and sectioning at a thickness of 4 μm. Deparaffinization is accomplished using xylene, and hydration is achieved through a series of ethanol washes (anhydrous ethanol, 95% ethanol, and 75% ethanol for 3 min each). Subsequently, the sections are subjected to antigen retrieval by boiling in 0.01 M citrate buffer for 15–20 min, followed by a 30-min room temperature incubation in 3% H_2_O_2_ to inactivate endogenous peroxidases. The sections are then treated with goat serum blocking solution, incubated at room temperature for 20 min, and excess fluid is removed. First, the primary antibodies are added and left to incubate at room temperature for 1 h, followed by washing with PBS. Following the addition of the primary antibody, the samples were incubated at room temperature for 1 h, washed with PBS, and then incubated with secondary IgG anti-rabbit antibody for 20 min at 37 °C. Subsequently, the samples were washed with PBS and incubated with streptavidin-peroxidase (SP) at 37 °C for 30 min, followed by another PBS wash. The DAB substrate (P0202, Beyotime Biotechnology Co., Ltd) was then added for 5–10 min for color development, followed by a 10-min water rinse to stop the reaction. Counterstaining with hematoxylin (C0107, Beyotime Biotechnology Co., Ltd) was performed for 2 min, followed by differentiation in a hydrochloric acid alcohol solution and a 10-min water rinse prior to dehydration in graded alcohols (xylene transparent) and sealing with 2–3 drops of neutral resin. For analysis, under a light microscope, five random high-power microscope fields were selected per slide, with 100 cells counted in each field to calculate the percentage of positive cells.

### Flow cytometry

Flow cytometry was used to detect the levels of macrophages and CD8^+^ T cells. Macrophages derived from THP-1 in a co-culture model were collected by centrifugation at 1200 × *g* for 5 min at 4 °C, followed by resuspension in staining buffer. Single-cell suspensions from macrophages or tumor tissues were incubated in the dark at 4 °C for 30 min, and cells were dissociated using StemPro™ Accutase™ (A1110501, Gibco, USA) cell dissociation reagent. The cell pellets were then centrifuged at 1000 × *g*/min for 5 min and washed twice with PBS. Subsequently, cells were resuspended in 100 μL of PBS and stained with antibodies from Table S2. After antibody incubation, cells were washed three times with PBS and promptly analyzed using a flow cytometer (Beckman, USA).

### High-throughput sequencing and analysis of mRNA in tumor tissues of the NSCLC mouse bone metastasis model

Tumor tissue samples from PI3K-mutated A549 mice metastasized to the bone in the PBS group and the TSRP group (6 mice each) were randomly selected. Total RNA was isolated from the 12 samples using the total RNA isolation kit (12183555, Invitrogen, USA), and the quantity of total RNA was quantified by measuring the OD value with a UV spectrophotometer (BioSpectrometer basic, Eppendorf, USA). The integrity of these total RNAs was assessed using agarose gel electrophoresis. High-quality total RNA was reverse transcribed into cDNA to construct RNA libraries, which were sequenced using Illumina’s NextSeq 500 platform. The raw image data obtained from sequencing was converted into raw reads through base calling. To ensure the quality of raw reads, cutadapt was used to remove sequencing adapter sequences and filter out low-quality sequences, resulting in “clean reads.” These clean reads were aligned to the human reference genome using Hisat2 software, and then gene expression was quantified using the R software package to generate a gene expression matrix.

The “limma” package in R was used to identify differentially expressed genes in the high-throughput sequencing data, with the criteria defined as |log2FC | > 1 & *p*-value < 0.05. A volcano plot was generated using the ggplot2 package, and a heatmap was created using the pheatmap package. Gene Ontology (GO) and Kyoto Encyclopedia of Genes and Genomes (KEGG) pathway enrichment analysis was performed using the Xiantao Academic Database (https://www.xiantaozi.com/). The CIBERSORT package was utilized to analyze the proportions of 22 immune cell types in the 12 samples, with a filtering condition of *p* < 0.05, to obtain the immune cell proportion matrix data for each tumor sample. Bar plots and violin plots were generated using the “vioplot” and “ggpubr” packages in R to illustrate the relationship between gene mutations and tumor invasion of the immune system.

### Isolation and identification of CSCs

The A549 cell line was cultured in a high-glucose DMEM medium (11965126, Gibco, USA) supplemented with 10% FBS. Spheroid-enriched (SE) cell lines derived from the aforementioned A549 cells were cultured under the same conditions. Two key elements of the enrichment method included employing adherent culture conditions and repeatedly selecting cells with anchorage-independent spheroid growth capability. When the monolayer culture reached 90% confluence, floating individual cells or spheroids were collected, resuspended, and immediately replated until subconfluency. After repeating this process eight times, the culture was enriched with spheroid-forming cells that remained suspended under adherent culture conditions, termed as stem cell-like cell lines. Subsequently, CSCs could be obtained by flow cytometry sorting for CD44 and CD133 double-positive cells.

### Isolation and preparation of EVs

When CSC fusion reached 80%–90% confluence, the supernatant was discarded, and cells were washed with 2× PBS. Subsequently, 25 mL serum-free IMDM medium (12440053, Gibco, USA) was added to each culture flask, and cells were further incubated for 48 h at 37 °C, 5% CO_2_ in a humidified atmosphere. The cell supernatants were collected into 50 mL centrifuge tubes and centrifuged at 300 × *g* for 10 min at 4 °C to remove cell debris. The supernatant was then transferred to another 50 mL centrifuge tube, and upon collection, immediate isolation of EVs was conducted. At 4 °C, the supernatant was centrifuged at 2000 × *g* for 20 min to transfer it to sterile tubes for use in an ultracentrifuge, followed by centrifugation at 16,500 × *g* for 30 min at 4 °C. The supernatant was transferred to ultracentrifuge tubes and centrifuged at 120,000 × *g* for at least 70 min at a fixed-angle rotor at 4 °C. The supernatant was thoroughly discarded. Subsequently, 1 mL of 4 °C PBS was added to each ultracentrifuge tube, and the pellet was resuspended using a micropipette. The solutions from the same group were mixed in the ultracentrifuge tube, followed by the addition of 4 °C PBS to exceed three-quarters of the tube volume. Centrifugation at 120,000 × *g* for 60 min at 4 °C was conducted to remove excess supernatant, and the pellet was resuspended again with sterile PBS, yielding CSC-derived EVs.

Using electroporation technology, TSRP was encapsulated into CSCs-EVs. The previously centrifuged CSCs-EVs pellet was resuspended in electroporation buffer containing 1.15 mM potassium phosphate (pH 7.2), 25 mM potassium chloride, and 21% OptiPrep working solution (D1556, Sigma–Aldrich, USA). The suspension of EVs was filtered through a 0.22 μm filter. Purified TSRP protein was then added to the EVs at a weight ratio of 1:5 to form EVs-TSRP complexes via electroporation using a Gene Pulser Xcell (Bio-Rad). Following electroporation, the EVs were centrifuged at 100,000 × *g*, 4 °C for 2 h, and the pellet was resuspended in cold PBS solution.

The TMTP1 peptide (sequence: NVVRQ) was synthesized by Xi’an Huachen Biotechnology Co., Ltd. using Fmoc chemistry synthesis on a solid-phase synthesizer. The peptide was purified using high-performance liquid chromatography, and its sequence and structure were confirmed by mass spectrometry. To enable targeted, specific delivery, the 5′-COOH-modified TMTP1 peptide was covalently linked to the amine groups on the surface of EVs via EDC-NHS coupling, facilitating surface functionalization of the EVs for targeted delivery.

To activate the carboxyl group of the TMTP1 peptide, a mixture of denatured (heated at 85–95 °C for 10 min) and re-natured (cooled to room temperature for 15 min) TMTP1 peptide (5 μM) was prepared with EDC (46 mg, 0.3 mmol; 22980, Thermo Scientific™, USA) and NHS (35 mg, 0.3 mmol; 24500, Thermo Scientific™, USA) for one hour. 1 mL of EV suspension (200 mg/mL) was added to DNase/RNase-free water. The stable NHS esters rapidly reacted with the amines present on the surface of EVs when gently stirred and left to incubate overnight at room temperature. To remove the unconjugated TMTP1 peptide, the reaction mixture was filtered through a 100 kDa cutoff Amicon centrifugal filter at 5000 × *g* for 30 min, followed by two washes with cold DNase/RNase-free water. Gel retardation assay confirmed the stability of the TMTP1-EVs complex, with free TMTP1 peptide as the positive control and wild-type EVs as the negative control.

The concentration of unconjugated TMTP1 peptide in the supernatant was quantified using the NanoDrop™ One UV-Visible spectrophotometer (840-317400, Thermo Scientific™, USA) at 260 nm after filtration. Subsequently, the conjugation efficiency of the TMTP1 peptide was calculated using the following formula: Conjugation efficiency of TMTP1 peptide (%) = (initial amount of TMTP1 peptide added - amount of unconjugated TMTP1 peptide in the supernatant) / initial amount of TMTP1 peptide added × 100.

The EVs were grouped as follows (Fig. S3): (1) EVs group (EVs derived from wild-type CSCs); (2) TSRP-EVs group (CSCs-derived EVs encapsulating TSRP); (3) TMTP1-EVs group (CSCs-derived EVs successfully conjugated with TMTP1 peptide on their surface); (4) TMTP1-TSRP-EVs group (CSCs-derived EVs encapsulating TSRP and successfully conjugated with TMTP1 peptide on their surface).

### Characterization of EVs

In order to identify the characteristics of the EVs under investigation, EVs were resuspended in RIPA lysis buffer (89901, Thermo Scientific™, Germany), and their specific markers, such as Alix, TSG101, and CD81, were detected using Western blot. The negative control protein marker used was calnexin. Antibodies used in this study included rabbit anti-Alix (ab275377, 1:1000), anti-TSG101 (ab133586, 1:1000), anti-CD81 (ab286173, 1:500), and anti-calnexin (ab92573, 1:20,000), all purchased from the UK-based company Abcam.

By utilizing Nanoparticle Tracking Analysis (NTA), the size and concentration of EVs were determined. The EV samples were suspended in PBS and then diluted 500 times with Milli-Q water. Subsequently, the diluted EVs were injected into the sample chamber of the NanoSight LM10 (Malvern) instrument using a sterile syringe, ensuring the absence of air bubbles and filling the chamber completely. The NanoSight LM10 is equipped with a 640 nm laser and a fluoroelastomer O-ring (Viton). The videos were analyzed using NanoSight version 2.3 software (NanoSight Ltd, Amesbury, UK) with a gain set at 6.0 and a threshold of 11 to capture the particle trajectories. The software generated concentration and size distribution profiles of the diluted samples, from which the original EVs’ concentration was calculated based on the dilution factor.

After centrifuging the resuspended EVs to form a pellet, the pellet was fixed in a fixative solution (2% paraformaldehyde, 2.5% glutaraldehyde) at 4 °C for 1 h. The fixed pellets underwent three washes with PBS (15 min each), followed by fixation in 1% osmium tetroxide for 1.5 h and another three washes with PBS (15 min each). The samples were dehydrated in a graded series of alcohols, infiltrated with epoxy resin overnight, embedded, and polymerized at 35 °C, 45 °C, and 60 °C for 24 h. After ultra-thin sectioning and lead-uranium staining, observation was conducted using a transmission electron microscope (JEM-1011; JEOL, Tokyo, Japan) at an accelerating voltage of 80 kV, with image capture performed using a side-mounted Camera-Megaview III (Soft Imaging System, Münster, Germany). Each experiment was repeated three times.

### Immunofluorescence staining to assess the uptake of EVs by NSCLC cells

EVs derived from CSCs were seeded into a 24-well plate, and Dil dye (C1036, Beyotime Biotechnology Co., Ltd., Shanghai) was added to 40 μg of EVs to achieve a final concentration of 25 μM. The mixture was then allowed to react for 30 min at room temperature, followed by rapid centrifugation to remove unbound dye. Subsequently, the cells were washed three times with PBS and fixed with 4% paraformaldehyde (AR1068, BOSTER Biological Technology Co., Ltd., Wuhan) for 30 min. Finally, the cell nuclei were stained with DAPI (4′,6-diamidino-2-phenylindole, C1005, Beyotime Biotechnology Co., Ltd., Shanghai) for 30 min, and the cells were imaged at 400× magnification using a BX53 fluorescence microscope equipped with a camera (Olympus). Image analysis was performed using ImageJ Pro Plus 6.0 software.

### Relative expression level of target genes detected by RT-qPCR

Total RNA was extracted from tissues or cells using Trizol reagent (15596026, Invitrogen, USA), and the concentration and purity of the total RNA were assessed at 260/280 nm using NanoDrop LITE (ND-LITE-PR, Thermo Scientific™, Germany). The extracted total RNA was reverse transcribed into cDNA using the PrimeScript RT reagent Kit with gDNA Eraser (RR047Q, TaKaRa, Japan). Subsequently, the expression of various genes was analyzed by RT-qPCR using SYBR Green PCR Master Mix reagents (4364344, Applied Biosystems, USA) and the ABI PRISM 7500 Sequence Detection System (Applied Biosystems).

The primers for the genes were synthesized by TaKaRa (Table S3), with GAPDH serving as the reference gene. The relative expression levels of the genes were analyzed using the 2^−ΔΔCt^ method, where ΔΔCt = (average Ct value of target gene in the experimental group - average Ct value of reference gene in the experimental group) - (average Ct value of target gene in the control group - average Ct value of reference gene in the control group). All RT-qPCR analyses were performed in triplicate.

### Western blot

Initially, cells or tissues were collected and lysed using an enhanced RIPA lysis buffer containing protease inhibitors (P0013B, Beyotime Biotechnology Co., Ltd, Shanghai, China). Subsequently, the protein concentration was determined using a BCA protein quantification assay kit (P0012, Beyotime Biotechnology Co., Ltd, Shanghai, China). The proteins were separated by 10% SDS-PAGE and transferred to a PVDF membrane (FFP39, Beyotime Biotechnology Co., Ltd). The membrane was then blocked with 5% BSA (ST023, Beyotime Biotechnology Co., Ltd) at room temperature for 2 h to prevent nonspecific binding. After blocking, primary antibodies (rabbit anti-human, detailed information in Table S4) were added at a diluted concentration and incubated at room temperature for 1 h. Following primary antibody incubation, the membrane was washed and incubated with an HRP-conjugated goat anti-rabbit secondary antibody (ab6721, 1:2000, Abcam, UK) at room temperature for 1 h. Pierce™ ECL Western blot substrate (32209, Thermo Scientific™, Germany) A and B solutions were mixed in equal amounts in a darkroom; the mixture was then added onto the membrane, and the membrane was exposed in a gel imager. The Western blot images were captured using the Bio-Rad imaging system (BIO-RAD, USA), and the bands of interest were quantified for grayscale using ImageJ analysis software, with GAPDH and β-Tubulin as internal references. Each experiment was conducted in triplicate. All the Full and uncropped Western blot images can be found in the Supplementary Materials.

### Statistical analysis

Our study utilized R language, version 4.2.1, for statistical analysis. The R code was compiled using the RStudio integrated development environment, version 2022.12.0-353. For data processing, we employed the Perl language, version 5.30.0. Additionally, GraphPad Prism software, version 8.0, was utilized for analysis.

We represented continuous data as mean ± standard deviation. To compare data between the two groups, we conducted an independent samples *t*-test. For comparing data among multiple groups, one-way analysis of variance (ANOVA) was employed, and for comparing data across different time points within groups, two-way ANOVA was utilized. Bonferroni post-hoc tests were performed. The significance threshold was set at *p* < 0.05.

## Results

### PIK3CA mutation may be a potential immunogenic mutation in NSCLC

Bone metastasis is a common and fatal complication in patients with NSCLC, leading to severe bone pain, pathological fractures, and spinal cord compression [53]. In recent years, ICIs, such as anti-PD-1 and anti-PD-L1 antibodies, have become important therapeutic modalities for treating NSCLC. However, despite some patients showing a significant clinical response to ICI therapy, a considerable proportion still exhibit primary resistance, that is, no response to initial treatment [54]. Therefore, the development of new strategies to reduce bone metastasis and overcome primary immune checkpoint resistance holds great significance for the overall prognosis of NSCLC patients.

**Fig. 1:**
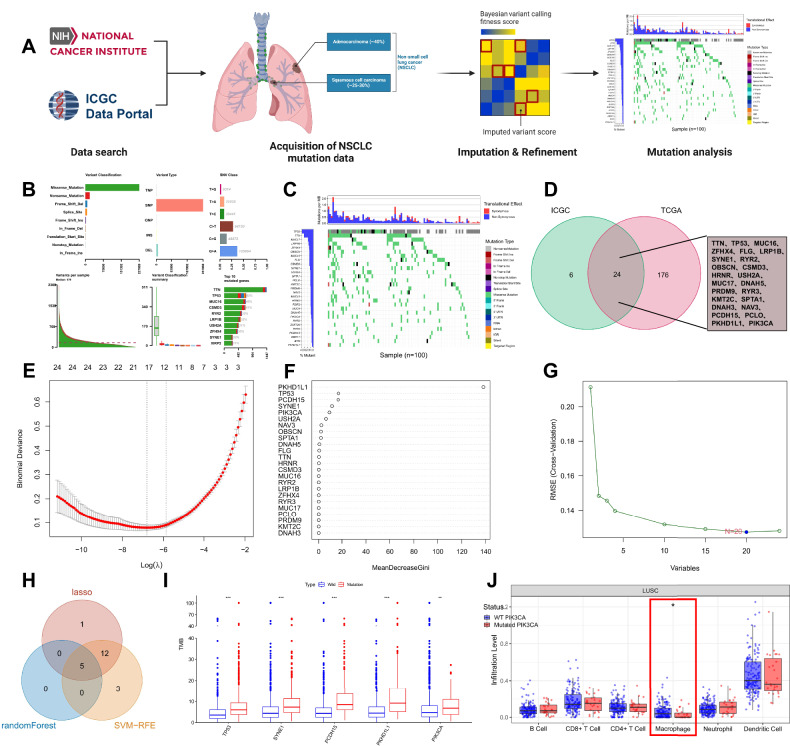
Analysis of Mutation Data in NSCLC from TCGA and ICGC for LUAD and LUSC patients. **A** Bioinformatics analysis flowchart for Fig. 1 (Created with BioRender.com); **B** The comprehensive figure displaying the mutation classification, mutation types, and SNV classification in TCGA-NSCLC samples (N = 1100); **C** Waterfall plot of genes with high mutation frequencies in ICGC-LUSC samples displayed on the left panel, where genes are sorted by mutation frequencies. Different mutation types are shown on the right panel, with a total sample size of 100; **D** Intersection of the top 200 mutation genes in TCGA-NSCLC samples and the top 30 mutation genes in ICGC-LUSC samples; **E** Lasso coefficient selection plot, showing the bias variation of the model under different λ (regularization parameter) values, with numbers below the curve indicating the number of non-zero coefficients in the model at the corresponding λ value. Optimal λ value selection is typically at the point where the curve is most stable, striking a balance between minimizing bias and model complexity (number of non-zero coefficients); **F** Random forest algorithm results plot, where each point represents a feature gene, and its position indicates the importance of the feature gene in the random forest model, measured by MeanDecreaseGini. MeanDecreaseGini is a metric that assesses the quality of a feature split, with higher values indicating greater importance of the feature in the model; **G** SVM-RFE analysis results plot, showing how the prediction error of the model (represented by root mean square error, RMSE) changes with the reduction in the number of features (denoted on the x-axis as the number of features). A decrease in RMSE signifies an improvement in the model prediction accuracy, with the optimal number of features typically identified by locating the point where RMSE starts to significantly increase; **H** Venn diagram displaying the intersection of NSCLC-related mutation genes identified by Lasso regression, random forest algorithm results, and SVM-RFE among the three machine learning algorithms; **I** Differential expression analysis of 5 mutation genes and TMB in TCGA-NSCLC (N = 1100); **J** Analysis of the level of PIK3CA gene mutations and differences in 6 immune cells using the TIMER database (N = 549). In the figures, * indicates *p* < 0.05, ** indicates *p* < 0.01, and *** indicates *p* < 0.001.

To delve into the genetic mutation landscape of NSCLC, we downloaded the somatic mutation profiles of LUAD and LUSC patients from the TCGA and ICGC databases and visualized them using the “maftools” package (Fig. 1A). The results revealed the top 200 genes in 1100 NSCLC samples from the TCGA database, as depicted in the figure (Fig. S4). By utilizing the tcgaCompare function to compare the mutational burden of NSCLC samples (LUAD and LUSC) with that of 31 other cancer types in the TCGA database, we observed a significantly higher number of mutations in NSCLC samples compared to other cancers (Fig. S5A). In NSCLC samples, missense mutations were the predominant mutation type, with SNPs occurring more frequently than insertions or deletions (Fig. 1B, Fig. S5B). Subsequently, we obtained and analyzed the genetic mutation data of 100 Korean patients with LUSC from the ICGC database and visualized it (Fig. S5C), illustrating the top 30 genes in the mutation profile (Fig. 1C). The intersection of the top 200 mutated genes in the TCGA dataset with the top 30 mutated genes in the ICGC database revealed 24 common genes (Fig. 1D), which exhibited notably high mutation frequencies in both datasets. Therefore, in the ensuing analysis, we focused on these particular mutated genes.

Subsequently, we conducted a multivariable Cox study using LASSO regression on these 24 genes and identified 18 feature genes specific to NSCLC (Fig. 1E). Simultaneously, we assessed gene importance through a random forest algorithm (Fig. 1F) and extracted feature genes using the SVM-RFE analysis method (Fig. 1G). Ultimately, we identified 5 critical mutated genes: PIK3CA, TP53, SYNE1, PCDH15, and PKHD1L1 (Fig. 1H). Furthermore, we calculated the TMB of NSCLC samples from TCGA and analyzed the relationship between these 5 mutated genes and TMB. The results indicated a significant difference in TMB elevation associated with mutations in PIK3CA in TCGA-NSCLC samples (Fig. 1I). Within NSCLC, mutations in the PI3K signaling pathway are common genetic alterations, with PIK3CA being the most frequently mutated gene in the pathway (Fig. S5D, E). Mutations in the PI3K pathway activate downstream AKT and mTOR signaling pathways, promoting cell proliferation, survival, and migration, consequently driving tumor progression and metastasis [55, 56]. Analysis from the COSMIC database revealed missense mutations as the primary mutation type for PIK3CA, with G > A being the most common single-nucleotide variant (SNV) (Fig. S5F).

To investigate the relationship between PIK3CA mutation and tumor-infiltrating immune cells in the NSCLC microenvironment, we utilized the TIMER database for immune cell infiltration analysis. The results revealed a significant decrease in macrophage immune cell infiltration in the PIK3CA-MUT group (Fig. 1J). The immune infiltration analysis suggests that the PIK3CA mutation may suppress macrophages. In conclusion, we identified one mutation gene associated with NSCLC: PIK3CA, and found a significant correlation between this gene mutation and TMB. The PIK3CA mutation in NSCLC is significantly associated with macrophage infiltration.

### PI3K mutation promotes bone metastasis of NSCLC and leads to primary immune checkpoint resistance

The role of PI3K mutation in bone metastasis of NSCLC has attracted widespread attention. Studies have shown that the activation of the PI3K signaling pathway not only promotes the bone-directed metastasis of tumor cells but also enhances the survivability of tumor cells by reshaping the TME [57, 58]. Moreover, abnormal activation of the PI3K/AKT signaling pathway can elevate the expression of PD-L1, weakening the efficacy of ICIs [59], which is a key factor contributing to primary resistance to ICIs [60].

**Fig. 2:**
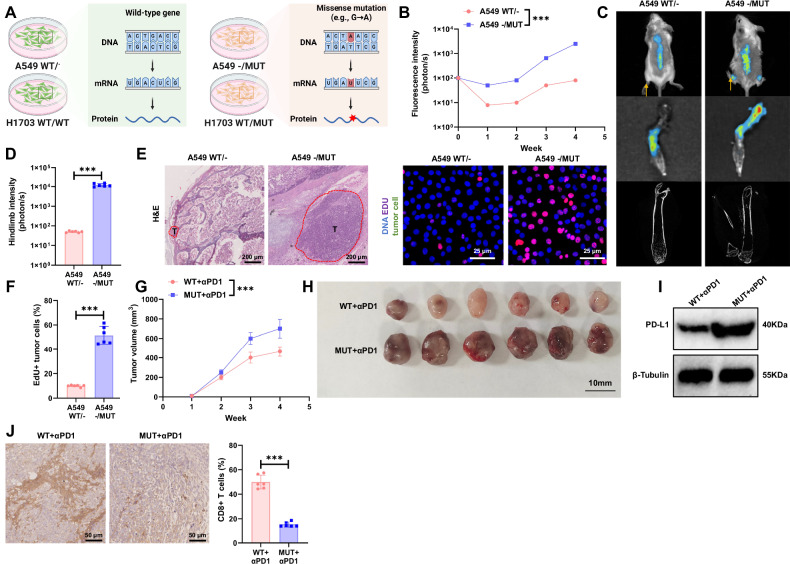
PIK3CA mutation promotes bone metastasis in the A549 xenograft model and induces primary immune checkpoint resistance. **A** Schematic representation of constructing PIK3CA-E545K heterozygous and homozygous knock-in mutants (Created with BioRender.com); **B** Fluorescence intensity results in NSCLC tumor tissues of mice in each group; **C** Bioluminescence imaging results of NSCLC tumors and metastatic lesions in vivo in mice of each group (arrows point to areas of bone damage); **D** Fluorescence intensity results of NSCLC tumor metastasis to hind limb tumor tissues in mice of each group; **E** H&E staining of tumor tissue and EdU immunofluorescence imaging results of tumor cells, scale bar = 200 μm; **F** Statistical results of EdU experiment; **G** Statistical results of tumor size in PIK3CA-mutant NSCLC xenograft mice after Nivolumab treatment; **H** Morphology of tumors in PIK3CA-mutant NSCLC xenograft mice after Nivolumab treatment; **I** Detection of PD-L1 protein expression levels in tumor tissues of PIK3CA-mutant NSCLC xenograft mice after Nivolumab treatment; **J** IHC detection of CD8^+^ T cell infiltration levels in tumor tissues of PIK3CA-mutant NSCLC xenograft mice after Nivolumab treatment, scale bar = 50 μm. Cell experiments were repeated at least three times, with 6 mice in each group, *** represents *p* < 0.001.

The ICGC database indicates that E545K is the most common mutation site in PIK3CA in NSCLC (Fig. S6A). Literature research also confirms this point [61–63], and mutations at the E545K site of PIK3CA are known to lead to tumor drug resistance [64]. Therefore, we have chosen the PIK3CA-E545K mutation as a focal point of our study. To investigate the impact of PI3K mutations on bone metastasis of NSCLC and primary immune checkpoint resistance, we utilized the CRISPR/Cas9 editing system to, respectively, construct PIK3CA-E545K heterozygous and homozygous knock-in mutants in A549 or H1703 cells (Fig. 2A).

To confirm the PIK3CA mutations in these cell lines, we performed Sanger sequencing of exon 9 of the PIK3CA gene isolated from the genomic DNA of A549 PIK3CA WT/^−^ (A549 WT/^−^), A549 PIK3CA^−^/MUT (A549^−^/MUT), H1703 PIK3CA WT/WT (H1703 WT/WT), and H1703 PIK3CA WT/MUT (H1703 WT/MUT) cells. In the A549 heterozygous allele cells, a single G to A transition in the codon was detected in A549^−^/MUT cells. In the H1703 allele cells, both G and A were detected in the codon of H1703 WT/MUT cells, indicating heterozygosity of the mutant allele (Fig. S6B).

To determine the correlation between copy number variations and point mutations of PIK3CA after mutation, we conducted FISH and RT-qPCR experiments to assess the copy number and mRNA expression levels of PIK3CA in mutant and wild-type groups. FISH results indicated an increase in the copy number of PIK3CA in both wild-type and mutant NSCLC cells, but this increase was not directly associated with the occurrence of a PIK3CA mutation (Fig. S6C). In contrast, the RT-qPCR results demonstrated no significant difference in the mRNA expression levels of PIK3CA between the wild-type and mutant groups (Fig. S6D).

To further investigate the impact of PIK3CA on bone metastasis of NSCLC, we established a metastatic tumor mouse model by intracardiac injection of wild-type and mutant PIK3CA NSCLC cells. By measuring human CD45 levels in the blood of mice 13 and 23 days after hPBMC injection, we observed that the protein levels of human CD45 in the hPBMC injection group increased by more than 30% after 23 days, indicating the successful construction of a humanized mouse model (Fig. S7A, B). The presence of a PIK3CA mutation significantly accelerated bone metastasis, leading to aggravated bone damage (Fig. 2B–F, Fig. S7C–G). Furthermore, when treating mice with the ICI Nivolumab (αPD-1), we found that the efficacy of Nivolumab in inhibiting tumor growth was markedly lower in the PIK3CA-mutant group compared to the wild-type group (Fig. 2G, H, Fig. S6E, F). Western blot and IHC results revealed that compared to the wild-type group, tumor specimens from mice carrying the PIK3CA-E545K point mutation exhibited higher levels of PD-L1 protein and less infiltration of CD8^+^ T cells (Fig. 2I, J, Fig. S6G, H).

These findings collectively confirm that PI3K mutations can promote bone metastasis of NSCLC and lead to primary resistance to ICIs.

### TSRP suppresses the PI3K/Akt/mTOR pathway to inhibit bone metastasis of NSCLC caused by PI3K mutation

**Fig. 3:**
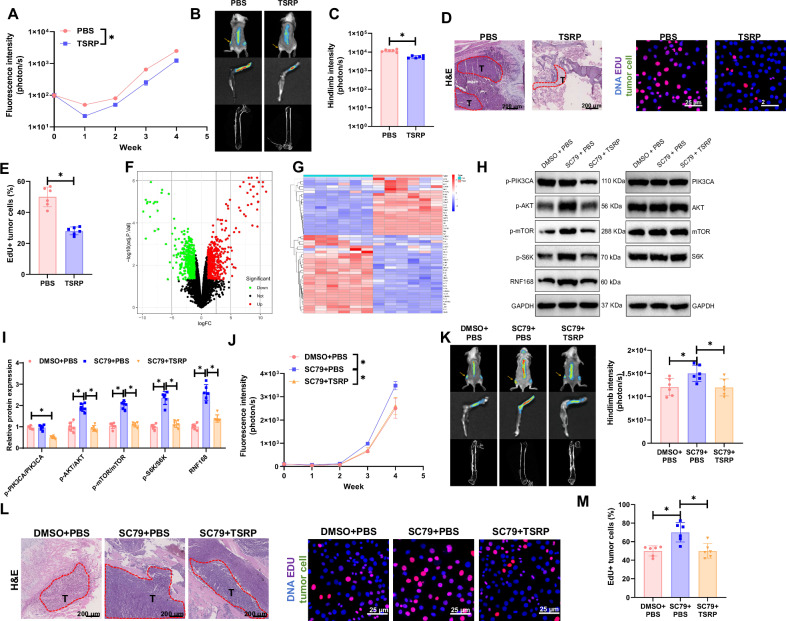
TSRP suppresses the PI3K/Akt/mTOR pathway to regulate bone metastasis in the A549 xenograft model with PIK3CA mutations. **A** Fluorescence intensity results in NSCLC tumor tissues of mice in each group; **B** Bioluminescence imaging results of NSCLC tumors and metastatic lesions in vivo in mice of each group (arrows point to areas of bone damage); **C** Fluorescence intensity results of NSCLC tumor metastasis to hind limb tumor tissues in mice of each group; **D** H&E staining of tumor tissue and EdU immunofluorescence imaging results of tumor cells, scale bar = 200 μm; **E** Statistical results of EdU experiment; **F**, **G** Volcano plot and heatmap of transcriptome sequencing differential analysis of tumor tissues metastasized to bones in mice from the PBS group (N = 6) and TSRP group (N = 6); **H**, **I** Detection results of protein phosphorylation levels of the PI3K/Akt/mTOR pathway in tumor tissues metastasized to bones in mice of each group; **J** Fluorescence intensity results in NSCLC tumor tissues of mice in each group; **K** Bioluminescence imaging results of NSCLC tumors and metastatic lesions in vivo in mice of each group (arrows point to areas of bone damage) and fluorescence intensity results of hind limb tumor tissues; **L** H&E staining of tumor tissue, scale bar = 200 μm and EdU immunofluorescence imaging results of tumor cells, scale bar = 25 μm; **M** Statistical results of EdU experiment. Each group consisted of 6 mice, * represents *p* < 0.05.

Previous studies have shown that the use of TSRP can achieve a comprehensive, synergistic therapy combining anti-tumor, anti-angiogenic, and immune responses [35]. Thus, we hypothesized that TSRP could also be effective in treating bone metastasis of NSCLC driven by PI3K mutation. To test this hypothesis, we initially characterized the purchased TSRP, and the results of HPLC and turbidity tests were consistent with the literature [35] (Fig. S8A, B). Subsequently, we treated a bone metastasis mouse model with TSRP via tail vein injection, and the results showed that compared to the PBS group, the TSRP treatment group exhibited reduced bone metastatic burden and bone resorption of NSCLC cells (Fig. 3A–E, Fig. S8C–G).

Furthermore, we conducted transcriptome sequencing analysis on the tumor tissues that had metastasized to the bones of mice in the A549 xenograft model treated with PBS and TSRP and identified a total of 22 upregulated genes and 30 downregulated genes (Fig. 3F, G). The results of the GO pathway enrichment analysis revealed that the highly expressed differentially regulated genes were mainly involved in biological processes such as leukocyte proliferation, positive regulation of T cell proliferation, and positive regulation of lymphocyte proliferation, whereas the downregulated genes were enriched in biological processes including response to peptide, regulation of vasculature development, and regulation of angiogenesis (Fig. S8H, J). Moreover, the KEGG pathway enrichment analysis showed that the upregulated differentially regulated genes were predominantly enriched in pathways such as Natural killer cell mediated cytotoxicity, T cell receptor signaling pathway, and Cytokine-cytokine receptor interaction, while the downregulated genes were mainly enriched in pathways including PD-L1 expression and PD-1 checkpoint pathway in cancer, PI3K-Akt signaling, and mTOR signaling pathway (Fig. S8I, K).

The pathway enrichment analysis results indicate that the inhibition of bone metastasis of NSCLC by TSRP may be associated with the downregulation of the PI3K/Akt/mTOR pathway. Consequently, upon intraperitoneal injection of the Akt pathway activator SC79 in mice, we observed that, compared to the control group, the phosphorylation levels of PI3K showed no significant change in the mice treated with the Akt pathway activator. However, there was a significant increase in the phosphorylation levels of Akt and mTOR, leading to a significant increase in the bone metastatic burden and bone resorption of NSCLC cells. Subsequently, the addition of TSRP reversed these effects, with a significant decrease in the phosphorylation levels of PI3K. S6K and RNF168, common downstream factors in the PI3K/Akt/mTOR pathway that influence tumor proliferation and migration, were significantly upregulated in mice treated with the AKT activator [16, 65]. S6K phosphorylation and RNF168 protein levels increased significantly, while TSRP treatment reversed these changes. (Fig. 3H–M, Fig. S9A–F). These results demonstrate that TSRP can inhibit the PI3K/Akt/mTOR pathway to prevent bone metastasis of NSCLC driven by PI3K mutation.

### TSRP reshapes the TME of PI3K-mutant NSCLC

**Fig. 4:**
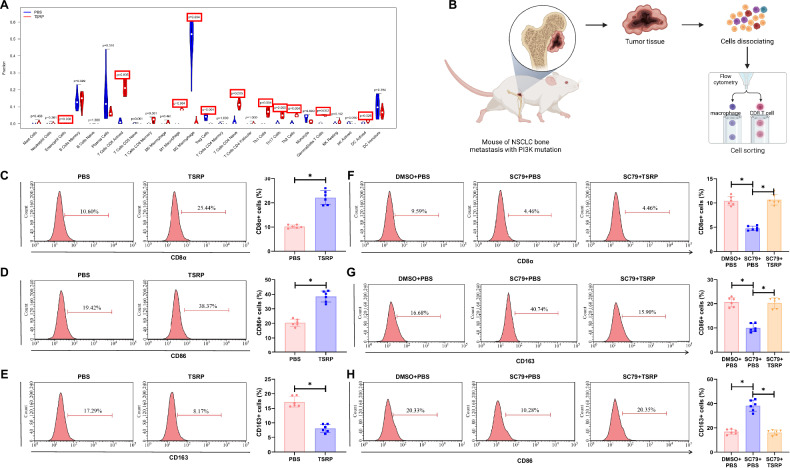
TSRP remodels the tumor microenvironment in PI3K-mutant A549 xenograft mice. **A** Immune infiltration analysis in tumor tissues metastasized to bones of mice in the PBS group and TSRP group; **B** Schematic representation of the impact of TSRP on the NSCLC tumor immune microenvironment validated through experiments (Created with BioRender.com); **C**–**E** Analysis of the effect of TSRP on the content of CD8^+^ T cells and macrophage subtypes in tumor tissues of each group using flow cytometry; **F**–**H** Analysis of the impact of TSRP-mediated PI3K/Akt/mTOR pathway on the content of CD8^+^ T cells and macrophage subtypes in tumor tissues of each group using flow cytometry. Each group consisted of 6 mice, * represents *p* < 0.05.

Bone metastasis is determined not only by the intrinsic characteristics of cancer cells but is also significantly influenced by the TME. The TME, consisting of cancer cells, blood vessels, and immune cells, orchestrates the complex interactions that regulate tumor growth, invasion, and metastasis [66]. Immunoinfiltration analysis of the transcriptome sequencing data revealed that in the TSRP group, the levels of infiltrating T Cells CD8 Activated (*p* = 0.005) and M1 Macrophages (*p* = 0.004) in the tumor tissue metastasized to the bone in mice were significantly increased, while the levels of M2 Macrophages (*p* = 0.004) were significantly decreased. Furthermore, most immune cells showed a positive correlation, suggesting an activation of the immune response in the tumor tissue of mice after TSRP treatment and a potential synergy among immune cells (Fig. 4A, Fig. S10A, B). Existing literature has confirmed that CD8^+^ T cells and M1 macrophages can inhibit bone metastasis of lung cancer, whereas M2 macrophages have the opposite effect [67, 68].

To further investigate the impact of TSRP on the infiltration levels of macrophages and CD8^+^ T cells in vivo, we used flow cytometry to measure the content of CD8^+^ T cells and macrophages in the tumor tissue (Fig. 4B). The results indicated that compared to the PBS group, the TSRP group exhibited a significant increase in the infiltration levels of CD8^+^ T cells and M1 macrophages, while the infiltration level of M2 macrophages was markedly decreased (Fig. 4C–E, Fig. S10C–E).

Furthermore, upon intraperitoneal injection of SC79 in mice, we observed a significant downregulation in the infiltration levels of M1 macrophages and CD8^+^ T cells and an upregulation in M2 macrophage infiltration levels in the SC79 injection group compared to the DMSO + PBS group. However, when TSRP was added, the infiltration levels of M1 macrophages and CD8^+^ T cells were significantly upregulated, while the infiltration levels of M2 macrophages were notably decreased (Fig. 4F–H, Fig. S10F–H). These results suggest that TSRP can reshape the TME by inhibiting the PI3K/Akt/mTOR pathway. By enhancing M1 macrophage and CD8^+^ T cell infiltration and reducing M2 macrophage infiltration, TSRP reshapes the tumor microenvironment in mice, potentially enhancing anti-tumor immune responses and providing new insights for tumor therapy.

### Reversal of primary immune checkpoint resistance in NSCLC with PI3K mutation by TSRP

**Fig. 5:**
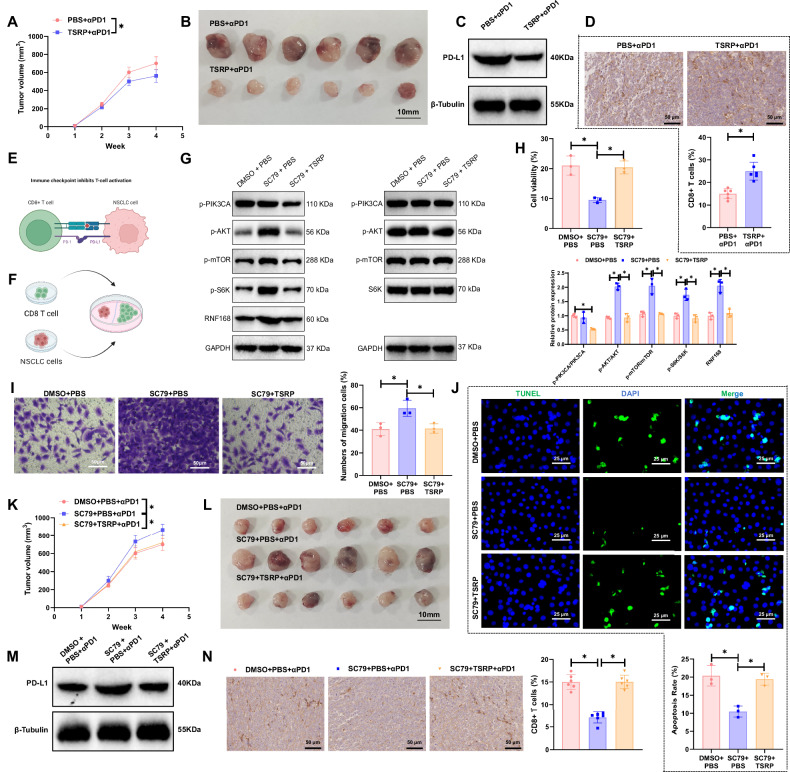
TSRP inhibits the PI3K/Akt/mTOR pathway to regulate primary immune checkpoint resistance in PI3K-mutant A549 xenograft models. **A** Tumor size statistics of PIK3CA-mutant NSCLC xenograft mice treated with Nivolumab; **B** Morphology of tumors in PIK3CA-mutant NSCLC xenograft mice after Nivolumab treatment; **C** Western blot analysis of PD-L1 protein expression levels in tumor tissues from PIK3CA-mutant NSCLC xenograft mice treated with Nivolumab; **D** IHC examination of CD8^+^ T cell infiltration levels in tumor tissues of PIK3CA-mutant NSCLC xenograft mice after Nivolumab treatment, scale bar = 50 μm; **E** Schematic diagram of the mechanism of PD-1/PD-L1 involvement in ICIs resistance (Created with BioRender.com); **F** Schematic diagram of co-culture cell model of NSCLC cell lines with CD8^+^ T cells (Created with BioRender.com); **G** Western blot analysis of protein phosphorylation levels in the PI3K/Akt/mTOR pathway in co-culture cell models from different groups; **H** Detection results of CD8^+^ T cell activity in various co-culture cell models; **I** Detection results of migration capability of tumor cells in various co-culture cell models, scale bar = 50 μm; **J** Detection results of apoptosis status of tumor cells in various co-culture cell models, scale bar = 25 μm; **K** Tumor size statistics of PIK3CA-mutant NSCLC xenograft mice after Nivolumab treatment; **L** Morphology of tumors in PIK3CA-mutant NSCLC xenograft mice after Nivolumab treatment; **M** Detection of PD-L1 protein expression levels in tumor tissues of PIK3CA-mutant NSCLC xenograft mice after Nivolumab treatment; **N** IHC examination of CD8^+^ T cell infiltration levels in tumor tissues of PIK3CA-mutant NSCLC xenograft mice after Nivolumab treatment, scale bar = 50 μm. Cell experiments were repeated at least three times, with 6 mice in each group, and * represents *p* < 0.05.

Numerous studies have indicated that the PI3K/AKT/mTOR signaling pathway plays a crucial role in the onset and progression of various cancers. This pathway can upregulate the expression levels of PD-L1 in NSCLC and has a close association with primary immune checkpoint resistance [15, 69, 70]. TSRP has been shown to enhance the infiltration levels of CD8^+^ T cells [35]. Comparative analysis of the treatment outcomes in mice from the PBS and TSRP groups revealed that the therapeutic efficacy of Nivolumab in the TSRP group was significantly superior to that in the PBS group. Furthermore, there was a significant reduction in the protein levels of PD-L1 and an increase in CD8^+^ T cell infiltration in the TSRP group, suggesting that TSRP may exert its effect in reversing primary immune checkpoint resistance by inhibiting PD-L1 expression and enhancing the infiltration levels of CD8^+^ T cells (Fig. 5A–E, Fig. S11A–D).

To further investigate the mechanism by which TSRP reverses primary immune checkpoint resistance, we established an in vitro co-culture cell model of NSCLC cell lines with CD8^+^ T cells (Fig. 5F). In this cell model, SC79 or (and) TSRP was introduced. In comparison to the DMSO + PBS group, the SC79 + PBS group showed no significant change in the phosphorylation levels of PI3K in tumor cells; however, there was a significant increase in the phosphorylation levels of AKT and mTOR proteins, as well as the expression of PD-L1 protein. Moreover, there was a notable decrease in the proliferation capacity of CD8^+^ T cells, an increase in the migration ability of tumor cells, and a reduction in apoptosis. When compared to the SC79 + PBS group, the SC79 + TSRP group exhibited a significant decrease in the phosphorylation levels of PI3K, AKT, and mTOR proteins, as well as the expression of PD-L1 protein. Additionally, there was a significant increase in the proliferation capacity of CD8^+^ T cells, a decrease in the migration ability of tumor cells, and an increase in apoptosis. Further examination of downstream factors of the PI3K/Akt/mTOR pathway revealed that, compared to the DMSO + PBS group, the SC79 + PBS group had significantly elevated phosphorylation levels of S6K and increased RNF168 expression. In contrast, the SC79 + TSRP group exhibited significantly reduced phosphorylation of S6K and decreased RNF168 expression (Fig. 5G, K, Fig. S11E–H). These results indicate that S6K and RNF168 are key downstream molecules in this pathway.

Upon further in vivo validation of the aforementioned in vitro experimental results, it was observed that the efficacy of Nivolumab in inhibiting tumor growth in the SC79 + PBS group was notably inferior to that in the DMSO + PBS group. However, the therapeutic effectiveness in the SC79 + TSRP group was significantly superior to that in the SC79 + PBS group. Additionally, IHC results revealed that compared to the DMSO + PBS group, the tumor specimens from the SC79 + PBS group exhibited higher levels of PD-L1 protein. In contrast, the PD-L1 protein levels were lower in the SC79 + TSRP group compared to the SC79 + PBS group (Fig. 5L–N, Fig. S11I–L). In conclusion, TSRP can reshape the TME by inhibiting the PI3K/Akt/mTOR pathway, thereby impeding bone metastasis of NSCLC with PI3K mutation and primary immune checkpoint resistance.

### Synthesis and characterization of TMTP1-TSRP-EVs

**Fig. 6:**
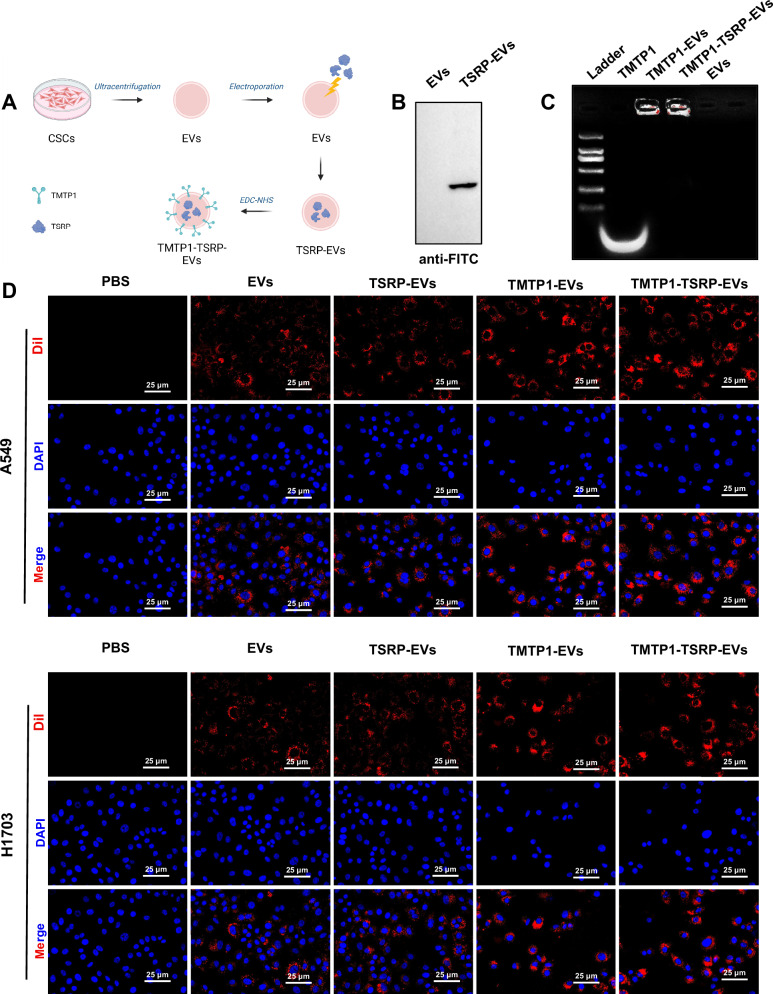
Construction and validation of TMTP1-modified CSC-derived EVs (CSCs-EVs) encapsulating TSRP. **A** Schematic diagram of the synthesis of TMTP1-TSRP-Evs (Created with BioRender.com); **B** Evaluation of the encapsulation efficiency of TSRP by EVs using anti-FITC antibody labeling under electroporation technique; **C** Detection of the coupling effect between TMTP1 peptide and exosomes using agarose gel electrophoresis; **D** Laser scanning microscopy observation of the uptake of EVs by A549 and H1703 cells, with red fluorescence representing Dil and blue fluorescence indicating DAPI nuclear staining (scale bar: 25 μm). Cell experiments were repeated at least three times.

We have investigated the fact that TSRP inhibits the PI3K/Akt/mTOR pathway to prevent bone metastasis of NSCLC with PI3K mutation and primary immune checkpoint resistance. In order to enhance the targeting specificity and stability of TSRP in tumor tissue, we propose to develop a nanomaterial capable of delivering TSRP specifically to NSCLC cells. EVs derived from CSCs have been reported to target tumor cells effectively [71], displaying good biocompatibility and low immunogenicity [72]. Therefore, engineered CSCs-derived EVs have garnered attention as vehicles for delivering RNA, proteins, and small-molecule drugs [34, 73]. The TMTP1 peptide exhibits strong and specific targeting to tumor tissues and metastatic lesions in mouse models, facilitating more precise payload delivery to tumor tissues and metastatic lesions while minimizing effects on normal tissues [74, 75]. Therefore, we chose CSCs-derived EVs to construct nanomaterials for delivering TSRP, simultaneously utilizing TMTP1 modification on EVs to enhance their targeting specificity, as depicted in Fig. 6A.

Initially, we enriched CSC spheres from A549 cells through a spheroid formation assay, observing their spherical shape and suspended growth under an optical microscope (Fig. S12A). Flow cytometry confirmed the enrichment of CSCs, with surface expression of the stem cell markers CD44 (95.60%) and CD133 (96.85%) on A549-CSCs (Fig. S12B). These results indicate the successful isolation of A549-CSCs.

Subsequently, EVs were isolated from A549-CSCs, and Western Blot analysis revealed the expression of classical EV markers (Alix, TSG101, CD81) in CSC-derived EVs (Fig. S12C). Transmission electron microscopy and NTA confirmed the typical cup-shaped morphology of EVs, ranging in size from 50 to 200 nm (Fig. S12D, E). Through electroporation, TSRP was encapsulated into EVs, and detection using FITC antibodies confirmed the presence of TSRP in the TSRP-EVs group, demonstrating the successful construction of CSCs-EVs encapsulating TSRP (Fig. 6B).

Subsequently, measurements of the free adapter in the filtered supernatant of the EVs post-incubation with the adapter aptamer indicated that approximately 56% of the initial adapter had attached to the EVs. The concentration of the adapter in the supernatant was 63.81 μg, while the original amount in the coupling reaction was 148.65 μg. By calculation, the amount of TMTP1 peptide attached to 200 μg of EVs was determined to be 84.84 μg. Furthermore, EVs successfully conjugated with the TMTP1 peptide exhibited a delay on 2.5% agarose gel electrophoresis. In comparison to the free TMTP1 peptide, the formation of a covalent bond between the carboxyl group at the 5’ end of the TMTP1 peptide and the amino groups on the surface of EVs increased the molecular weight of the conjugate complex, preventing its migration on the agarose gel (Fig. 6C). To further investigate the uptake of TSRP or the prepared EVs by NSCLC cells, the uptake was assessed by labeling the various EV groups with Dil dye and co-incubating them with A549 and H1703 cells for 24 h. Cell uptake of EVs was observed under a laser scanning microscope. The results showed that the PBS group exhibited no red fluorescence signal in NSCLC cells, while distinct red fluorescence signals were observed in the other 4 groups of cells. The TMTP1-EVs group and TMTP1-TSRP-EVs group displayed the strongest red fluorescence signals, potentially due to the targeted mechanism of TMTP1 enhancing the uptake rate by tumor cells (Fig. 6D). These findings indicate successful uptake of the various EV groups by NSCLC cells.

Based on the collective results, we have successfully constructed TMTP1-TSRP-EVs, which have demonstrated successful uptake by NSCLC cells.

### TMTP1-TSRP-EVs reversing primary immune checkpoint resistance in PI3K-mutant NSCLC

**Fig. 7:**
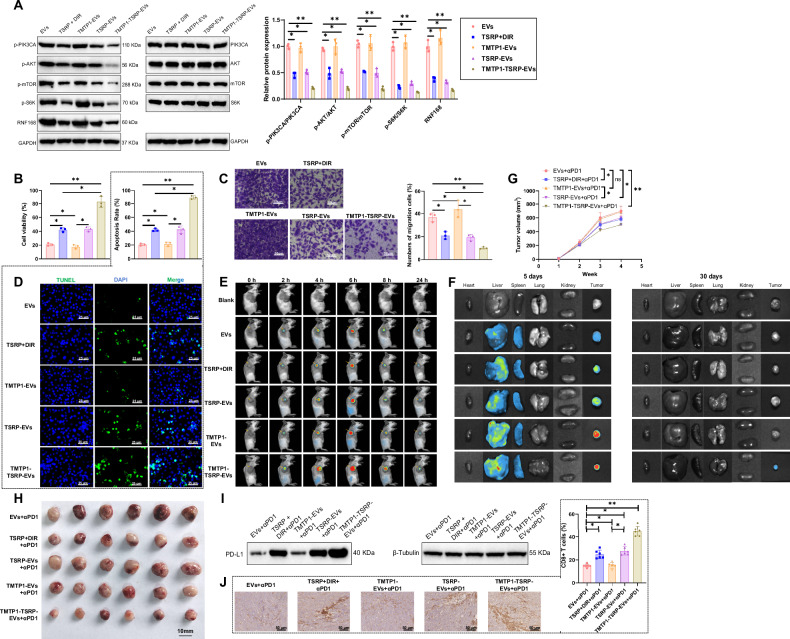
TMTP1-TSRP-EVs reverse immune checkpoint inhibitor (ICI) resistance in PI3K-mutant A549 xenograft mice. **A** Western blot analysis detected phosphorylation levels of the PI3K/Akt/mTOR pathway proteins in co-culture models; **B** Evaluation of CD8^+^ T cell activity in various co-culture cell models; **C** Assessment of tumor cell migration capability in various co-culture cell models, scale bar = 50 μm; **D** Analysis of apoptosis status of tumor cells in various co-culture cell models, scale bar = 25 μm; **E** In vivo NIR fluorescence imaging of fluorescence distribution in different groups of mice; **F** In vivo NIR fluorescence imaging of major organs and tumor tissues in different groups of mice; **G** Tumor size statistics of PIK3CA-mutant NSCLC xenograft mice after Nivolumab treatment; **H** Morphology of tumors in PIK3CA-mutant NSCLC xenograft mice after Nivolumab treatment; **I** Detection of PD-L1 protein expression levels in tumor tissues of PIK3CA-mutant NSCLC xenograft mice after Nivolumab treatment; **J** IHC examination of CD8^+^ T cell infiltration levels in tumor tissues of PIK3CA-mutant NSCLC xenograft mice after Nivolumab treatment, scale bar = 25 μm. Cell experiments were repeated at least three times with 6 mice in each group, and * represents *p* < 0.05.

To further investigate the impact of prepared TMTP1-TSRP-EVs on the primary immune checkpoint resistance in PI3K-mutant NSCLC, we co-cultured EVs from different groups with A549 cells and assessed their effects on the PI3K/AKT/mTOR pathway, PD-L1 protein, CD8^+^ T cells, and tumor cells. Compared to the control group (EVs) and TMTP1-EVs group, the TSRP-EVs group and TMTP1-TSRP-EVs group exhibited significantly reduced levels of phosphorylation of PI3K, AKT, and mTOR proteins, as well as decreased expression of PD-L1 protein. Additionally, there was a notable increase in the proliferative capacity of CD8^+^ T cells, decreased migration ability of tumor cells, and increased apoptosis, with the strongest effect observed in the TMTP1-TSRP-EVs group (Fig. 7A–D, Fig. S13A–D).

Subsequently, we intravenously injected EVs labeled with DIR into mice and evaluated the biodistribution of EVs from each group using near-infrared imaging. Live imaging was conducted within 24 h post-tail vein injection of EVs from each group, followed by ex vivo imaging of major organs and tumors 24 h later. The results revealed that within 24 h, the fluorescent signal of TSRP-EVs, TMTP1-EVs, and TMTP1-TSRP-EVs was primarily localized in the kidney, liver, and tumor tissues. Due to the non-targeted nature and smaller particle size of EVs in the EVs group, they were rapidly metabolized in the kidneys, resulting in a gradual decrease in accumulation at tumor sites. The metabolism rate of the TSRP + DIR group was only slightly lower than that of the EVs group. In contrast, TMTP1-EVs and TMTP1-TSRP-EVs exhibited prolonged retention of fluorescent signals at tumor sites, with the highest intensity observed in TMTP1-EVs and TMTP1-TSRP-EVs groups, attributed to the targeting capability of TMTP1, leading to extended accumulation time at tumor sites (Fig. 7E, F, Fig. S13E, F). Fluorescence tracking experiments showed that TMTP1-TSRP-EVs maintained sustained expression in tumor tissues for up to 30 days (Fig. 7F, Fig. S13F).

In vivo validation of the aforementioned in vitro experimental results revealed that the efficacy of tumor growth inhibition by the EVs group and TMTP1-EVs group with Nivolumab was significantly lower compared to the TSRP + DIR group, TSRP-EVs group, and TMTP1-TSRP-EVs group. Interestingly, the therapeutic effect of the TMTP1-TSRP-EVs group was notably superior to the TSRP + DIR group and the TSRP-EVs group. Furthermore, IHC results demonstrated that, in comparison to the EVs group and TMTP1-EVs group, the TSRP + DIR group, TSRP-EVs group, and TMTP1-TSRP-EVs group exhibited lower levels of PD-L1 protein and higher infiltration of CD8^+^ T cells, with the most pronounced effect observed in the TMTP1-TSRP-EVs group (Fig. 7G–J, Fig. S13G–J). These results collectively indicate that TMTP1-TSRP-EVs have the potential to reverse primary immune checkpoint resistance in PI3K-mutant NSCLC.

### TMTP1-TSRP-EVs reshape the TME and inhibit PI3K-mutant bone metastasis of NSCLC

**Fig. 8:**
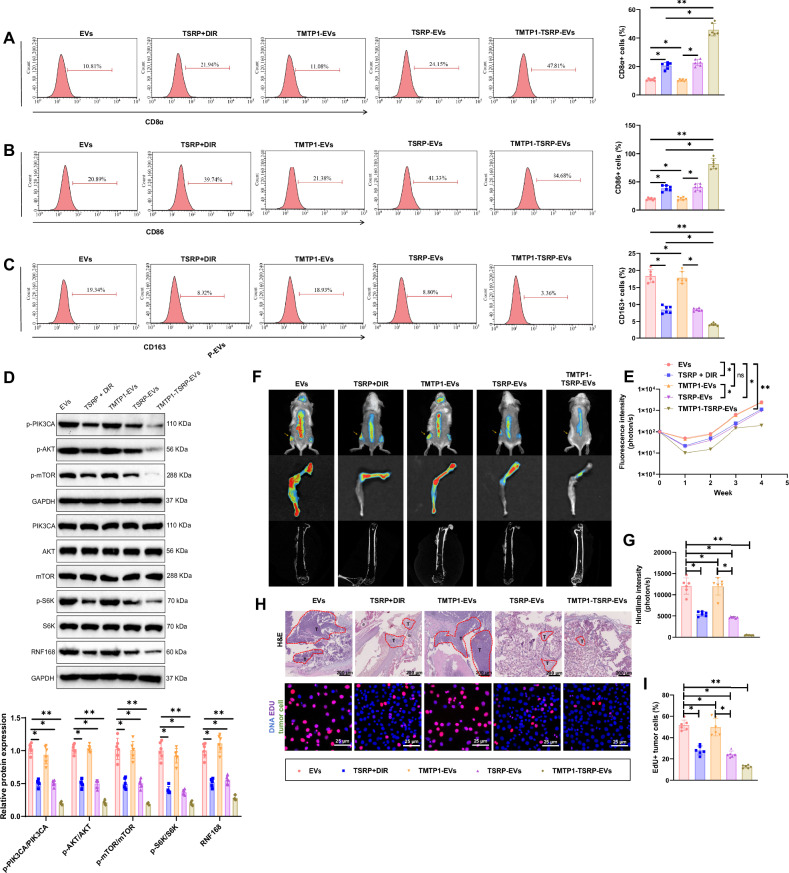
MTP1-TSRP-EVs remodel the tumor microenvironment and inhibit bone metastasis of PI3K-mutant A549 cells in vivo. **A**–**C** Flow cytometric analysis of the impact of TSRP on the content of CD8^+^ T cells and macrophage subtypes in tumor tissues of different groups; **D** Western blot analysis of phosphorylation levels of proteins in the PI3K/Akt/mTOR pathway in tumor tissues metastasized to bone from different groups of mice; **E** Fluorescence intensity of NSCLC tumor tissues in different groups of mice; **F** Bioluminescence imaging results of NSCLC tumors and metastatic lesions in different anatomical regions of mice (arrows indicate bone lesion areas); **G** Fluorescence intensity of tumor tissues in the hind limbs where NSCLC tumors metastasized; **H** H&E staining of tumor tissue and EdU immunofluorescence imaging of tumor cells, scale bar = 200 μm; **I** Statistical results of EdU experiments. *p < 0.05, **p < 0.01, with 6 mice in each group.

In this study, we conducted IHC analysis to evaluate the impact of TMTP1-TSRP-EVs on the TME in mice. The results demonstrate a significant increase in the infiltration levels of CD8^+^ T cells and M1 macrophages in the TSRP + DIR group, TSRP-EVs group, and TMTP1-TSRP-EVs group compared to the EVs group and TMTP1-EVs group. Conversely, the infiltration levels of M2 macrophages decreased notably. Moreover, the TMTP1-TSRP-EVs group exhibited a higher enrichment of CD8^+^ T cells and M1 macrophages compared to the TSRP + DIR group and TSRP-EVs group, and a weaker infiltration of M2 macrophages (Fig. 8A–C and Fig. S14A–C). TMTP1-TSRP-EVs reshape the tumor microenvironment by enhancing M1 macrophage and CD8^+^ T cell infiltration and reducing M2 macrophage infiltration. This remodeling enhances anti-tumor immune responses and provides new therapeutic insights for tumor treatment.

Furthermore, in comparison to the EVs group and TMTP1-EVs group, the mice in the TSRP + DIR group, TSRP-EVs group, and TMTP1-TSRP-EVs group exhibited significantly decreased phosphorylation levels of PI3K, Akt, and mTOR in tumor tissues near the bone. This led to the inhibition of tumor cell bone metastasis and alleviation of bone damage. Notably, the TMTP1-TSRP-EVs group showed the lowest pathway activation levels, resulting in the most significant suppression of bone metastasis (Fig. 8D–I and Fig. S14D–I).

Subsequently, after the completion of treatment, major organs were extracted for an H&E staining analysis, revealing no significant pathological changes (Fig. S15). This suggests that TMTP1-TSRP-EVs possess good biocompatibility and do not exhibit apparent toxicity to the organism. In conclusion, TMTP1-TSRP-EVs have the potential to reshape the TME, prevent PI3K-mutant bone metastasis of NSCLC, and counteract primary immune checkpoint resistance.

## Discussion

This study successfully demonstrated the potential of utilizing TMTP1-TSRP-EVs in preventing bone metastasis and reversing immune checkpoint resistance in PI3K-mutant NSCLC. Combining TSRP with TMTP1-modified CSCs-EVs enhanced their targeting specificity and therapeutic efficacy. In contrast to previous studies, peptide therapies showed lower targeting specificity and limited effectiveness in clinical applications. This study innovatively utilized EVs as carriers to improve drug stability and specificity, achieving precise targeting of tumor cells through TMTP1.

The findings of this study revealed that TSRP inhibited the proliferation and migration of PIK3CA-mutant NSCLC by downregulating the PI3K/Akt/mTOR signaling pathway. Prior research has indicated that PIK3CA mutation is a crucial factor in the malignant transformation of tumor cells and therapy resistance [64, 76, 77]. Through high-throughput sequencing and bioinformatics analysis, this study further confirmed the significant role of the PI3K/Akt/mTOR pathway in PIK3CA-mutant NSCLC. Unlike other studies, this research elaborated on the specific inhibitory effect of TSRP on this pathway, providing a more in-depth explanation of the molecular mechanism. Additionally, both in vitro and in vivo experiments validated the effectiveness of TSRP in preventing bone metastasis, laying a solid foundation for future clinical applications.

This study also found that TSRP significantly increased the infiltration of CD8^+^ T cells in the TME and suppressed the expression of PD-L1, thus reversing immune checkpoint resistance. Compared to immunomodulators used in previous studies, TSRP exhibited a stronger effect in regulating the immune microenvironment [35]. While prior research often focused on single immunomodulators, this study, through multi-layered experiments, demonstrated the multifaceted role of TSRP in reshaping the immune microenvironment. Specifically, using flow cytometry and IHC techniques, we detailed the impact of TSRP on immune cell infiltration and function, offering new perspectives for its application in immunotherapy.

The role of CSCs and their secreted EVs in the TME is gaining increasing attention [27, 28, 78]. Through spheroid formation assays and transmission electron microscopy, this study successfully isolated and identified CSCs and their secreted EVs from A549 cells. In contrast, research on CSCs-EVs has been limited in previous studies, particularly in their application as drug carriers [79]. This study innovatively utilized CSCs-EVs to encapsulate TSRP and enhanced its targeting specificity and stability through TMTP1 modification. This approach not only strengthened TSRP’s anti-tumor effects but also provided new strategies and methods for cancer treatment.

To comprehensively validate the efficacy of TMTP1-TSRP-EVs, this study conducted extensive in vitro and in vivo experiments. Through experiments such as CCK-8 assays, Transwell assays, and TUNEL staining, we systematically evaluated the impact of TMTP1-TSRP-EVs on tumor cell proliferation, migration, and apoptosis. Furthermore, by establishing mouse metastatic tumor models and humanized mouse models, we further confirmed the anti-tumor effects of TMTP1-TSRP-EVs in vivo. Compared to previous studies, this research presented more detailed and robust experimental evidence, demonstrating the effectiveness and safety of TMTP1-TSRP-EVs in inhibiting bone metastasis in PI3K-mutant NSCLC [50, 80, 81].

In conclusion, based on the above results, we can preliminarily draw the following conclusions: PI3K mutations can promote bone metastasis of NSCLC and lead to primary immune checkpoint resistance. Prepared TMTP1-TSRP-EVs can reshape the TME by inhibiting the PI3K/Akt/mTOR pathway to prevent bone metastasis and primary immune checkpoint resistance in PI3K-mutant NSCLC. This study provides a novel therapeutic strategy to address primary immune checkpoint resistance in NSCLC that is challenging to overcome with conventional treatments. Our research contributes to elucidating the mechanisms of NSCLC bone metastasis progression and developing new therapeutic targets for NSCLC bone metastasis.

This study demonstrates significant effects in preventing bone metastasis and reversing immune checkpoint resistance in NSCLC with PI3K mutations using TMTP1-TSRP-EVs. The scientific importance is highlighted by unveiling the critical role of the PI3K/Akt/mTOR signaling pathway in PIK3CA-mutant NSCLC and providing a novel peptide, TSRP, that enhances CD8^+^ T cell infiltration and suppresses PD-L1 expression by downregulating this pathway, reshaping the tumor immune microenvironment. Additionally, this study innovatively utilizes TMTP1-modified CSCs-EVs as drug carriers, improving the targeting and stability of TSRP and offering a new strategy for cancer treatment. The clinical significance lies in the potential of this novel therapeutic approach to overcome the limitations of current treatments, particularly addressing resistance issues caused by PI3K mutations, providing a new treatment option that significantly improves the prognosis and survival rates of NSCLC patients.

M1 macrophages, often referred to as “classically activated” macrophages, produce pro-inflammatory cytokines such as TNF-α, IL-1β, and IL-6, as well as nitric oxide (NO), and exhibit strong anti-tumor activity. They enhance anti-tumor immune responses by promoting the activation and proliferation of CD8^+^ T cells [82]. In contrast, M2 macrophages, termed “alternatively activated” macrophages, are involved in tissue repair and anti-inflammatory responses, secreting cytokines like IL-10 and TGF-β to suppress inflammation. Within the tumor microenvironment, M2 macrophages often promote tumor growth and metastasis while inhibiting anti-tumor immune responses, possibly by enhancing angiogenesis and immunosuppression [83, 84]. TMTP1-TSRP-EVs effectively remodel the tumor microenvironment by increasing the infiltration of M1 macrophages and CD8^+^ T cells while reducing M2 macrophage infiltration, thereby enhancing anti-tumor immune responses and providing new therapeutic strategies.

Despite the important progress achieved in this study, certain limitations exist. Primarily, the research primarily conducted in vitro cell experiments and mouse models lacks clinical trial data, necessitating further validation of its safety and efficacy in humans. Secondly, limitations in sample size may affect the generalizability of results, particularly regarding their applicability in different genetic backgrounds and tumor types. Additionally, the idealized experimental conditions may not fully reflect the complexity of real clinical settings and individual variabilities that could impact treatment outcomes. Lastly, the lack of long-term observational and follow-up data necessitates further research into the long-term effects and potential side effects of TSRP.

Although this study provides preliminary evidence for the potential link between PIK3CA mutations and macrophage infiltration, further validation is necessary. Future studies will combine single-cell transcriptomics and functional validation experiments to explore how PIK3CA mutations directly or indirectly affect macrophage phenotypes and their roles within the tumor microenvironment at the molecular and cellular levels. Moreover, elucidating the immunomodulatory mechanisms of TSRP in the tumor microenvironment will uncover its therapeutic potential.

Future research should focus on optimizing the preparation process of TMTP1-TSRP-EVs to ensure their stability and efficacy in clinical applications. Further preclinical studies may involve expanding sample sizes to validate their suitability across different genetic backgrounds and tumor types, as well as exploring their long-term effectiveness and safety. Clinical trials may be a crucial next step, involving rigorous trial design and monitoring to evaluate the therapeutic effects of TMTP1-TSRP-EVs in humans. Additionally, it is essential to delve into the mechanism of action of TSRP and investigate its potential applications in other types of cancer. This study demonstrates that TMTP1-TSRP-EVs effectively inhibit the PI3K/Akt/mTOR pathway, reversing immune checkpoint resistance in PI3K-mutant NSCLC. This provides a theoretical basis for their combination with existing immune checkpoint inhibitors (e.g., PD-1/PD-L1 inhibitors) to enhance immunotherapy efficacy. Additionally, tumor stem cell-derived EVs used as drug carriers offer a novel delivery method, improving drug targeting, stability, and reducing degradation in vivo. Future clinical research can optimize administration routes, such as intravenous or localized delivery, to enhance patient compliance and treatment outcomes. Although TMTP1-TSRP-EVs are tumor-targeting and theoretically reduce off-target effects on normal cells, clinical trials are needed to evaluate their safety and tolerability, ensuring no new adverse effects arise during clinical applications. Ultimately, the hope is for this novel treatment strategy to be successfully applied in clinical settings, providing more personalized and precise treatment options and significantly improving the quality of life and cure rates for cancer patients.

## Supplementary information

Supplementary Materials

## Data Availability

All data can be provided as needed.
