# Supplementary material for: Therapeutic efficacy of TMTP1-modified EVs in overcoming bone metastasis and immune resistance in PIK3CA mutant NSCLC
Source: Cell Death Dis. 2025 May 6;16(1):367. doi: 10.1038/s41419-025-07685-y (PMC12055990; doi:10.1038/s41419-025-07685-y)
Supplement: Supplementary file 1 — Supplementary Materials [file 41419_2025_7685_MOESM1_ESM.docx]

**
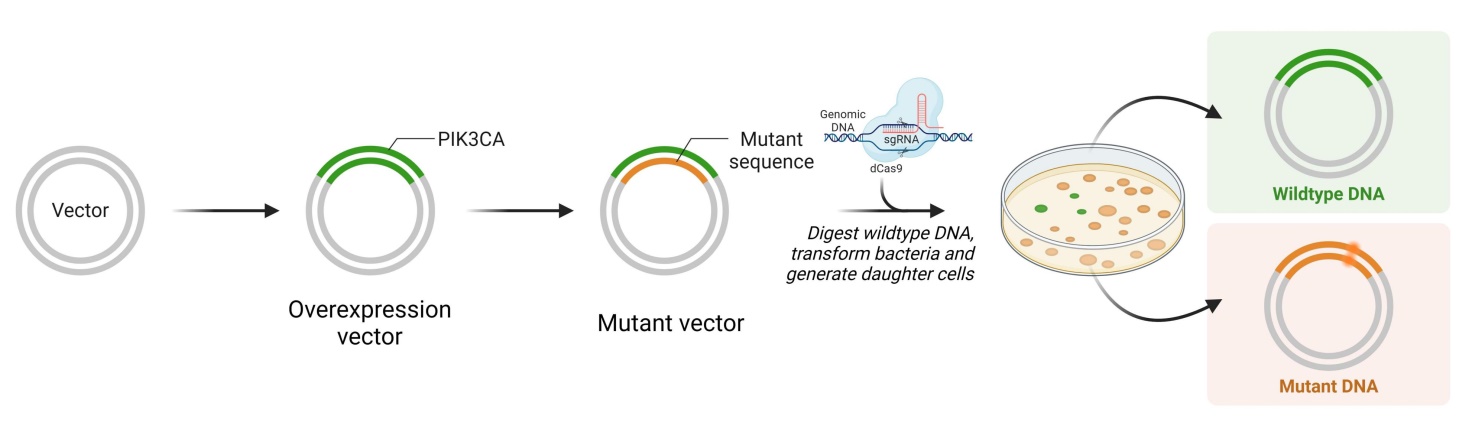
**

**Figure S1. Schematic of Constructing Mutant Overexpressing Cells.**

Note: Firstly, the overexpression vector of PIK3CA is constructed using CRISPR/Cas9 technology. Then, a PIK3CA-E545K site-directed mutation vector is created using the CRISPR/Cas9 editing system. After transfection into cells, wild-type and mutant cells are selected. (Created by BioRender.)

**
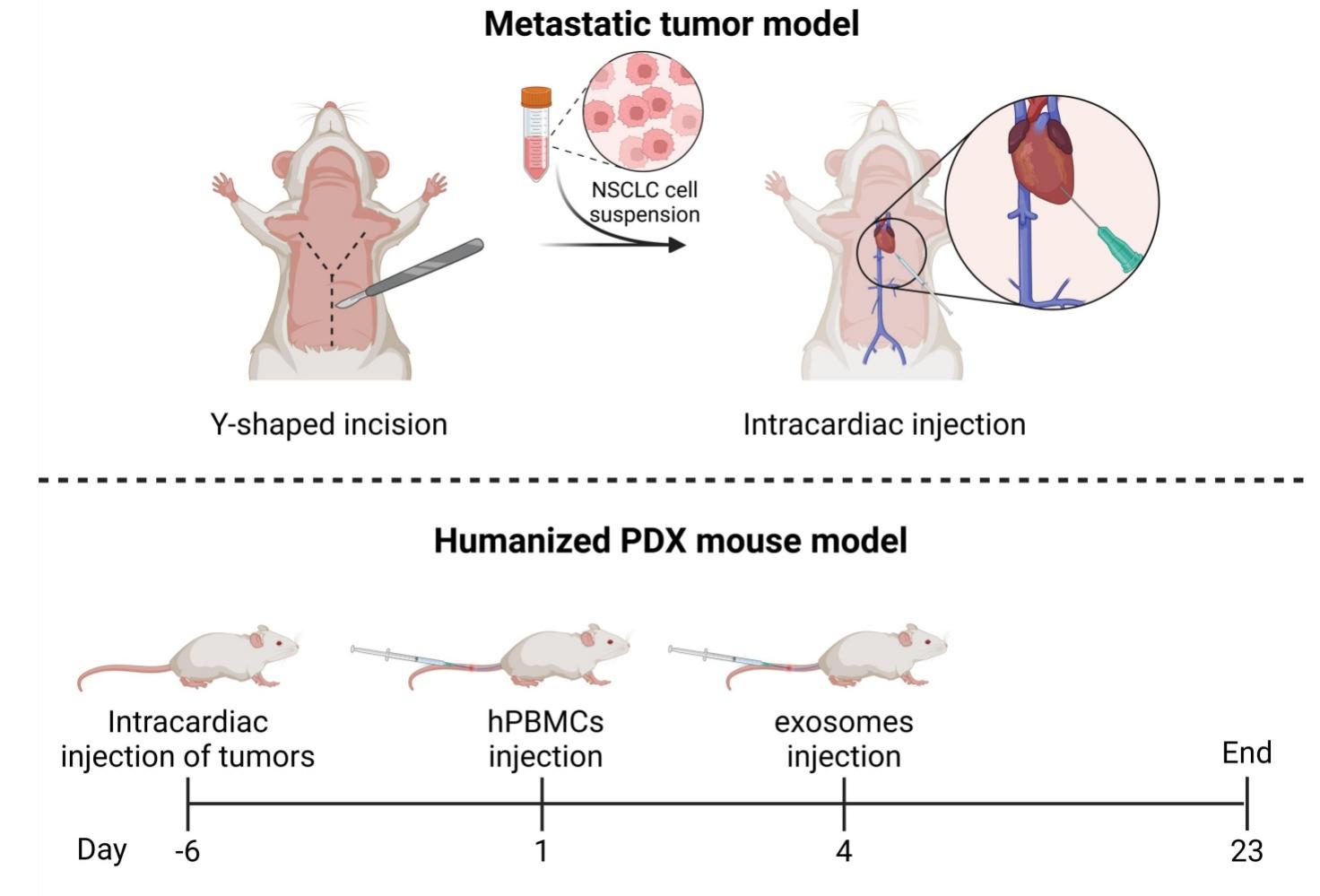
**

**Figure S2. Schematic of Establishing *In vivo* Animal Models for NSCLC.**

Note: The upper image depicts the schematic for constructing an NSCLC bone metastasis model, while the lower image shows the schematic for establishing a humanized mouse model. (Created by BioRender.)

**
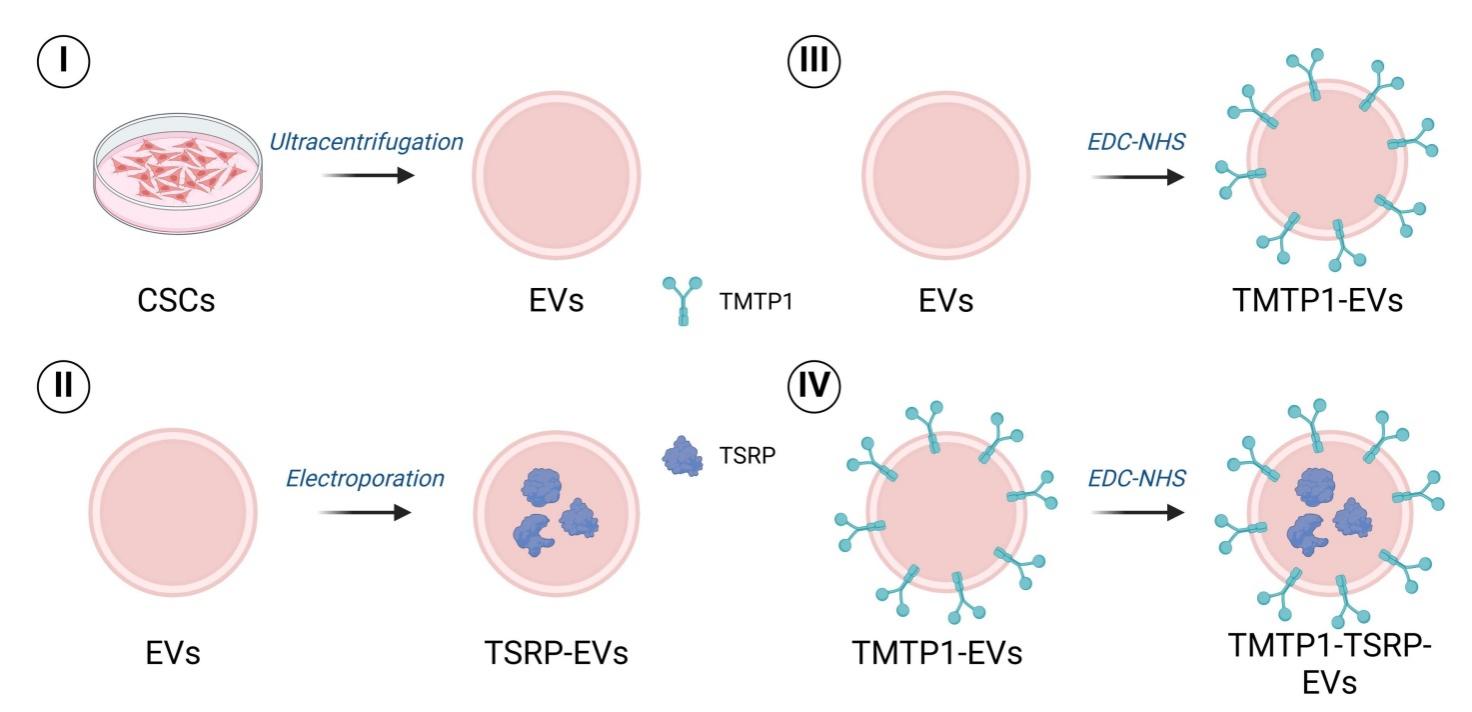
**

**Figure S3. Schematic Diagram of EVs Subgrouping.**

Note: Ⅰ Illustration of EVs isolated from CSCs; Ⅱ Schematic representation of TSRP-EVs obtained through electroporation of EVs; Ⅲ Schematic diagram of TMTP1-EVs obtained by coupling TMTP1 peptide using EDC-NHS method; Ⅳ Schematic representation of TMTP1-TSRP-EVs obtained by coupling TMTP1 peptide with TSRP-EVs. (Created by BioRender.)

**
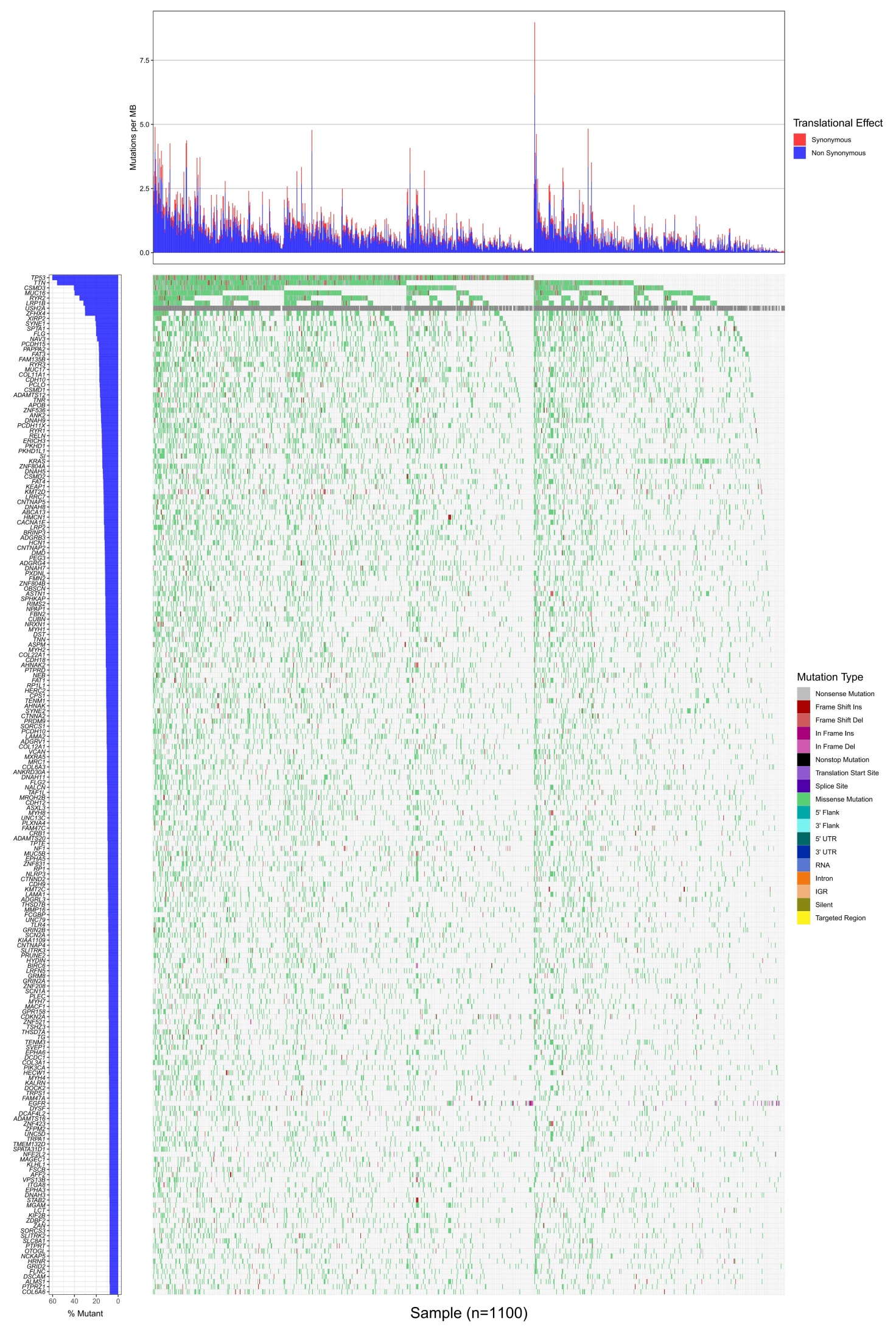
**

**Figure S4. Distribution of Frequently Mutated Genes in TCGA Database NSCLC.**

Note: Waterfall plots in the left panel display genes with high mutation frequencies in TCGA-LUAD and TCGA-LUSC samples, sorted by mutation frequency. The left side shows specific mutation frequencies of genes, while the right side lists different mutation types (N=1100).

**
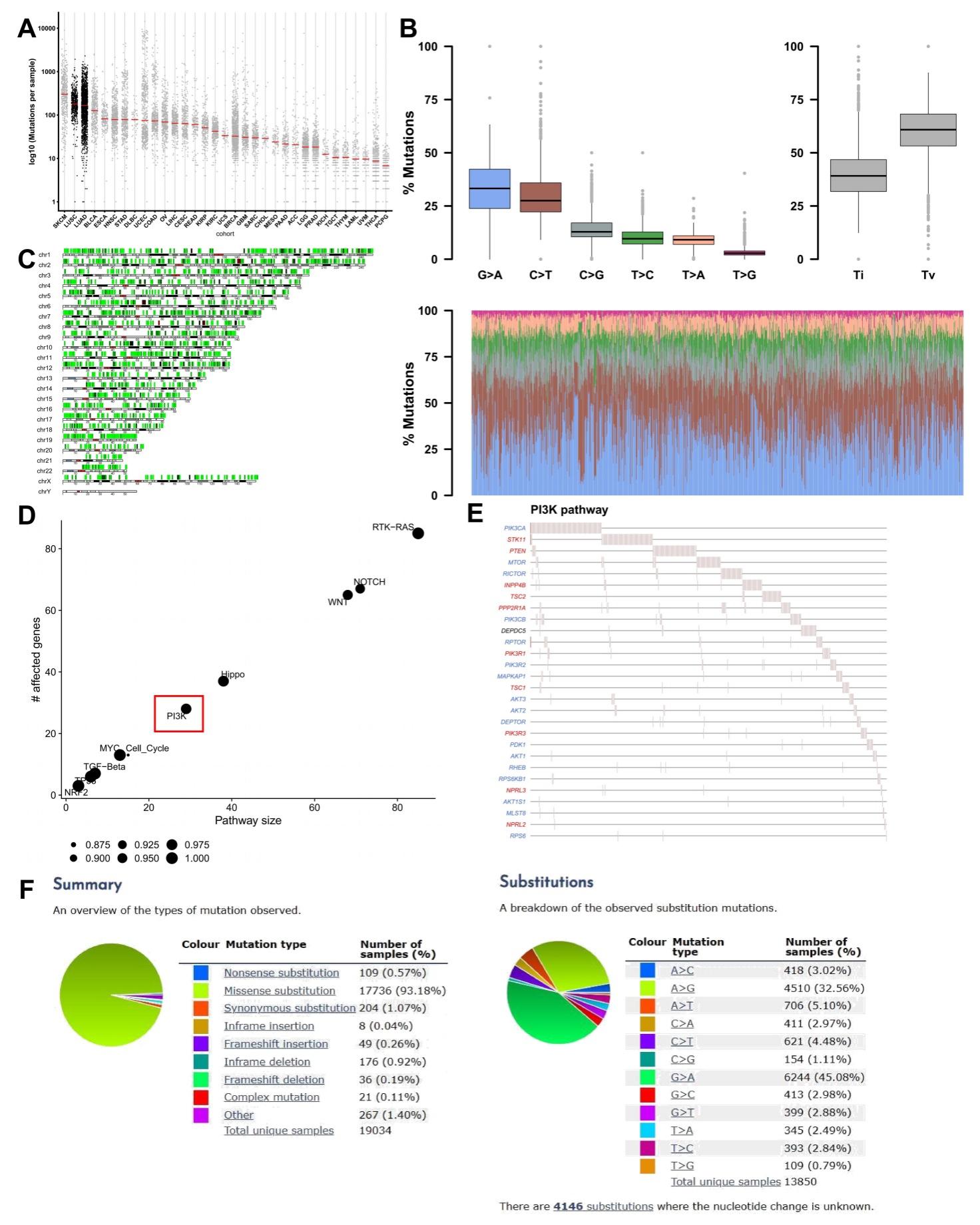
**

**Figure S5. Distribution of Frequently Mutated Genes in NSCLC from the TCGA database**

Note: (A) Comparison of mutation burdens in 33 types of cancers in TCGA, where the x-axis represents different tumor cohorts, the y-axis represents the number of somatic mutations per sample, with the red line indicating the median; (B) Overall representation of mutation types, nucleotide alteration rates, mutation spectra, and other mutation characteristics in TCGA-NSCLC samples (N=1100); (C) Distribution of mutated genes on chromosomes in ICGC-LUSC samples; (D) Display of significantly enriched pathways of mutated genes in TCGA-NSCLC samples; (E) Enrichment status of mutated genes in the PI3K pathway; (F) Summary of mutations in the PIK3CA gene in various cancers from the COSMIC database.

**
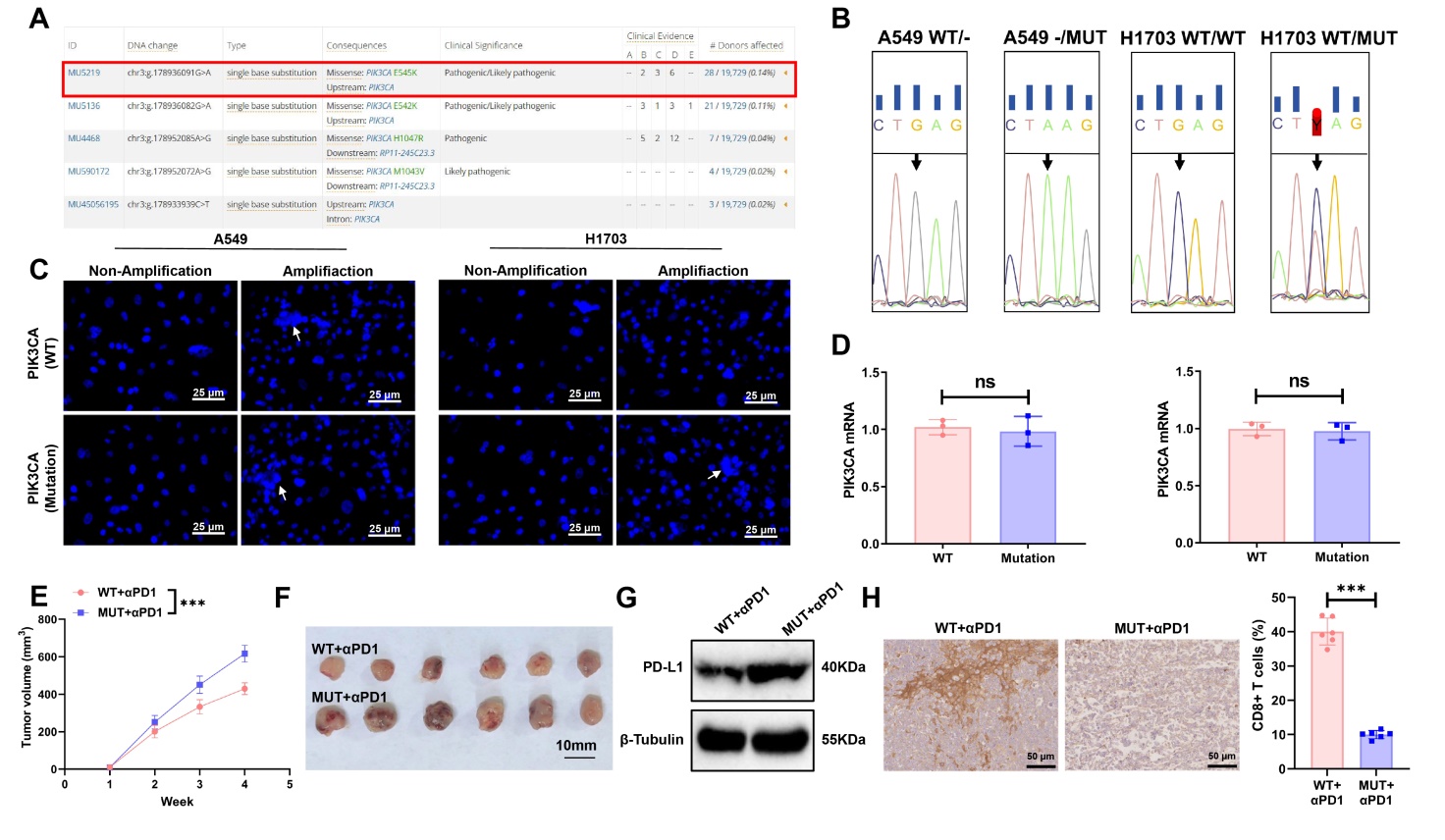
**

**Figure S6. PIK3CA mutation promotes primary immune checkpoint resistance in the H1703 xenograft model.**

Note: (A) Presentation of the results from the ICGC database on the distribution of PIK3CA mutation sites in NSCLC; (B) Confirmation of PIK3CA mutations in cell lines such as A549 and H1703 using Sanger sequencing, where "Y" indicates the presence of both C and T; (C) Detection results of PIK3CA amplification, with representative examples in the WT and Mutation groups shown (white arrows indicate NSCLC cells with PIK3CA amplification, scale bar=25 μm); (D) Measurement of PIK3CA gene mRNA levels in the WT and Mutation groups using RT-PCR; (E) Tumor size statistics of PIK3CA mutant NSCLC xenograft mice after Nivolumab treatment; (F) Morphology of tumors in PIK3CA mutant NSCLC xenograft mice after Nivolumab treatment; (G) Western blot analysis of PD-L1 protein expression levels in tumor tissues from PIK3CA-mutant NSCLC xenograft mice treated with Nivolumab; (H) IHC examination of CD8^+^ T cell infiltration levels in tumor tissues of PIK3CA mutant NSCLC xenograft mice after Nivolumab treatment, scale bar=50 μm. Cell experiments were repeated at least three times, with 6 mice in each group, "ns" indicates no significant difference, and *** represents *p* < 0.001.

**
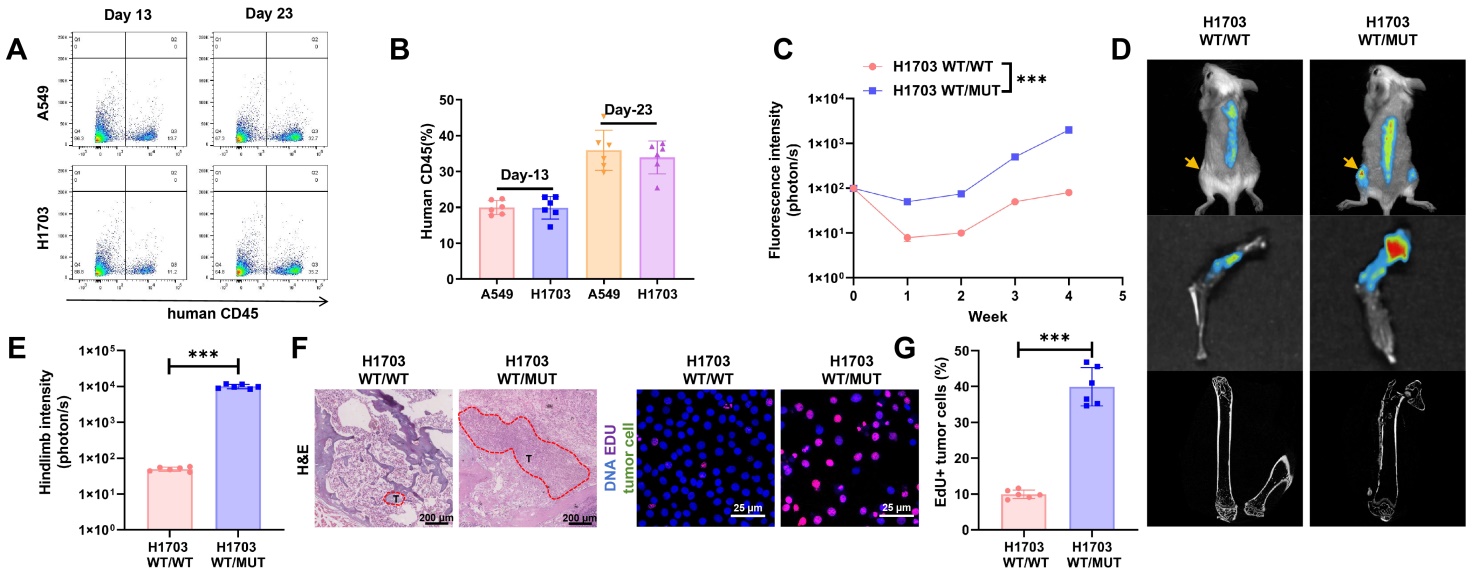
**

**Figure S7. PIK3CA Mutation Promotes Bone Metastasis in H1703 Xenograft Mice.**

Note: (A-B) Flow cytometry analysis to confirm the successful establishment of the humanized mouse model; (C) Fluorescence intensity results of NSCLC tumor tissues in different groups of mice; (D) Bioluminescence imaging results of NSCLC tumors and metastatic lesions in different anatomical regions of mice, with arrows indicating bone lesion areas; (E) Fluorescence intensity results of tumor tissues in the hind limbs where NSCLC tumors metastasized; (F) H&E staining of tumor tissue, scale bar=200 μm and EdU immunofluorescence imaging of tumor cells, scale bar=25 μm; (G) Statistical results of EdU experiments. Each group included 6 mice, and *** indicates *p* < 0.001.

**
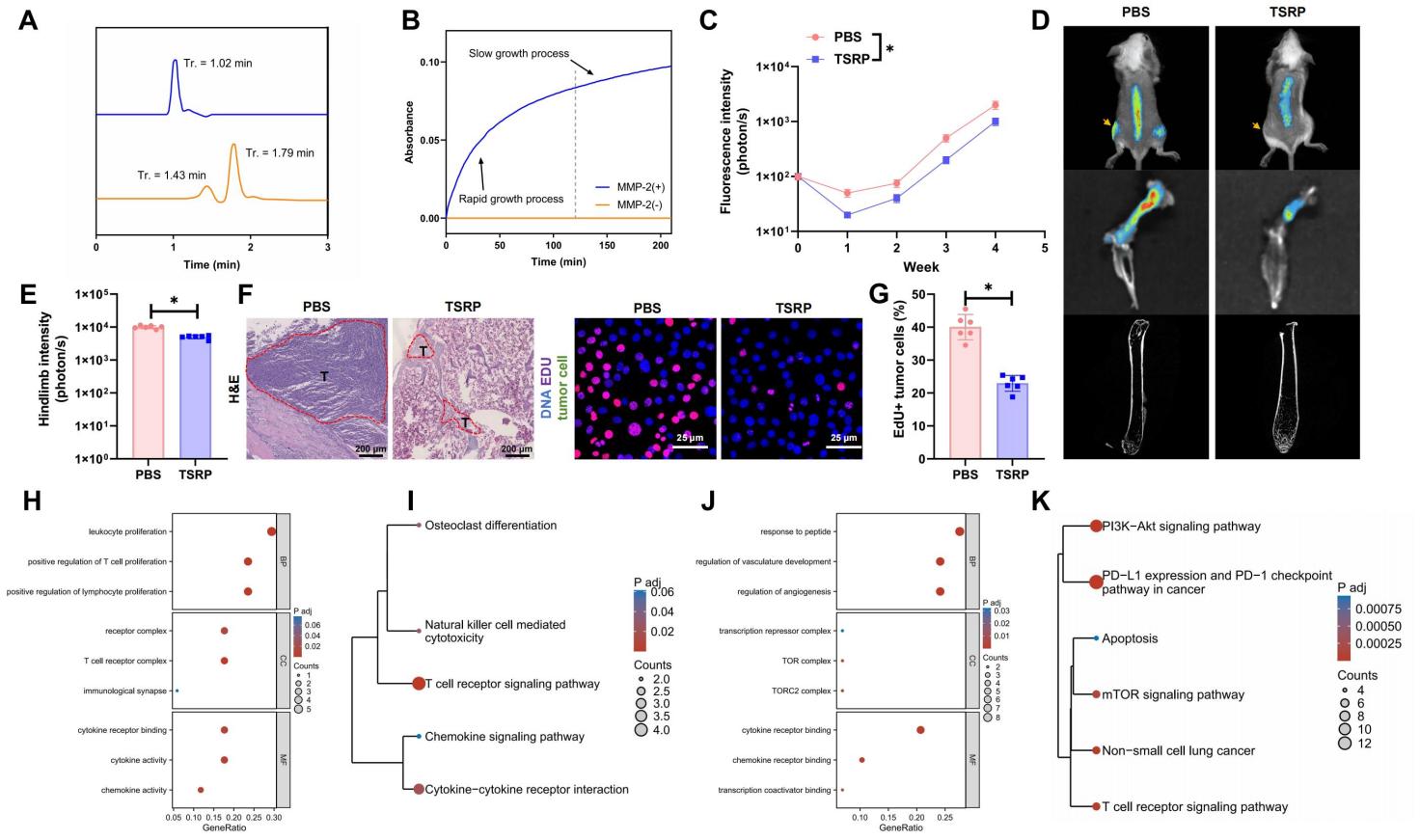
**

**Figure S8. TSRP inhibits bone metastasis in the H1703 xenograft model with PIK3CA mutations.**

Note: (A) HPLC and turbidity test results of synthesized TSRP; (B) Fluorescence intensity results of NSCLC tumor tissues in each group of mice; (C) Bioluminescence imaging results of NSCLC tumors and metastatic lesions in mice (arrows indicating bone injury area); (D) Fluorescence intensity results of NSCLC tumor metastasis to hind limb tumor tissue in each group of mice; (E) H&E staining of tumor tissue and EdU immunofluorescence imaging results of tumor cells, scale bar=200 μm; (F) Statistical results of EdU experiment; (G) Bubble diagram of GO enrichment for highly expressed genes in transcriptome sequencing (H) and KEGG clustering tree diagram (I); (J-K) Bubble diagram of GO enrichment for low expressed genes in transcriptome sequencing (J) and KEGG clustering tree diagram (K). BP represents Biological Process; CC represents Cell Component, and MF represents Molecular Function. 6 mice per group, * indicates *p* < 0.05.

**
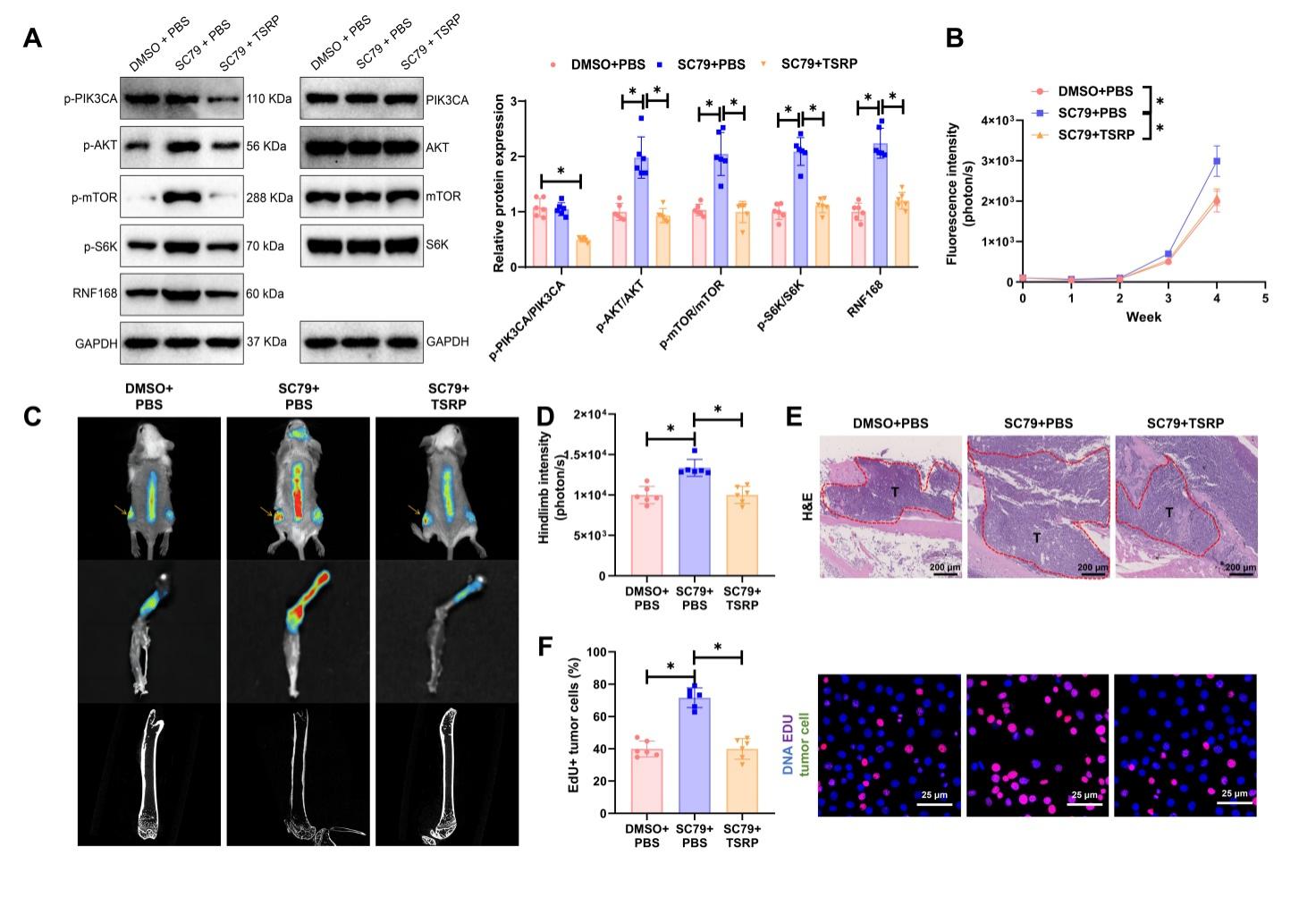
**

**Figure S9. TSRP suppresses the PI3K/Akt/mTOR pathway in the H1703 xenograft model with PIK3CA mutations.**

Note: (A) Detection of phosphorylation levels of PI3K/Akt/mTOR pathway proteins in tumor tissues metastasized to bone in mice; (B) Fluorescence intensity results of NSCLC tumor tissues in each group of mice; (C) Bioluminescence imaging results of NSCLC tumors and metastatic lesions in mice (arrows indicating bone injury area); (D) Fluorescence intensity results of NSCLC tumor metastasis to hind limb tumor tissue in each group of mice; (E) H&E staining of tumor tissue, scale bar=200 μm and EdU immunofluorescence imaging results of tumor cells, scale bar=25 μm; (F) Statistical results of EdU experiment. 6 mice per group, * indicates *p* < 0.05.

**
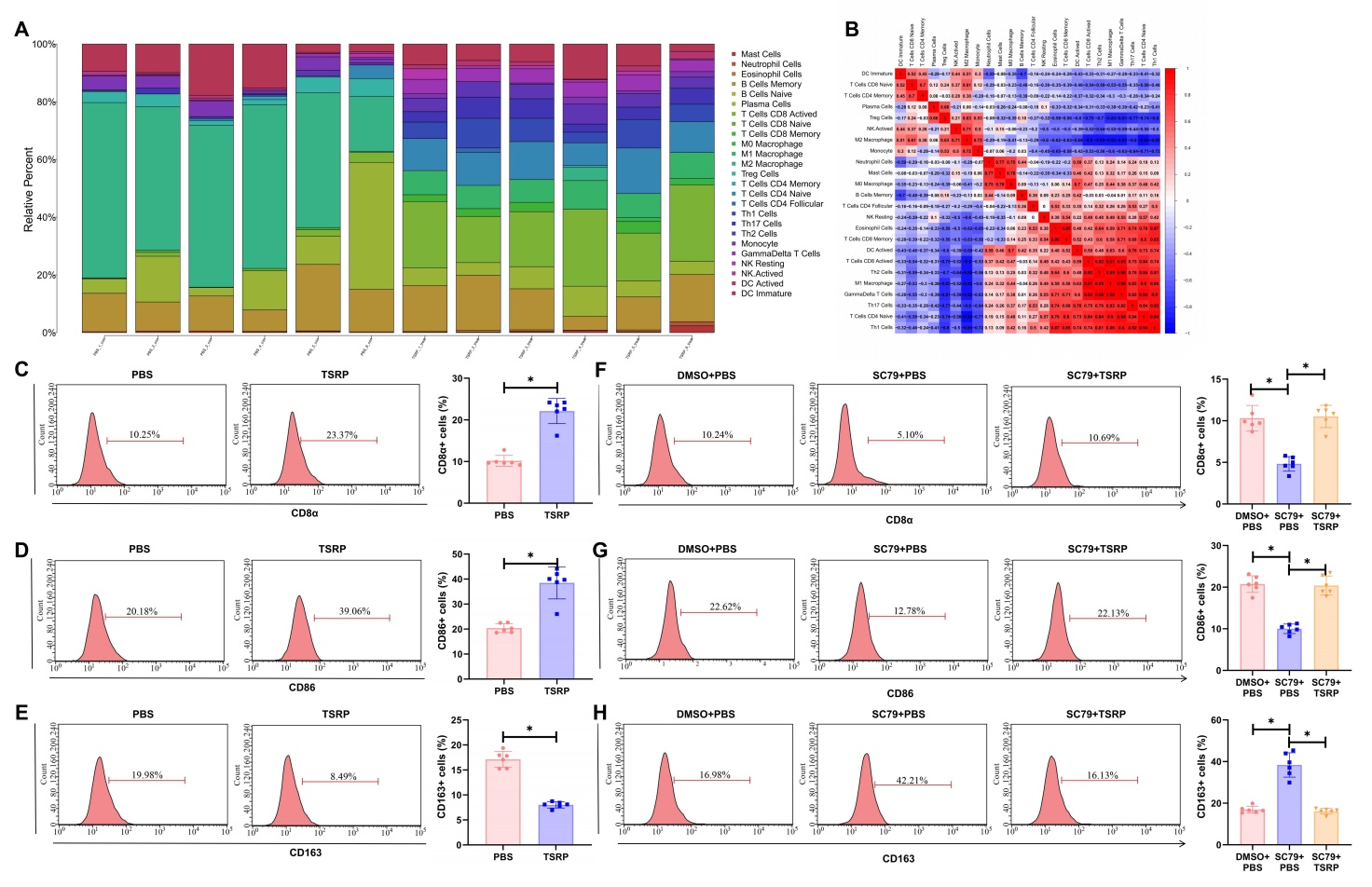
**

**Figure S10. TSRP inhibits the PI3K pathway and reshapes the tumor microenvironment in PI3K-mutant H1703 xenograft mice.**

Note: (A) Immune infiltration analysis results of tumor tissues metastasized to bone in mice from PBS group and TSRP group; (B) Schematic diagram illustrating the impact of TSRP on the NSCLC tumor immune microenvironment validated through experiments; (C-E) Flow cytometry analysis showing the effect of TSRP on the content of CD8^+^ T cells and macrophage subtypes in tumor tissues of each group; (F-H) Flow cytometry analysis demonstrating the impact of TSRP mediating the PI3K/Akt/mTOR pathway on the content of CD8^+^ T cells and macrophage subtypes in tumor tissues of each group. 6 mice per group, * indicates *p* < 0.05.

**
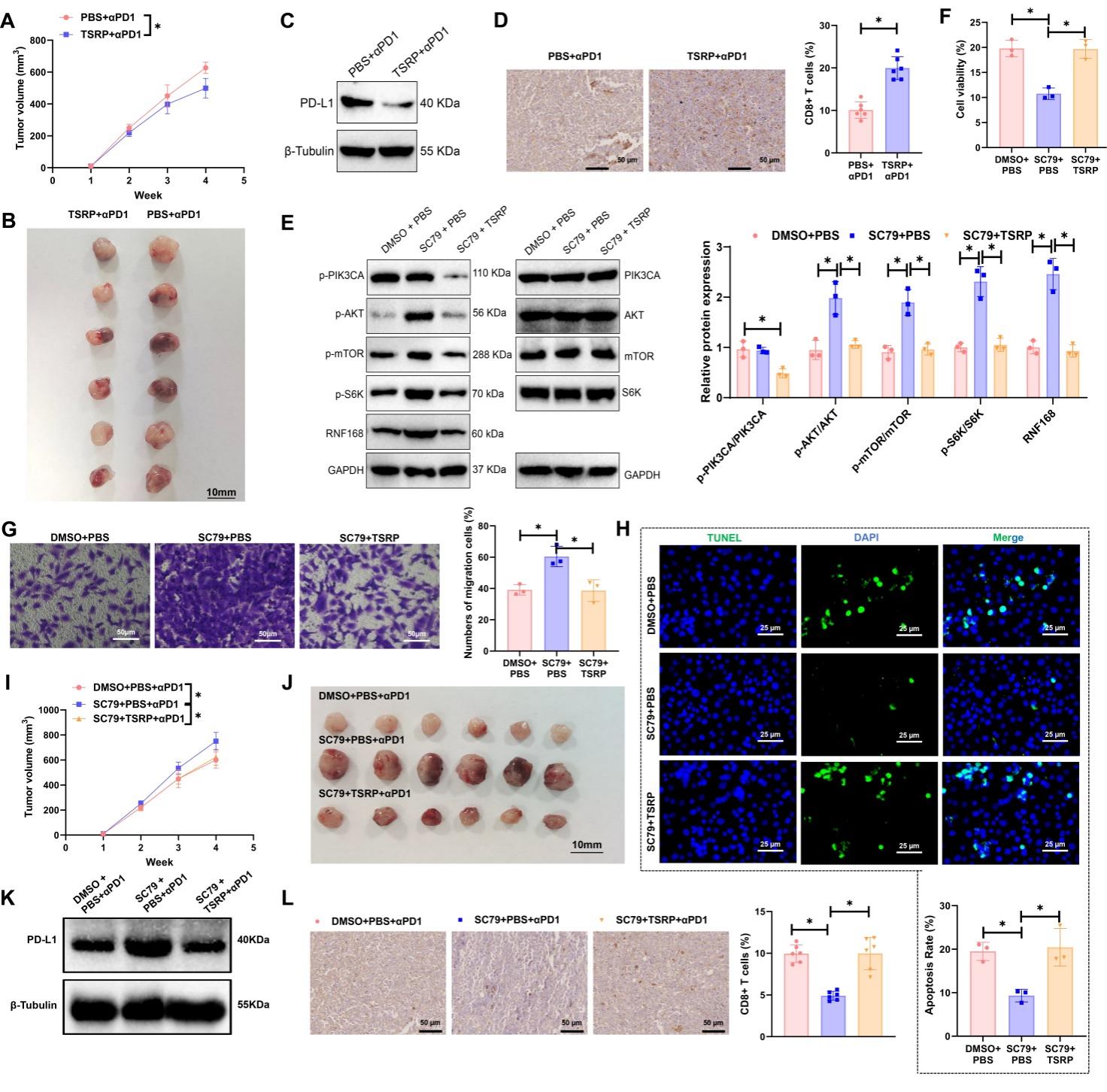
**

**Figure S11. TSRP inhibits the PI3K/Akt/mTOR pathway to regulate primary immune checkpoint resistance in PI3K-mutant H1703 xenograft models.**

Note: (A) Tumor size statistics of PIK3CA mutant NSCLC model mice in Nivolumab treatment groups; (B) Morphology of tumors in PIK3CA mutant NSCLC model mice after Nivolumab treatment; (C) Detection of PD-L1 protein expression levels in tumor tissues of PIK3CA mutant NSCLC model mice post Nivolumab treatment; (D) IHC detection of CD8^+^ T cell infiltration levels in tumor tissues of PIK3CA mutant NSCLC model mice post Nivolumab treatment, scale bar=50 μm; (E) Western blot analysis of protein phosphorylation levels in the PI3K/Akt/mTOR pathway in co-culture cell models from different groups; (F) Viability of CD8^+^ T cells in co-culture cell models; (G) Migration ability of tumor cells in co-culture cell models, scale bar=50 μm; (H) Apoptosis status of tumor cells in co-culture cell models, scale bar=25 μm; (I) Tumor size statistics of PIK3CA mutant NSCLC model mice post Nivolumab treatment; (J) Morphology of tumors in PIK3CA mutant NSCLC model mice after Nivolumab treatment; (K) Western blot analysis of PD-L1 protein expression levels in tumor tissues from PIK3CA-mutant NSCLC xenograft mice treated with Nivolumab; (L) IHC detection of CD8^+^ T cell infiltration levels in tumor tissues of PIK3CA mutant NSCLC model mice post Nivolumab treatment, scale bar=50 μm. Cell experiments were repeated at least three times, with 6 mice in each group, where * represents *p* < 0.05.

**
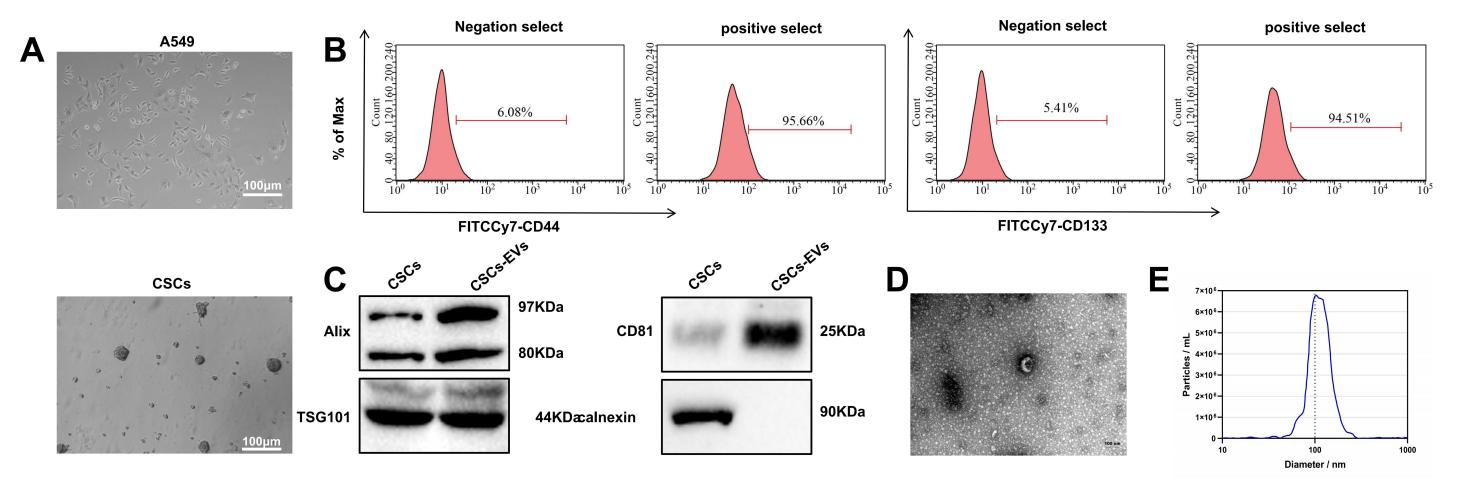
**

**Figure S12. Isolation and identification of CSCs-EVs through cell culture and characterization.**

Note: (A) Morphology of CSCs spheres derived from parental A549 cells and cultured differentiated cells, scale bar=100 μm; (B) Flow cytometry analysis of CSCs cell surface stem cell markers CD44 and CD133; (C) Protein expression levels of Alix, TSG101, and CD81 in EVs detected by Western blot; (D) Morphology of CSCs-EVs observed by TEM, with white arrows indicating the classic structure of cup-shaped vesicles (scale bar: 100 nm); (E) Size distribution analysis of CSCs-EVs through nanoparticle tracking. Cell experiments were repeated at least three times.

**
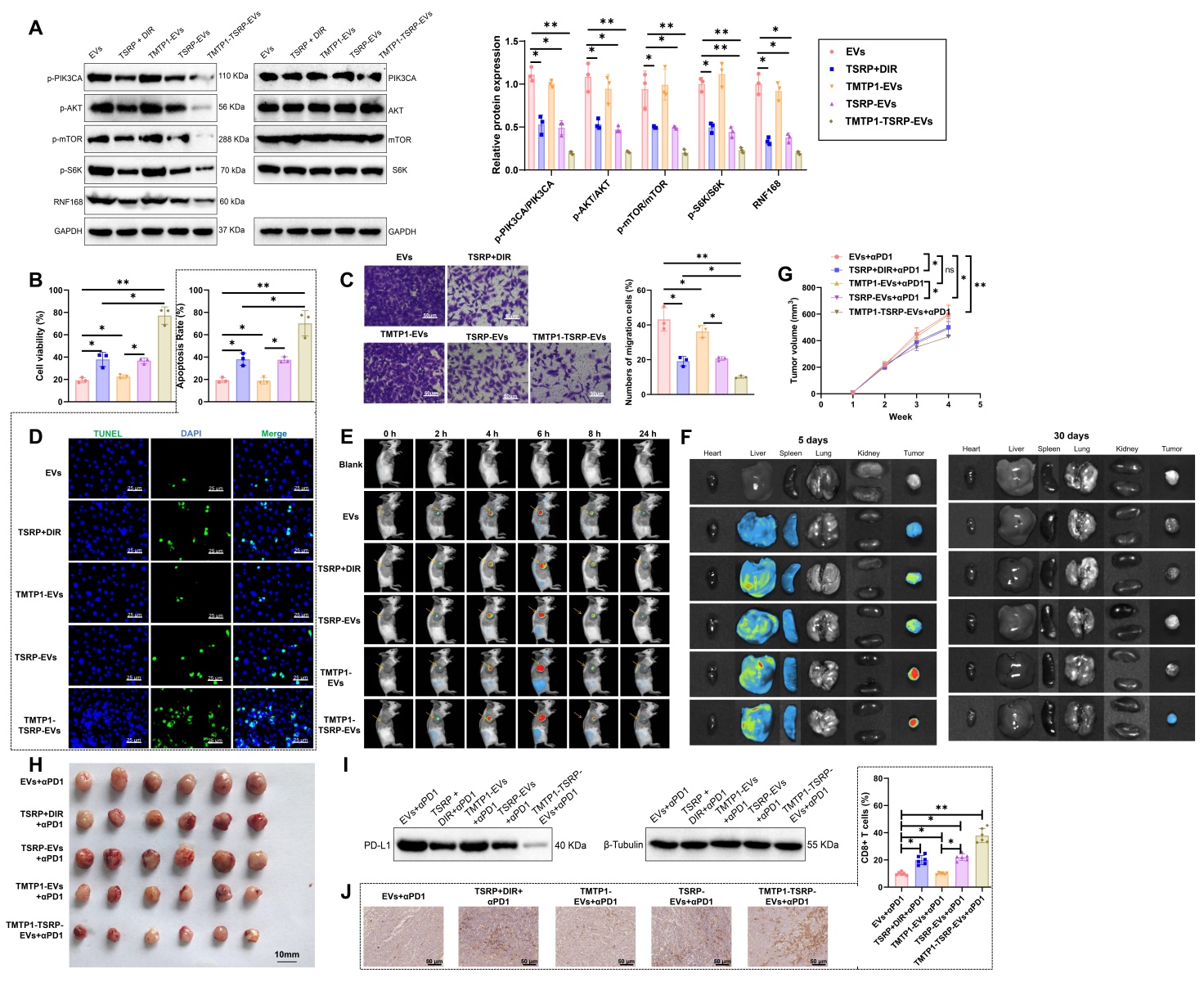
**

**Figure S13. TMTP1-TSRP-EVs reverse ICI resistance in PI3K-mutant H1703 xenograft mice.**

Note: (A) Western blot analysis was performed to detect the phosphorylation levels of proteins in the PI3K/Akt/mTOR pathway in co-culture cell models across different groups; (B) Assessment of viability of CD8^+^ T cells in co-culture cell models; (C) Evaluation of migratory capability of tumor cells in co-culture cell models, scale bar=50 μm; (D) Analysis of apoptosis status of tumor cells in co-culture cell models, scale bar=25 μm; (E) NIR fluorescence imaging to observe the *in vivo* fluorescence distribution of mice in each group; (F) NIR fluorescence imaging to observe the fluorescence distribution in major organs and tumor tissues of mice in each group; (G) Tumor size statistics of PIK3CA mutant NSCLC model mice post Nivolumab treatment in each group; (H) Morphology of tumors in PIK3CA mutant NSCLC model mice after Nivolumab treatment in each group; (I) Detection of PD-L1 protein expression levels in tumor tissues of PIK3CA mutant NSCLC model mice post Nivolumab treatment in each group; (J) IHC assessment of CD8^+^ T cell infiltration levels in tumor tissues of PIK3CA mutant NSCLC model mice post Nivolumab treatment, scale bar=50 μm. Cell experiments were repeated at least three times, with 6 mice in each group, where * denotes *p* < 0.05.

**
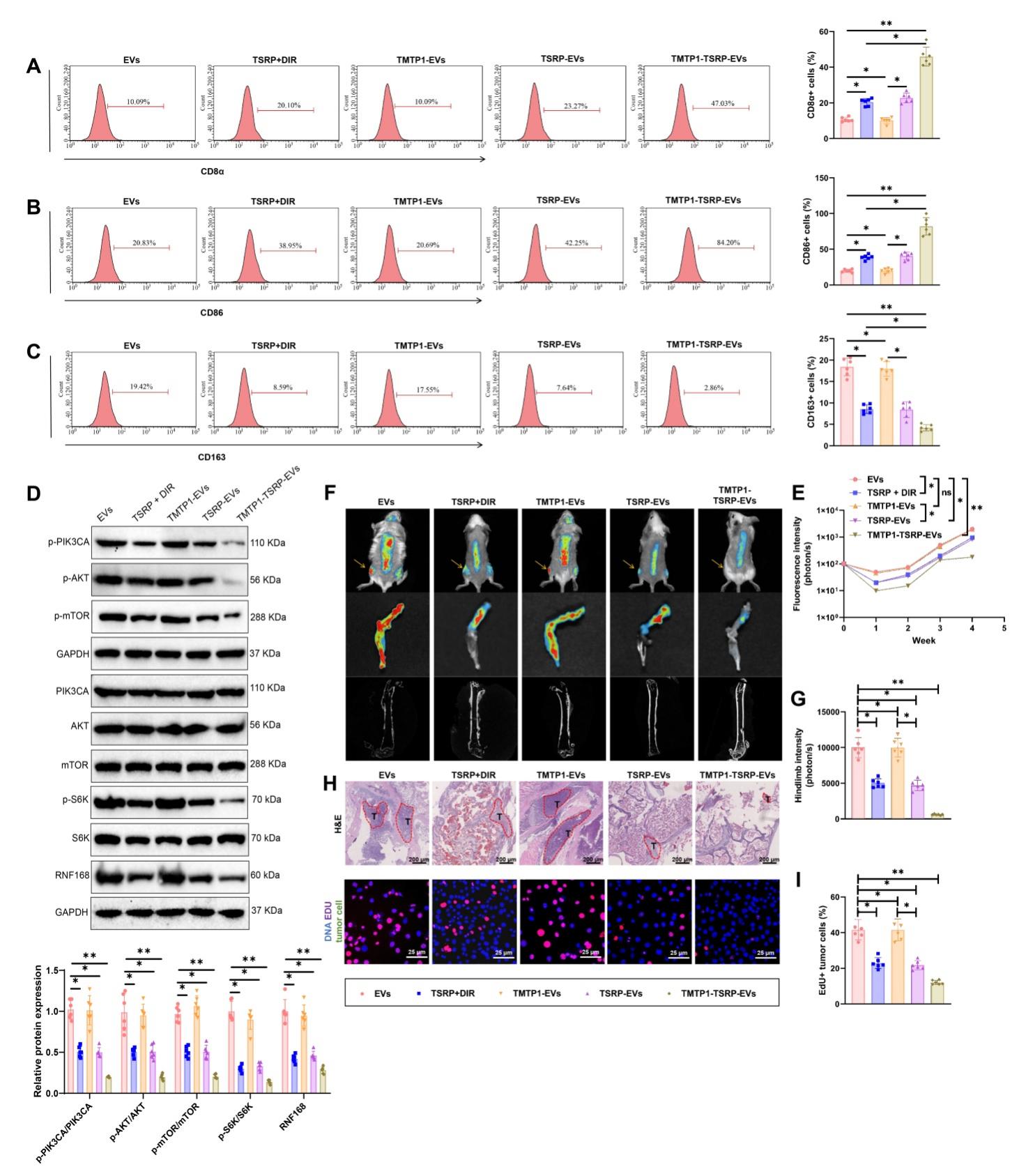
**

**Figure S14. TMTP1-TSRP-EVs remodel the tumor microenvironment and inhibit bone metastasis of PI3K-mutant H1703 cells *in vivo*.**

Note: (A-C) Analysis of the effect of TSRP on the content of CD8^+^ T cells and macrophage subtypes in tumor tissues of each group via flow cytometry; (D) Western blot analysis of phosphorylation levels of proteins in the PI3K/Akt/mTOR pathway in tumor tissues metastasized to bone from different groups of mice; (E) Fluorescence intensity in NSCLC tumor tissues of mice in each group; (F) Bioluminescent imaging results of NSCLC tumors and metastatic lesions in mice (arrows indicate areas of bone damage); (G) Fluorescence intensity in tumor tissues of hind limb metastases in NSCLC tumors in mice from each group; (H) H&E staining of tumor tissue, scale bar=200 μm and EdU immunofluorescence imaging of tumor cells, scale bar=25 μm; (I) Statistical results of EdU experiment. **p* < 0.05, ***p* < 0.01, with 6 mice in each group.

**
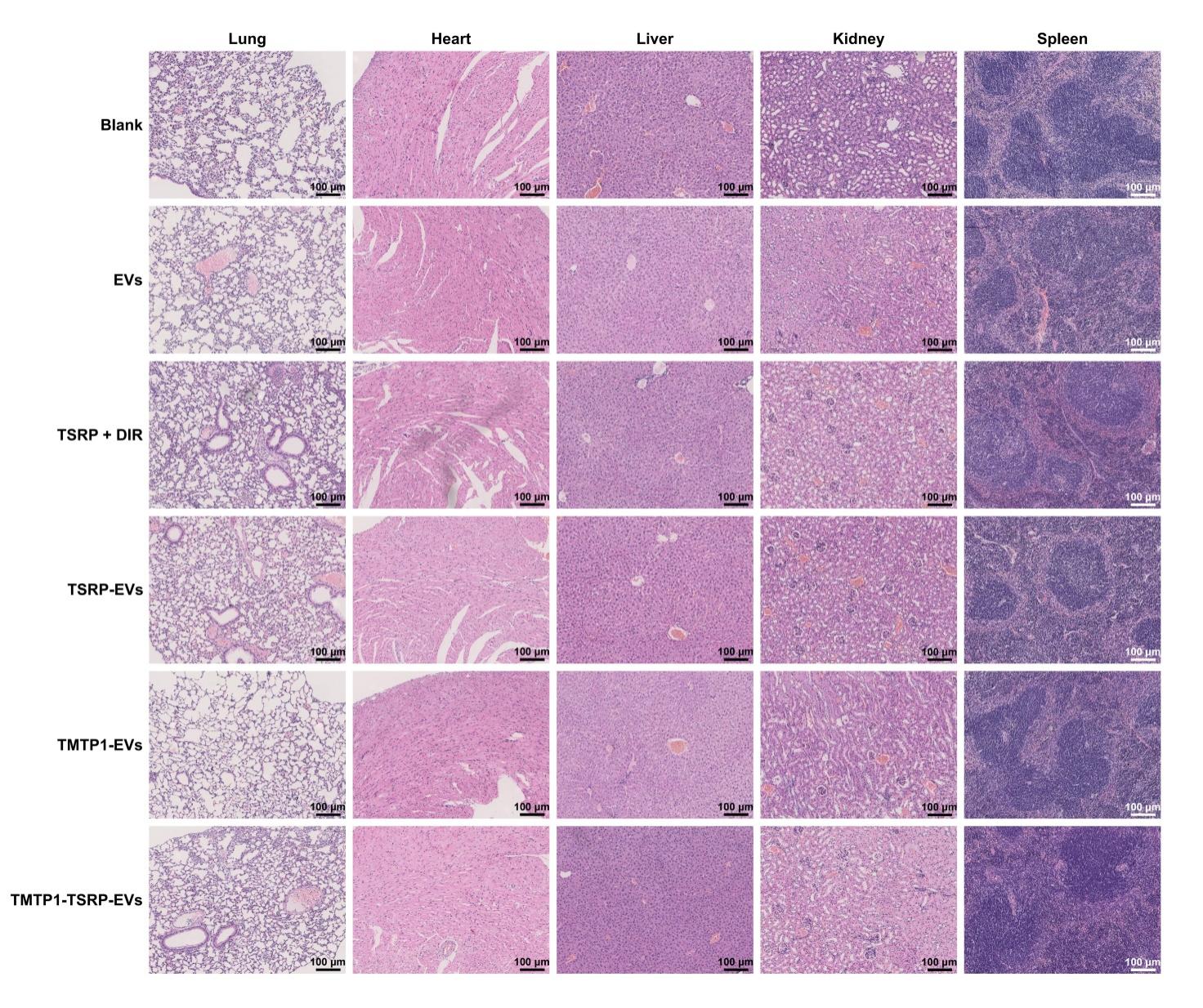
**

**Figure S15. H&E staining of major organs from PI3K-mutant A549 xenograft mice.**

Note: H&E staining images of lung, heart, liver, kidney, and spleen sections from mice in each group are shown in the figure, scale bar: 100 μm, with 6 mice in each group.

**Table S1. Details of IHC antibody product.**

| **Name** | **Host** | **Target** | **Cat.** | **DiIution Ratio** | **Manufacturer** | **Country** |
| --- | --- | --- | --- | --- | --- | --- |
| IgG | Goat | Rabbit | A-11008 | 1: 100 | Invitrogen | USA |
| IgG | Goat | Mouse | A-11005 | 1: 100 | Invitrogen | USA |
| PD-L1 | Mouse | Human | 14-5983-82 | 1: 100 | Invitrogen | USA |
| CD86 | Mouse | Human | PA5-114995 | 1: 200 | Invitrogen | USA |
| CD163 | Mouse | Human | PA5-78961 | 1: 200 | Invitrogen | USA |
| CD8α | Rabbit | Human | MA5-14548 | 1: 100 | Invitrogen | USA |
| Ki67 | Rabbit | Human | MA5-14520 | 1: 100 | Invitrogen | USA |

**Table S2. Details of flow cytometry antibody product.**

| **Name** | **Host** | **Target** | **Cat.** | **DiIution Ratio (µg/test)** | **Manufacturer** | **Country** | **Conjugation** |
| --- | --- | --- | --- | --- | --- | --- | --- |
| CD86 | Mouse | Human | 17-0869-42 | 0.25 | Invitrogen | USA | APC |
| CD163 | Mouse | Human | MA5-17719 | 0.25 | Invitrogen | USA | FITC |
| CD8α | Mouse | Human | 12-0088-42 | 0.125 | Invitrogen | USA | PE |
| CD44 | Rabbit | Human | 11-0441-82 | 0.5 | Invitrogen | USA | FITC |
| CD45 | Mouse | Human | 58-0459-42 | 0.06 | Invitrogen | USA | Alexa Fluor™ 532 |
| CD133 | Mouse | Human | 12-1338-42 | 0.5 | Invitrogen | USA | PE |
| EpCAM | Mouse | Human | 50-9326-42 | 0.06 | Invitrogen | USA | eFluor™ 660 |
| FITC | Goat | Rabbit | F-2765 | 1:500 | Invitrogen | USA | / |
| FITC | Goat | Mouse | F-2761 | 1-10 µg/mL | Invitrogen | USA | / |

**Table S3. RT-qPCR primer sequences.**

| **Gene** | **Primer Sequence** |
| --- | --- |
| GAPDH (human) | F: 5'-CGGATTTGGTCGTATTGGGC-3' |
|  | R: 5'-TTGACGGTGCCATGGAATTTG-3' |
| PIK3CA (human) | F: 5'-ACCCGATGCGGTTAGAGC-3' |
|  | R: 5'-CACCTGATGATGGTCGTGGA-3' |

Note: F: Forward; R: Reverse.

**Table S4. Details of the first antibody product.**

| **Name** | **Cat.** | **Dilution Ratio** | **Manufacturer** | **Country** | **MW (kDa)** |
| --- | --- | --- | --- | --- | --- |
| GAPDH | ab9485 | 1: 2500 | Abcam | UK | 37 |
| β-Tubulin | PA5-16863 | 1: 1000 | Invitrogen | USA | 55 |
| PIK3CA | MA5-14870 | 1: 1000 | Invitrogen | USA | 110 |
| AKT | MA5-37855 | 1: 1000 | Invitrogen | USA | 56 |
| S6K | PA5-28597 | 1: 1000 | Invitrogen | USA | 70 |
| mTOR | PA5-34663 | 1: 1000 | Invitrogen | USA | 288 |
| RNF168 | PA5-78642 | 1: 1000 | Invitrogen | USA | 60 |
| p-PIK3CA | PA5-105113 | 1: 1000 | Invitrogen | USA | 110 |
| p-AKT | PA5-36780 | 1: 1000 | Invitrogen | USA | 56 |
| p-Mtor | 44-1125G | 1: 1000 | Invitrogen | USA | 288 |
| p-S6K | 44-918G | 1: 1000 | Invitrogen | USA | 70 |
| PD-L1 | 14-5983-82 | 1: 100 | Invitrogen | USA | 40 |
| FITC | PA1-26793 | 1: 1000 | Invitrogen | USA | / |

Full and uncropped western blots


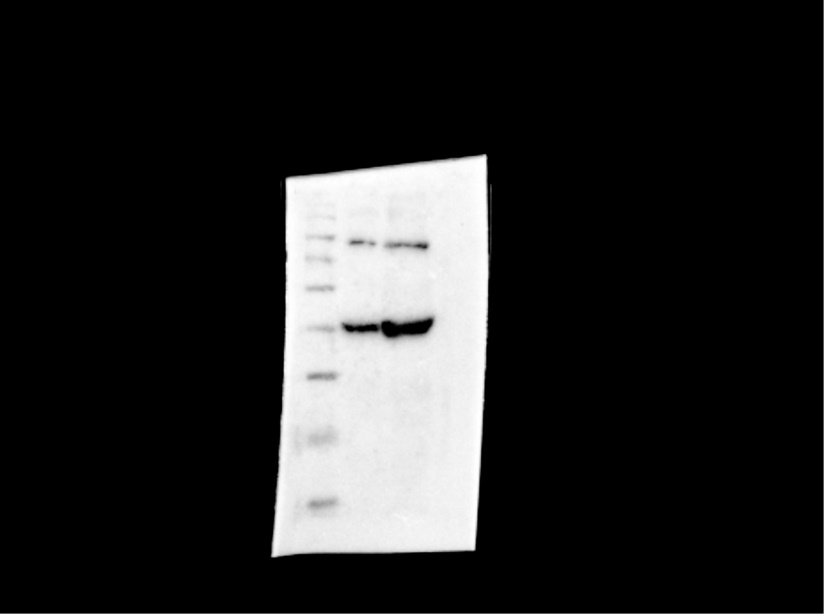


Full and uncropped western blots for Figure 2I-1


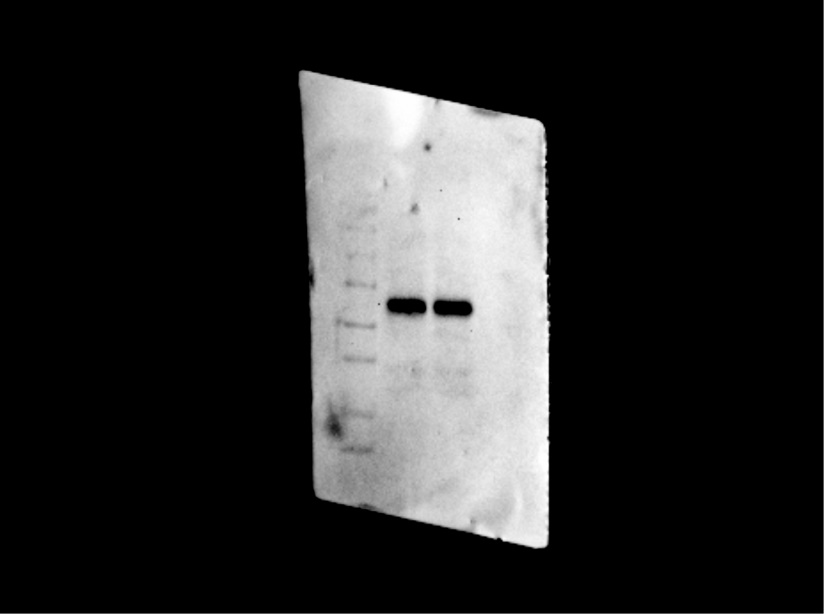


Full and uncropped western blots for Figure 2I-2


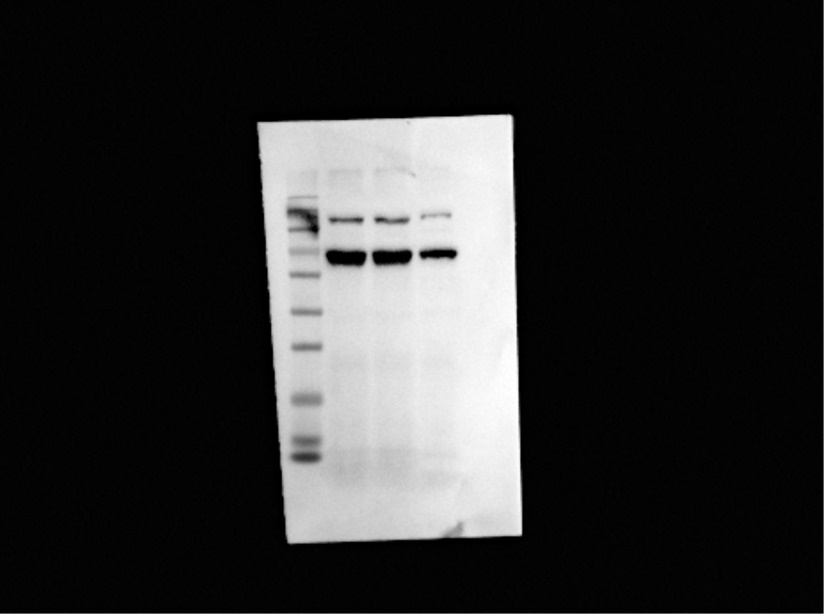


Full and uncropped western blots for Figure 3H-1 (1)


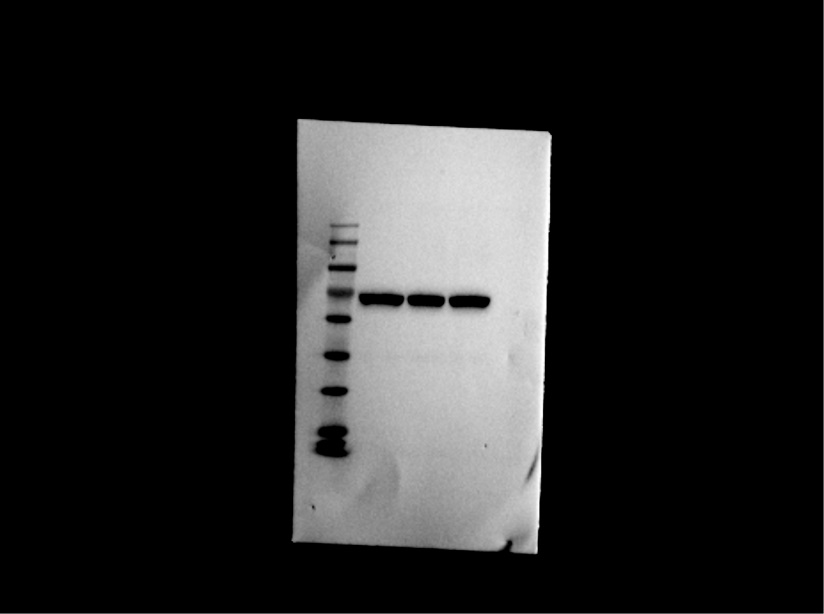


Full and uncropped western blots for Figure 3H-1 (2)


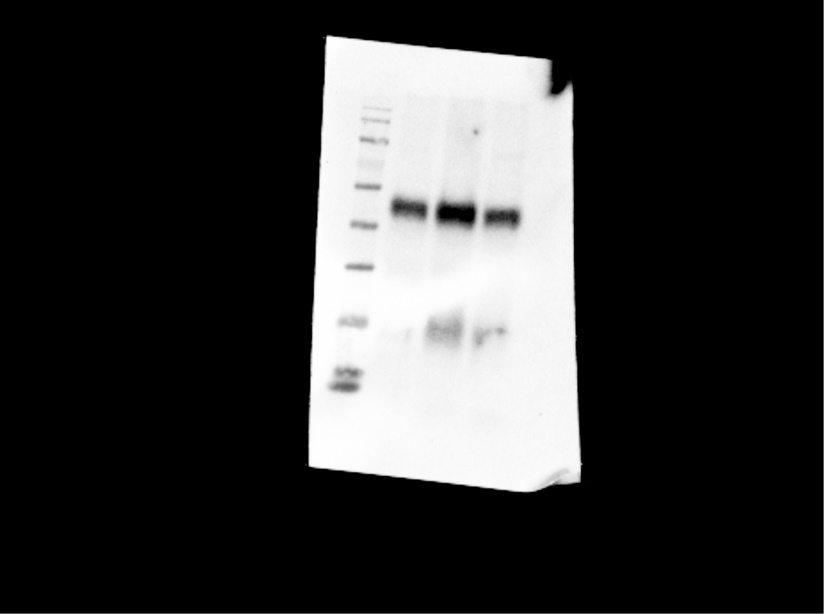


Full and uncropped western blots for Figure 3H-2 (1)


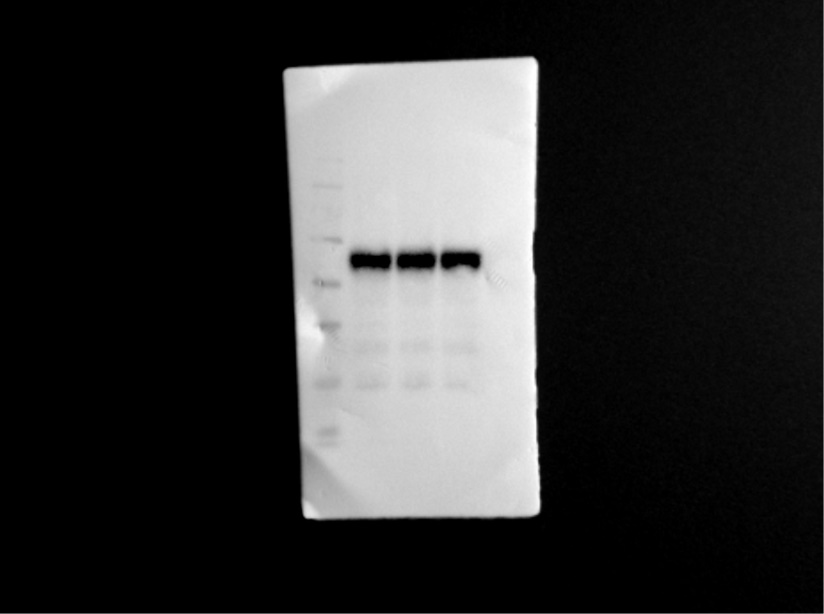


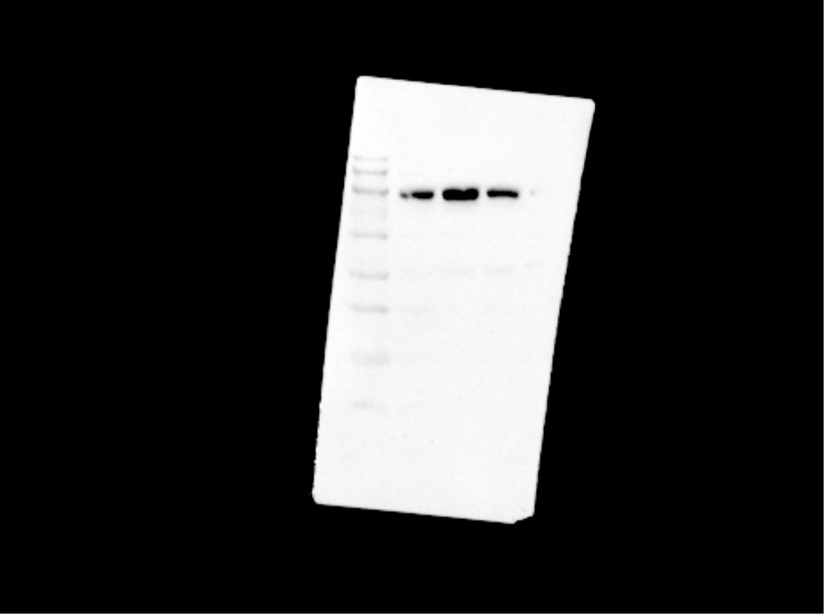


Full and uncropped western blots for Figure 3H-3 (1)


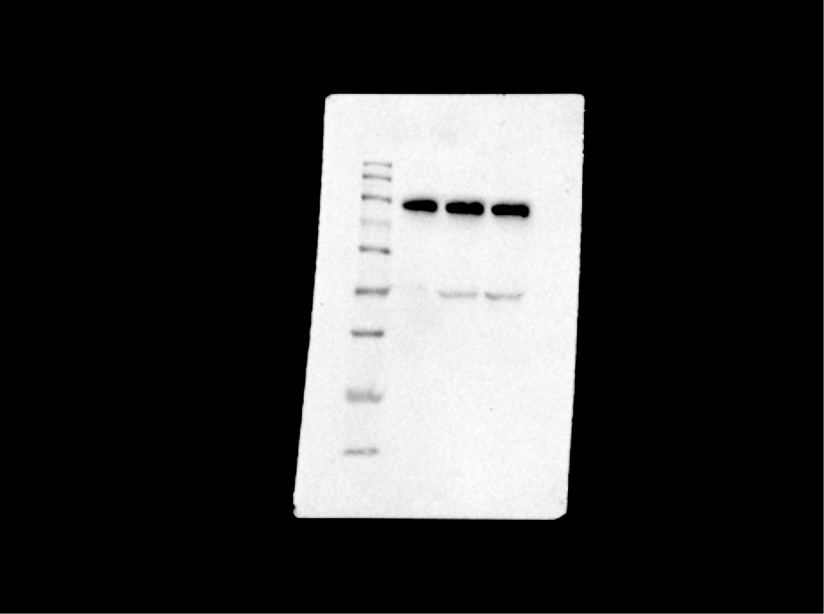


Full and uncropped western blots for Figure 3H-3 (2)


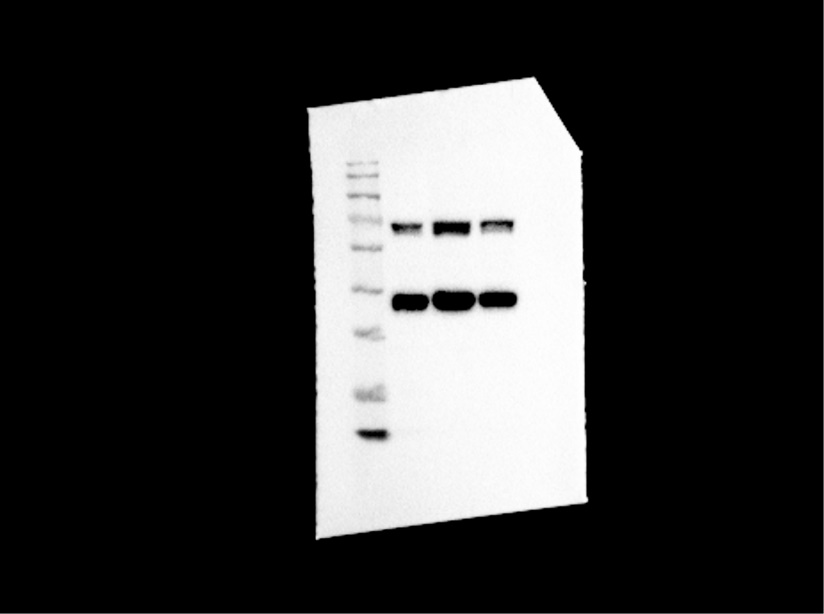


Full and uncropped western blots for Figure 3H-4-1


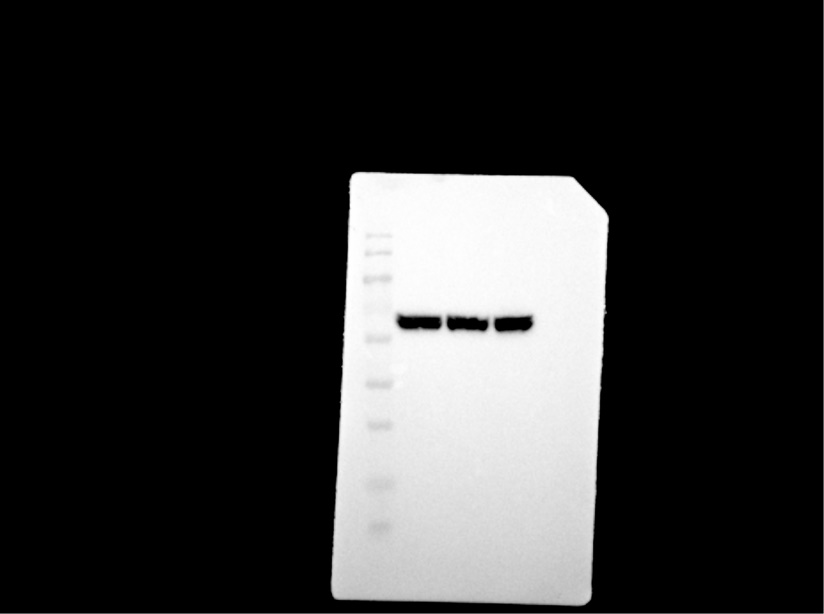


Full and uncropped western blots for Figure 3H-4-2


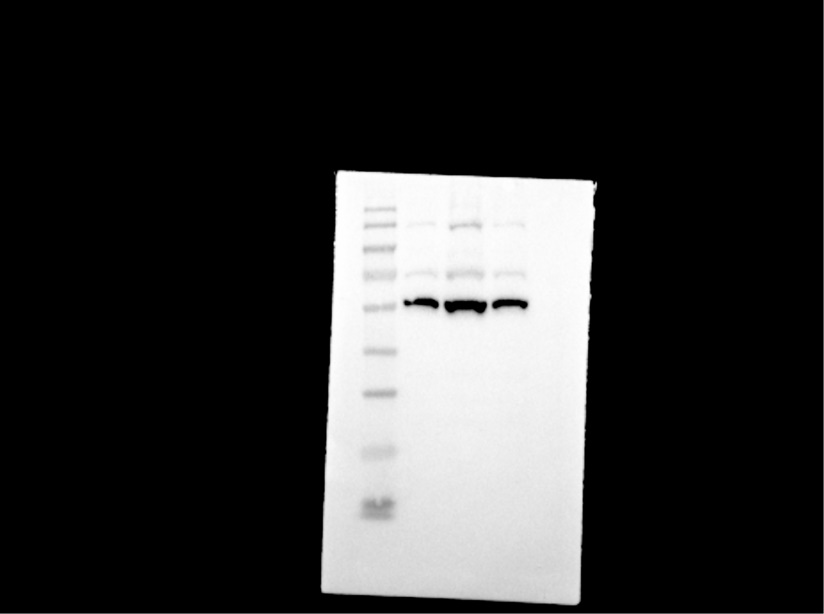


Full and uncropped western blots for Figure 3H-5


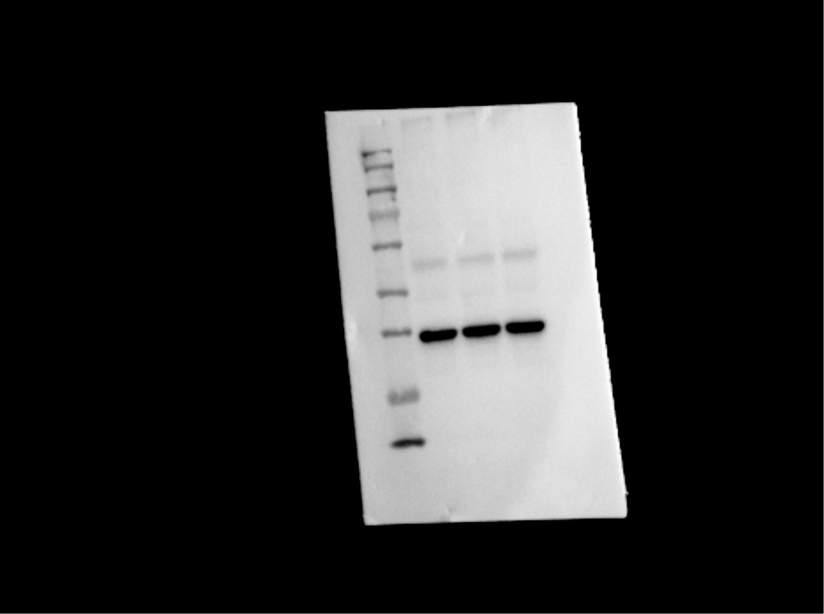


Full and uncropped western blots for Figure 3H-6 (1)


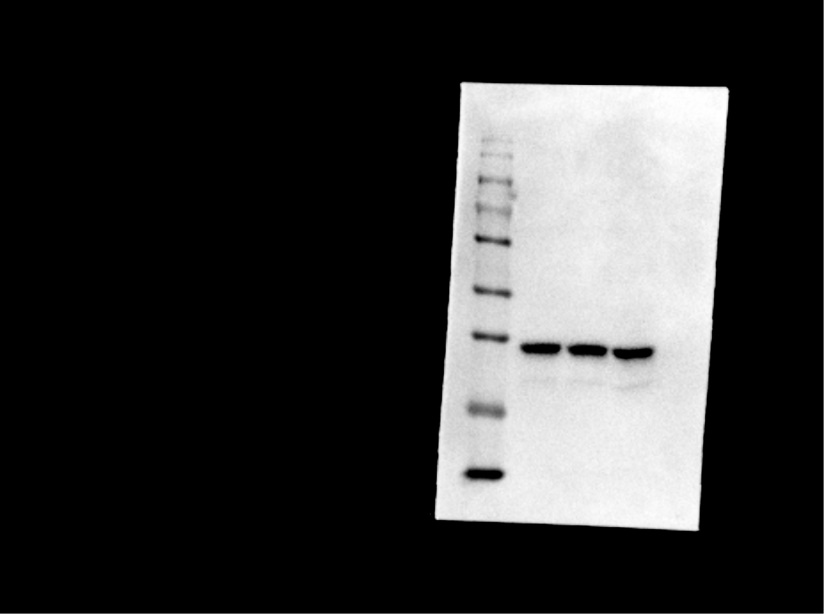


Full and uncropped western blots for Figure 3H-6 (2)


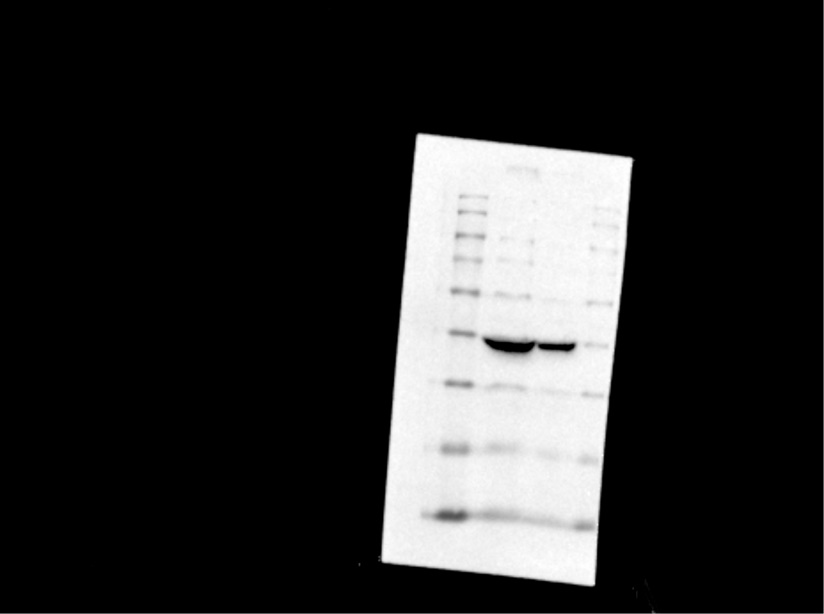


Full and uncropped western blots for Figure 5C-1


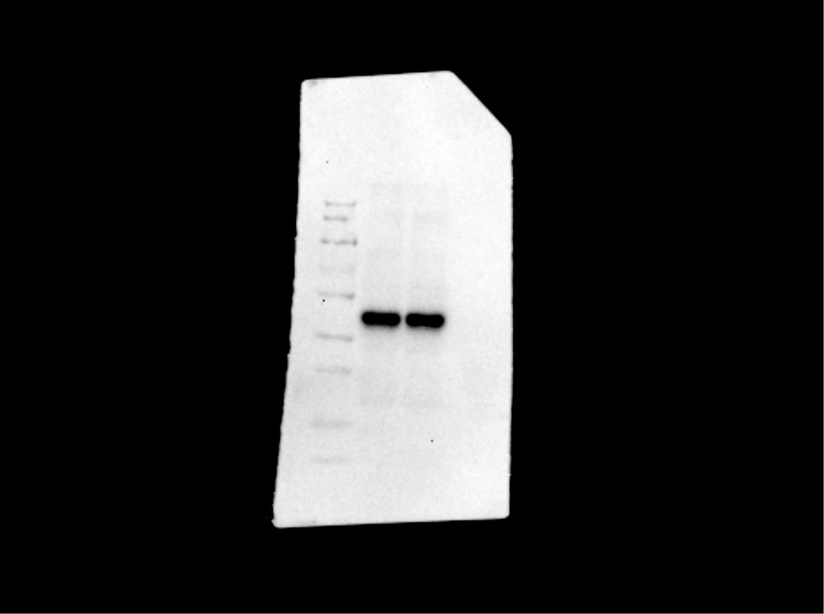


Full and uncropped western blots for Figure 5C-2


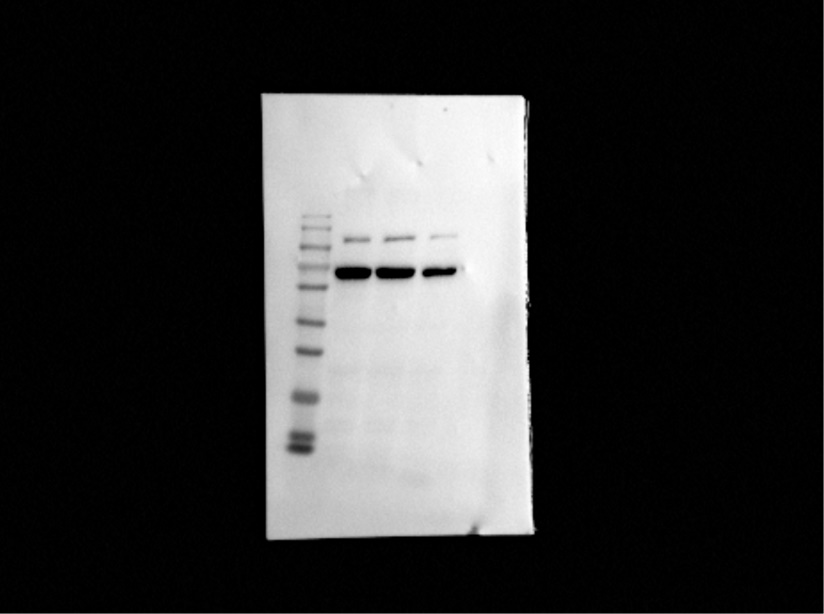


Full and uncropped western blots for Figure 5G-1 (1)


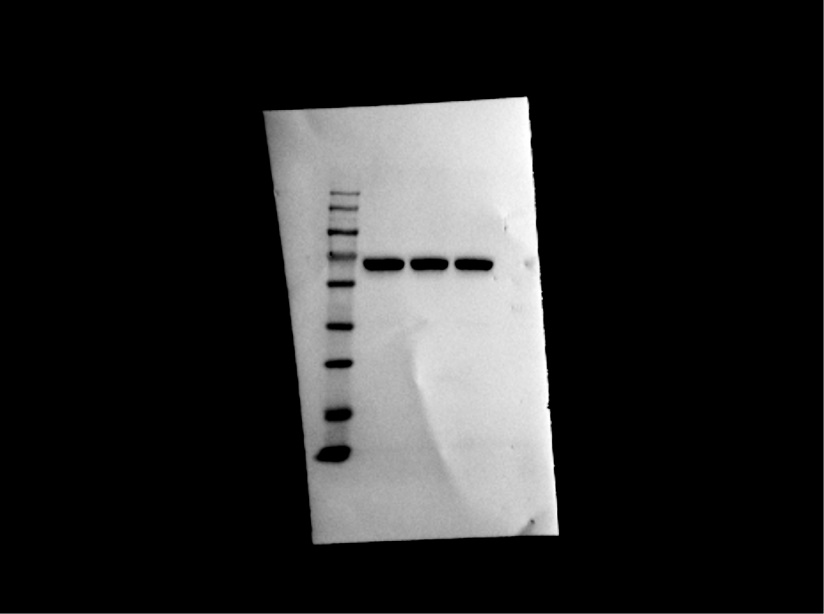


Full and uncropped western blots for Figure 5G-1 (2)


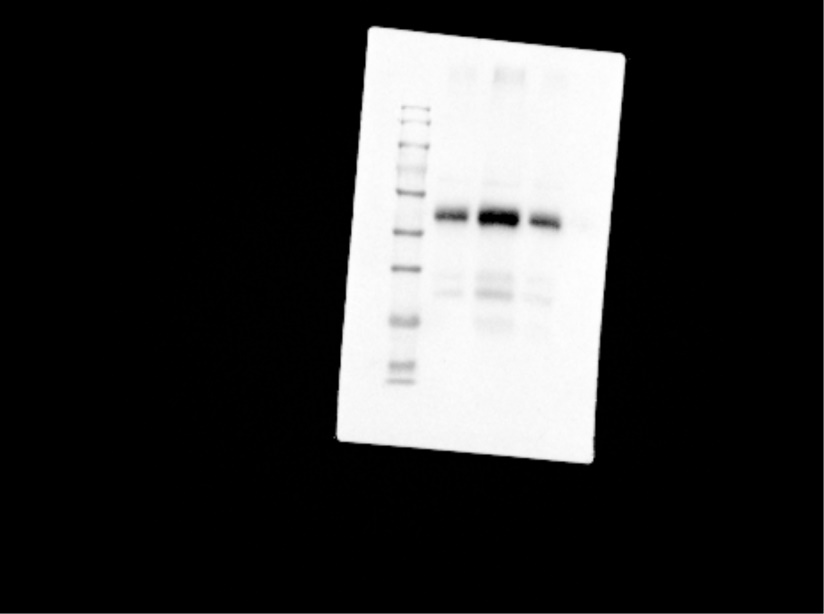


Full and uncropped western blots for Figure 5G-2 (1)


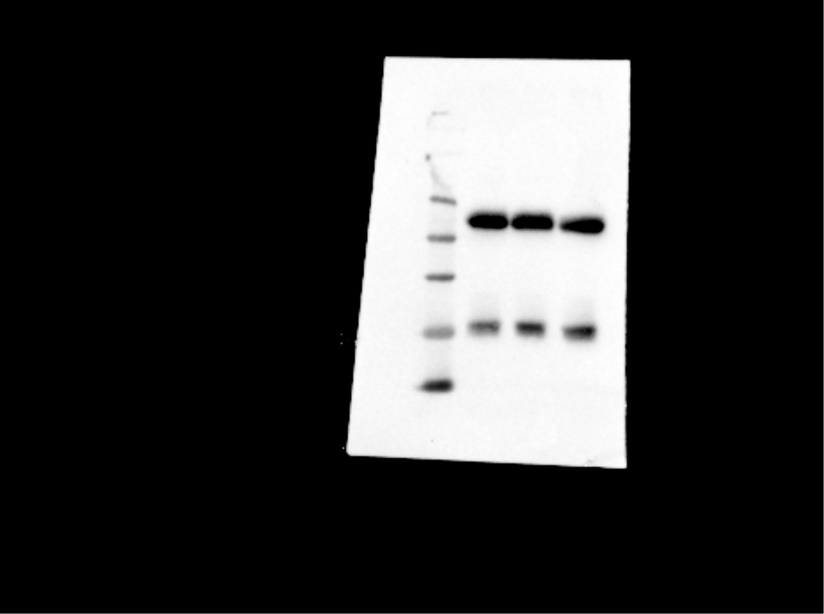


Full and uncropped western blots for Figure 5G-2 (2)


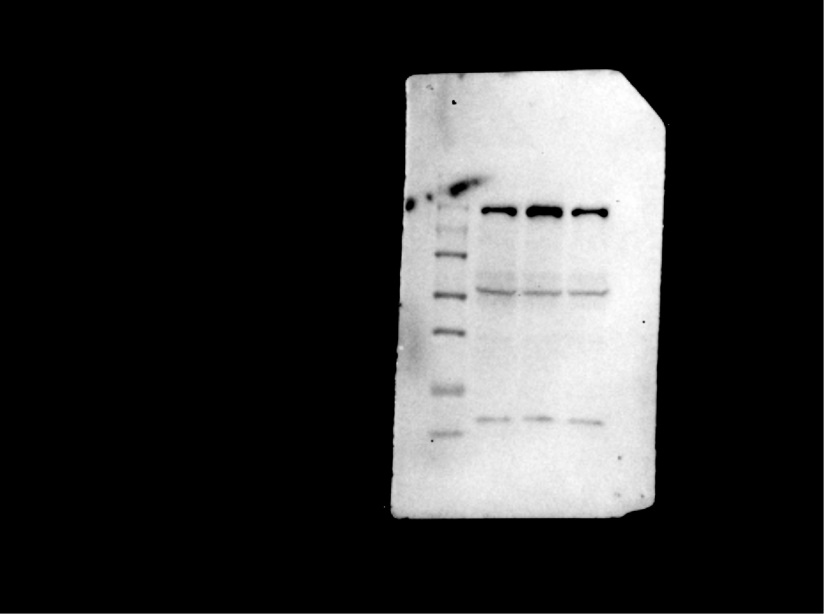


Full and uncropped western blots for Figure 5G-3 (1)


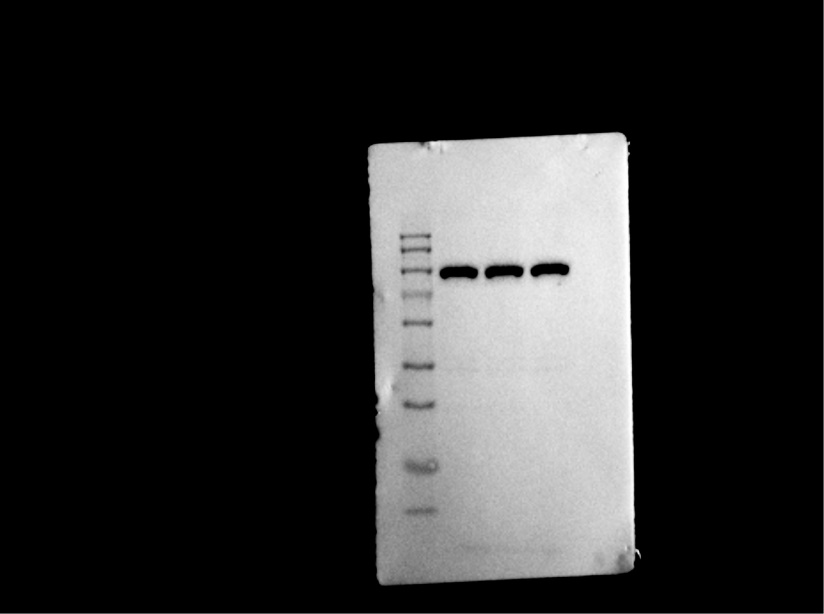


Full and uncropped western blots for Figure 5G-3 (2)


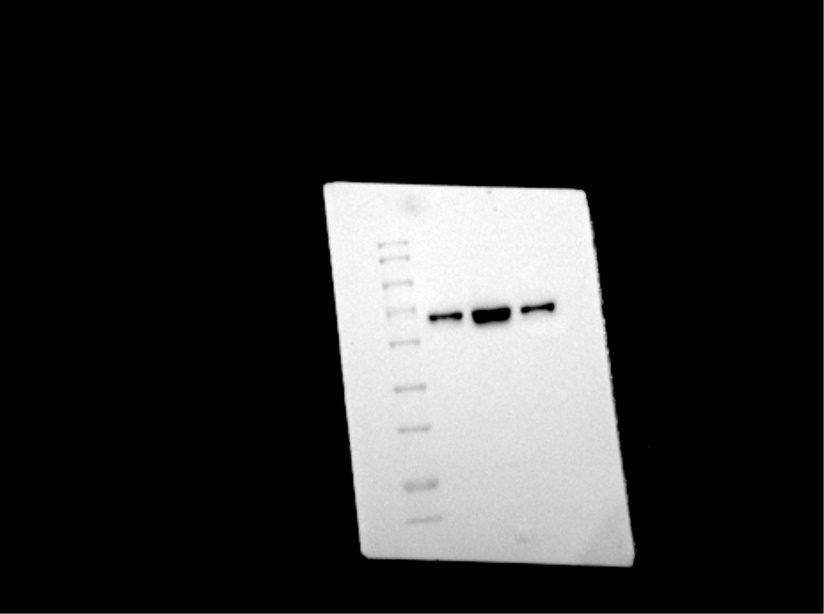


Full and uncropped western blots for Figure 5G-4-1


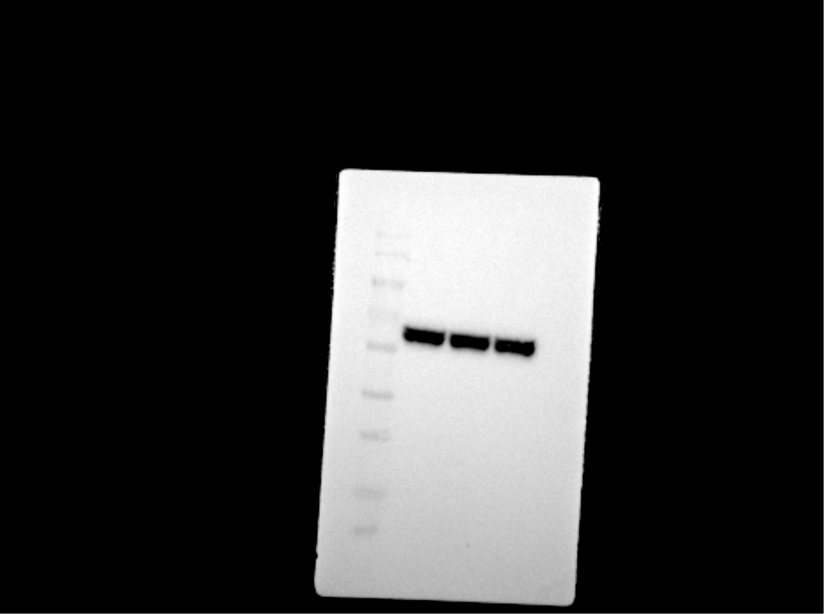


Full and uncropped western blots for Figure 5G-4-2


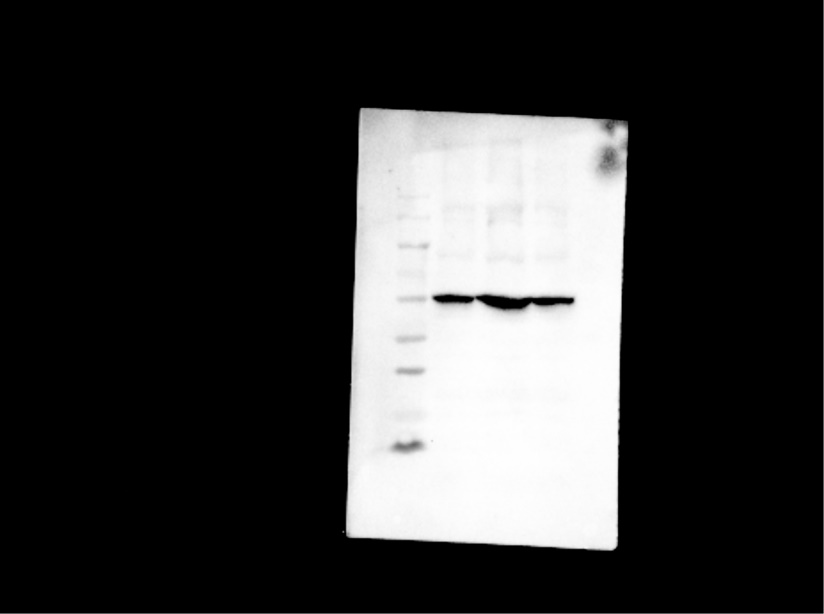


Full and uncropped western blots for Figure 5G-5


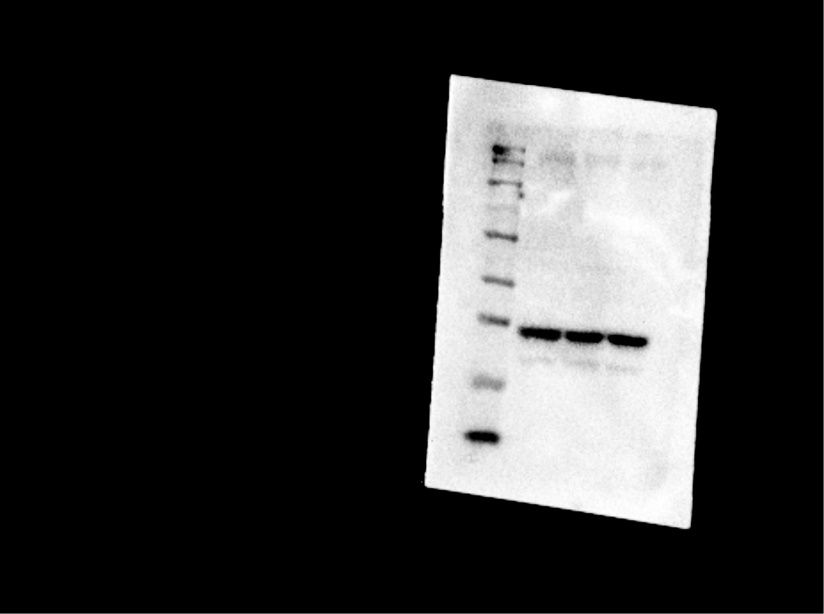


Full and uncropped western blots for Figure 5G-6 (1)


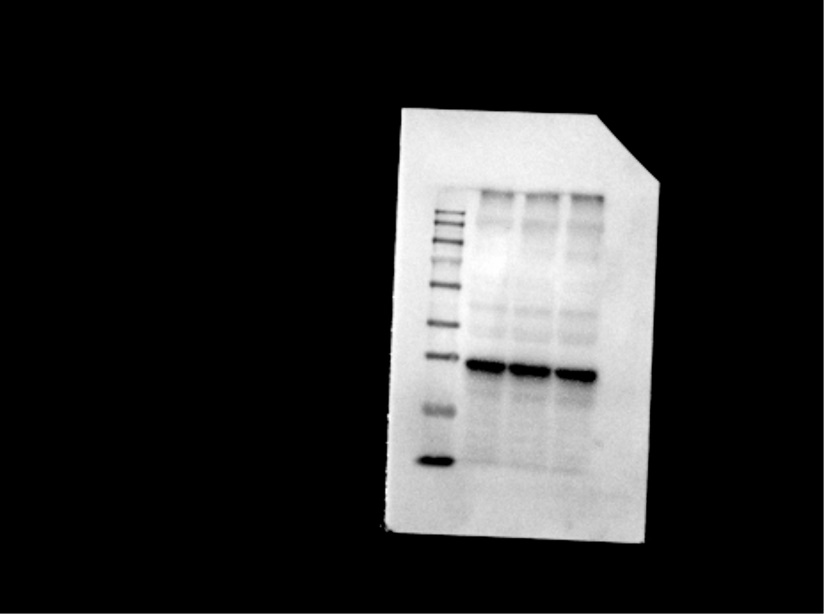


Full and uncropped western blots for Figure 5G-6 (2)


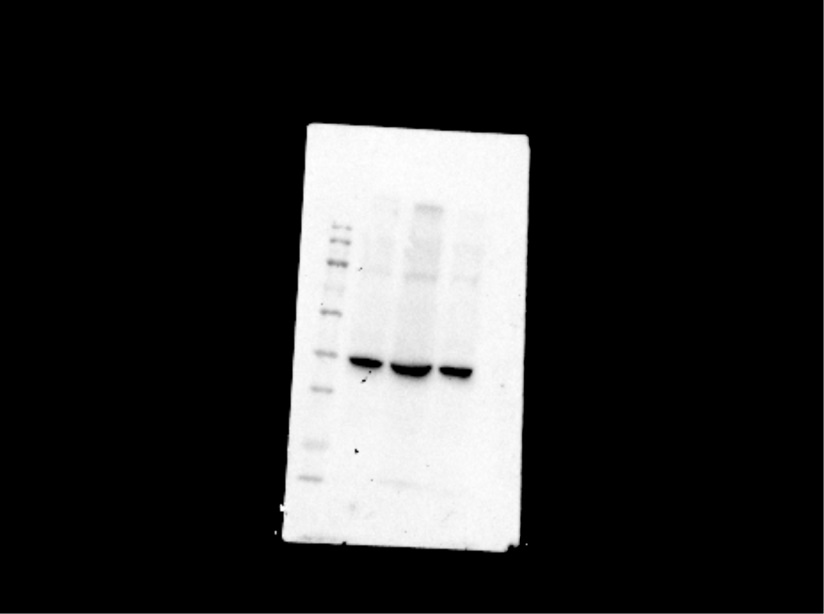


Full and uncropped western blots for Figure 5M-1


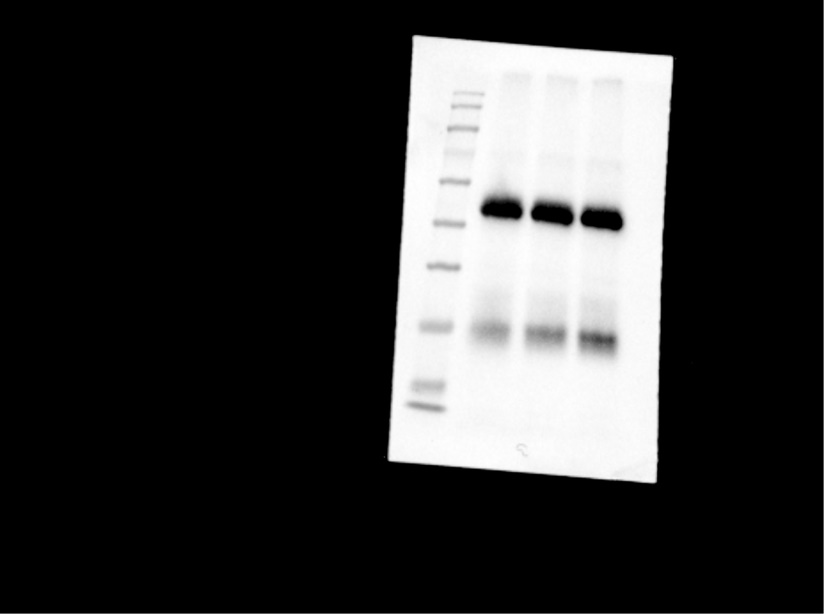


Full and uncropped western blots for Figure 5M-2


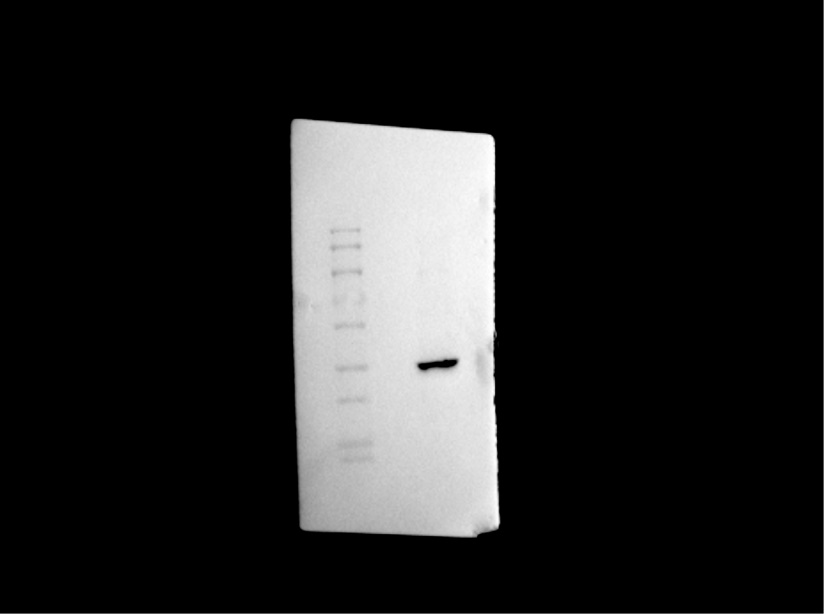


Full and uncropped western blots for Figure 6B


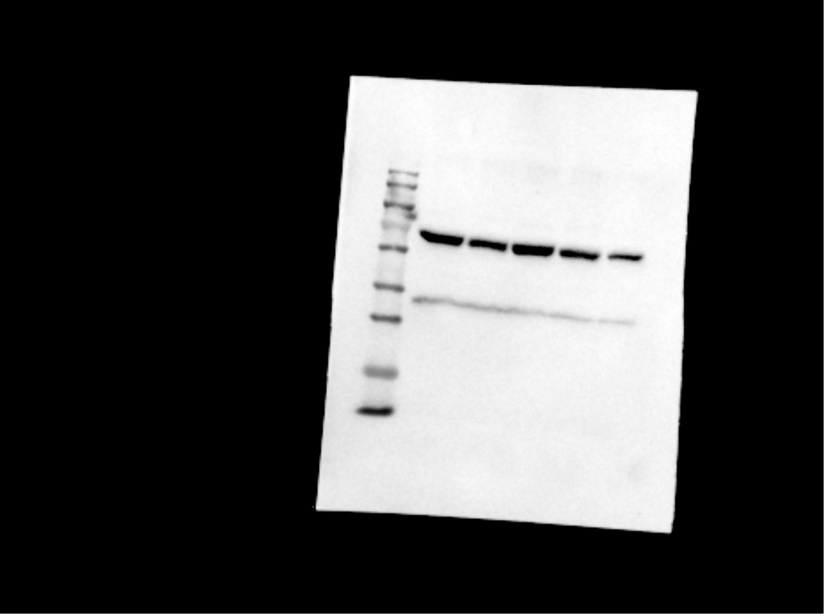


Full and uncropped western blots for Figure 7A-1 (1)


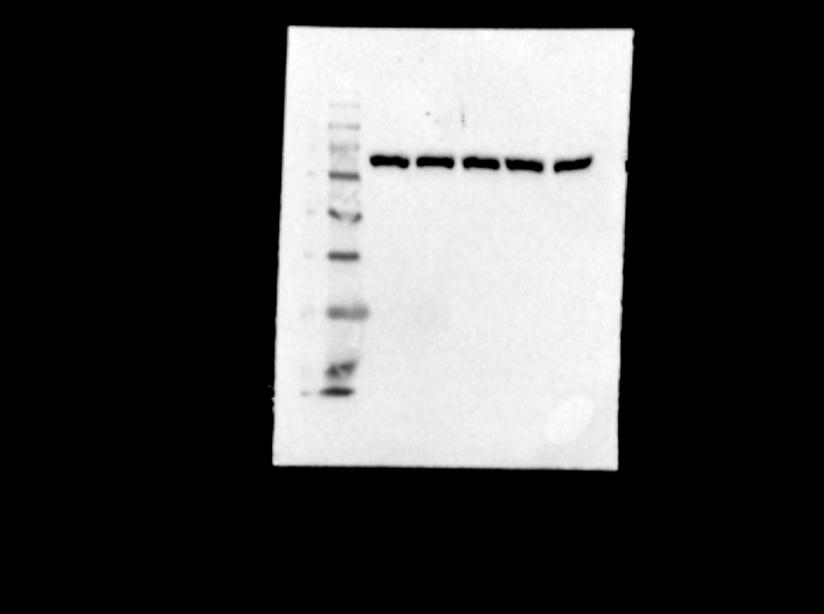


Full and uncropped western blots for Figure 7A-1 (2)


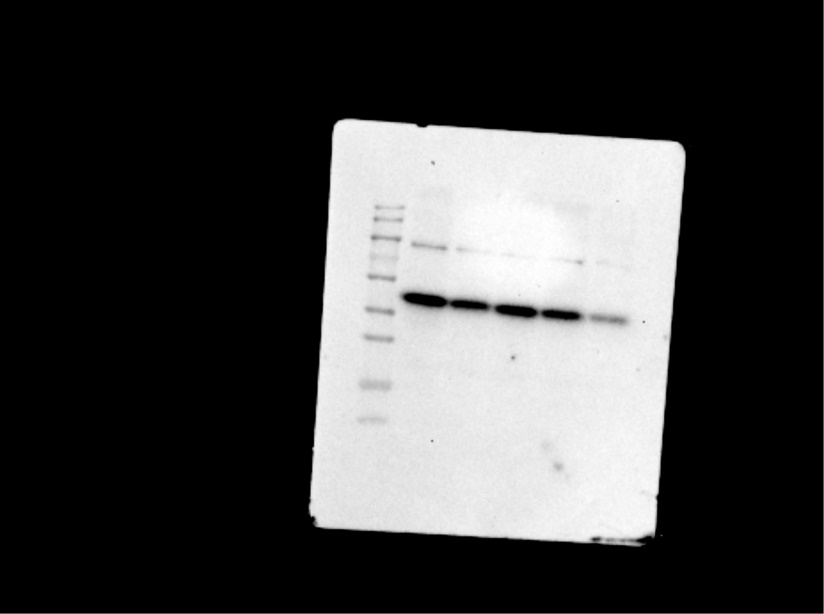


Full and uncropped western blots for Figure 7A-2 (1)


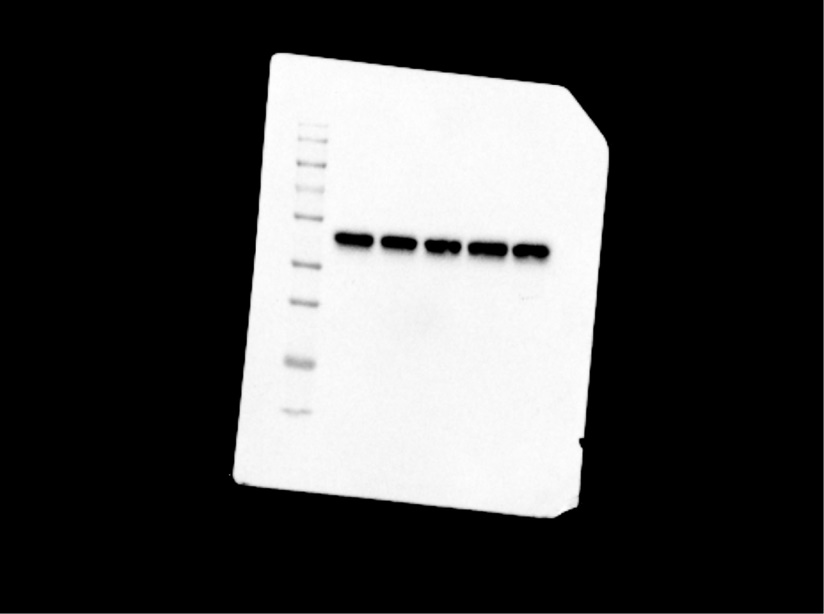


Full and uncropped western blots for Figure 7A-2 (2)


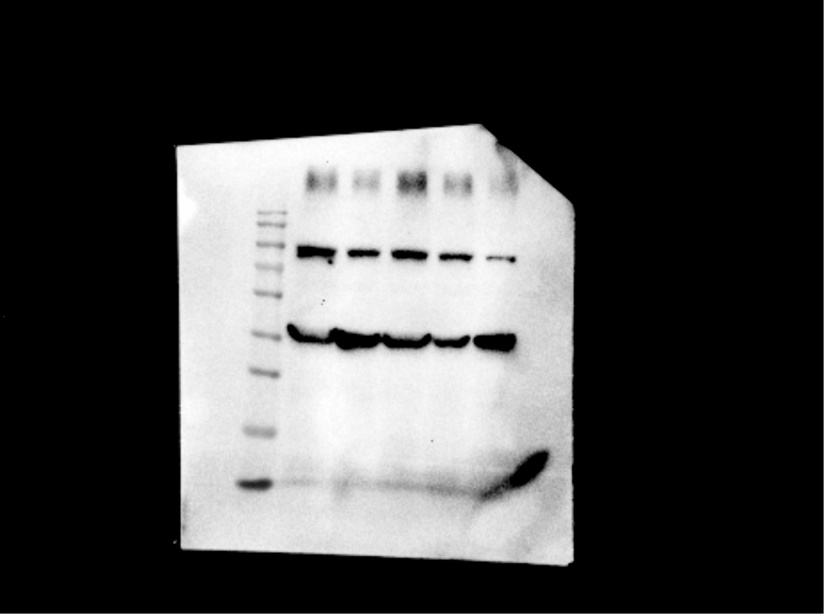


Full and uncropped western blots for Figure 7A-3 (1)


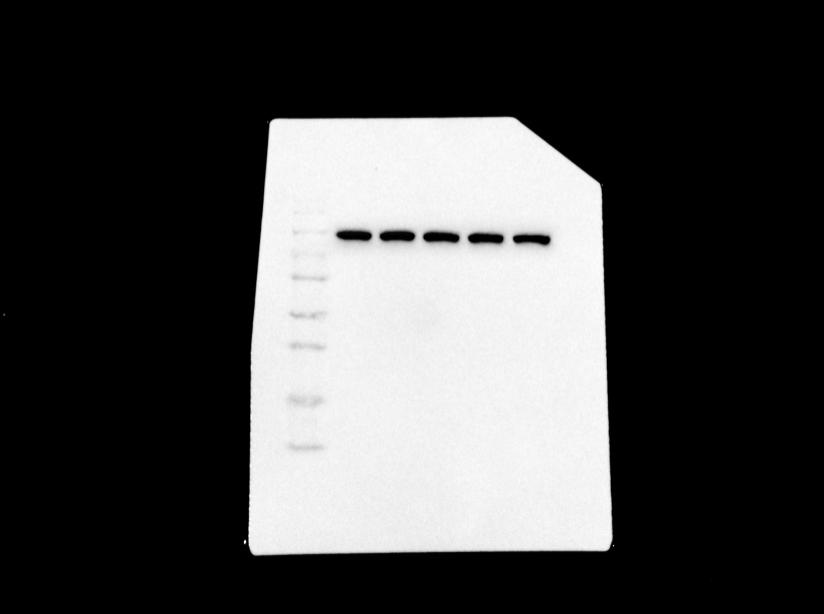


Full and uncropped western blots for Figure 7A-3 (2)


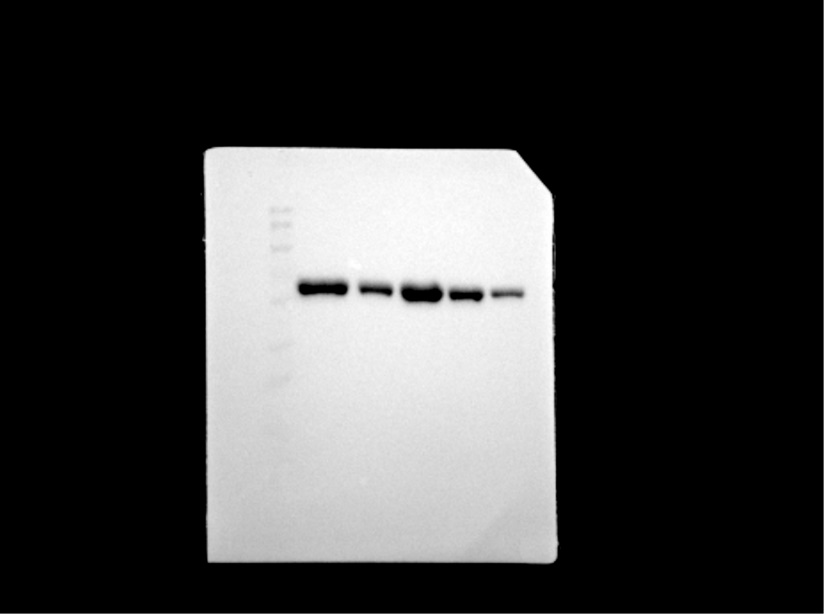


Full and uncropped western blots for Figure 7A-4-1


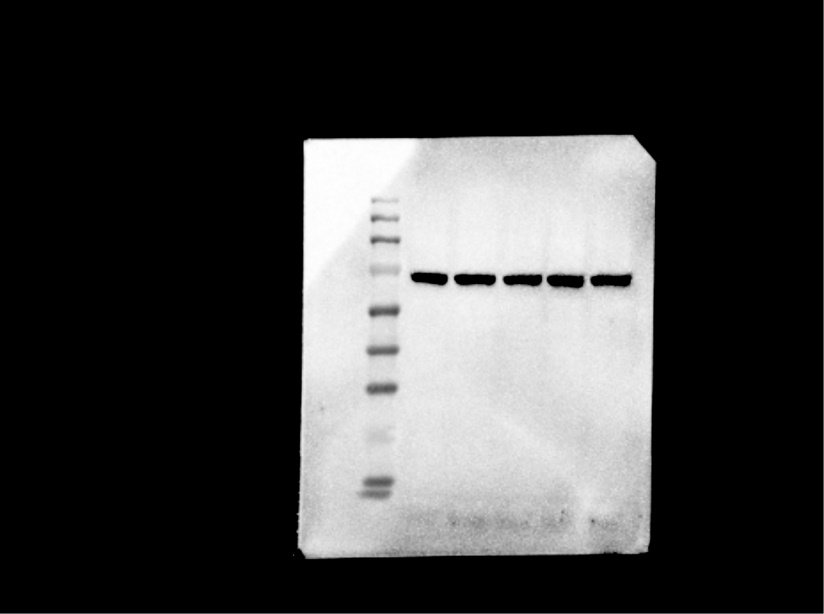


Full and uncropped western blots for Figure 7A-4-2


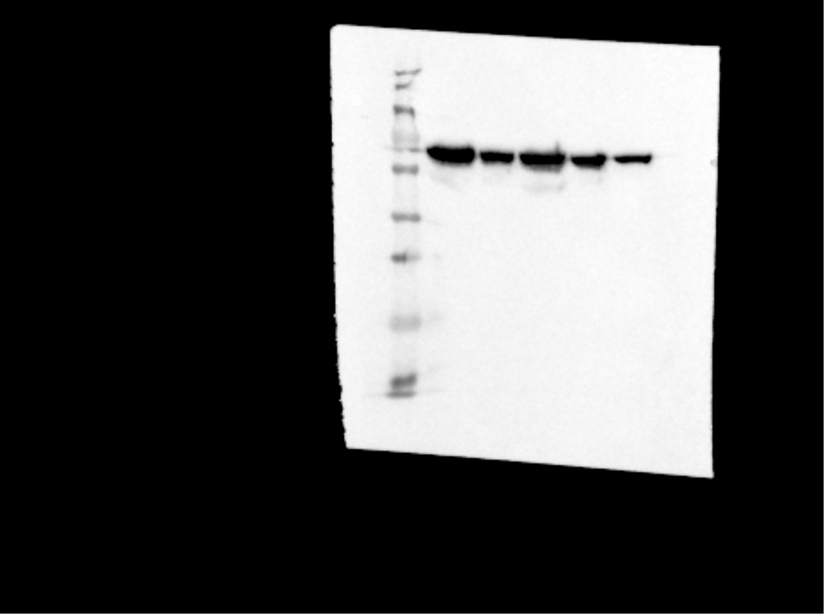


Full and uncropped western blots for Figure 7A-5


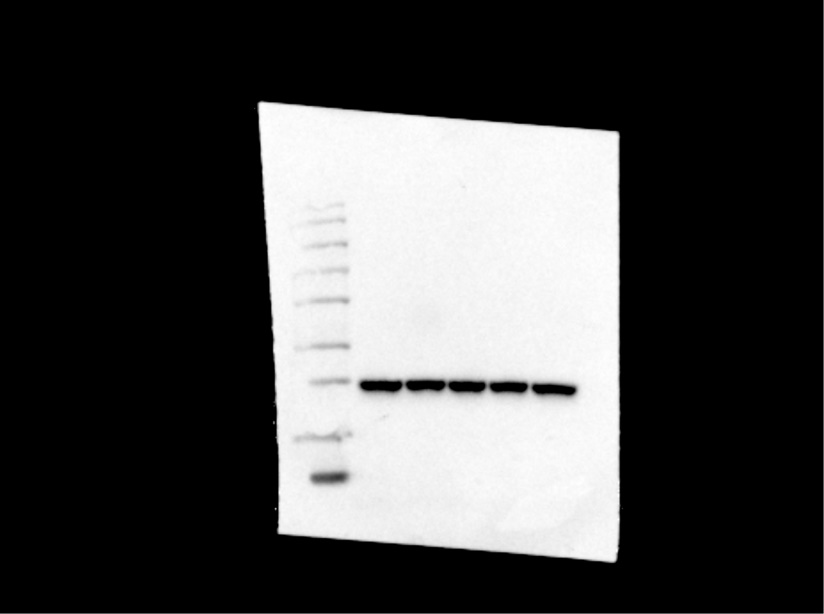


Full and uncropped western blots for Figure 7A-6 (1)


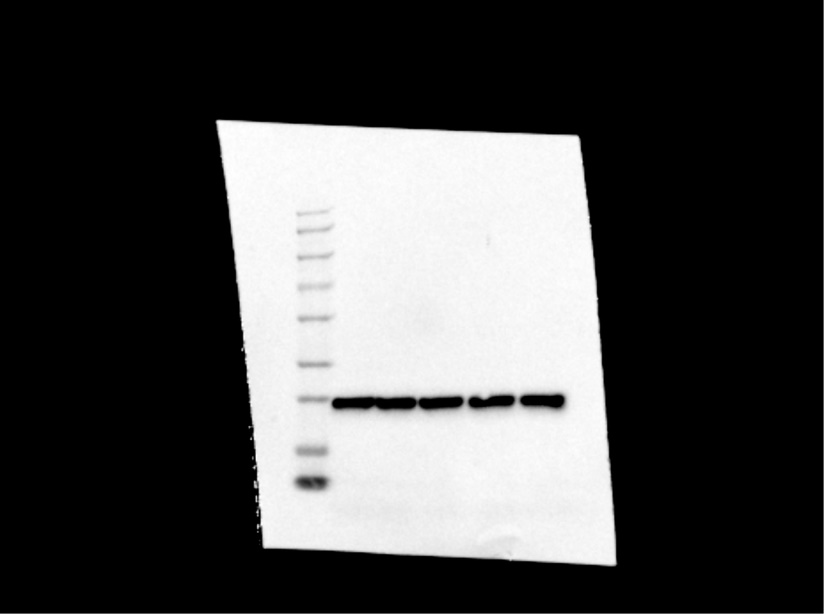


Full and uncropped western blots for Figure 7A-6 (2)


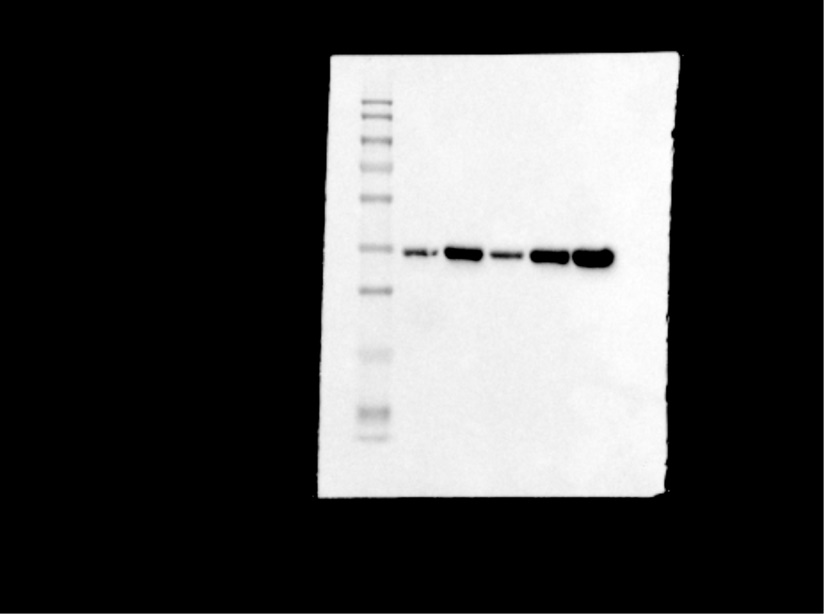


Full and uncropped western blots for Figure 7I-1


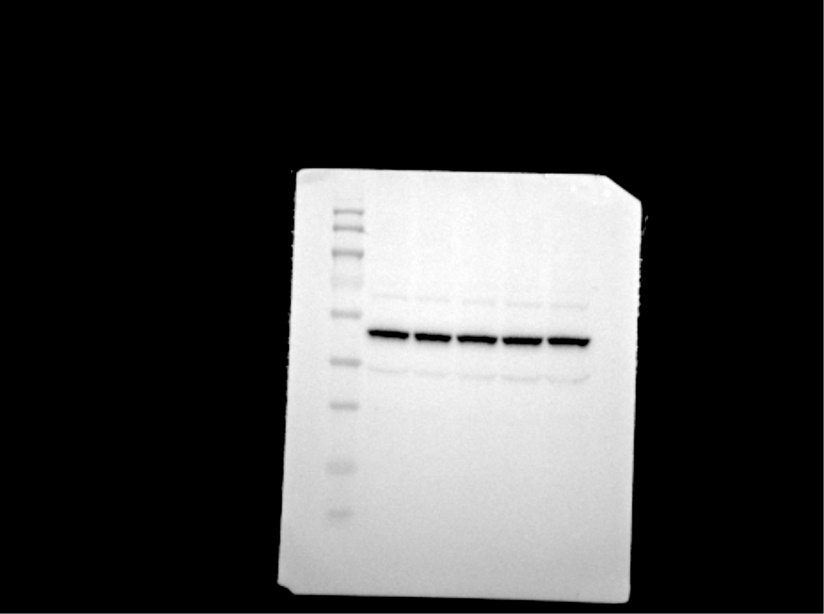


Full and uncropped western blots for Figure 7I-2


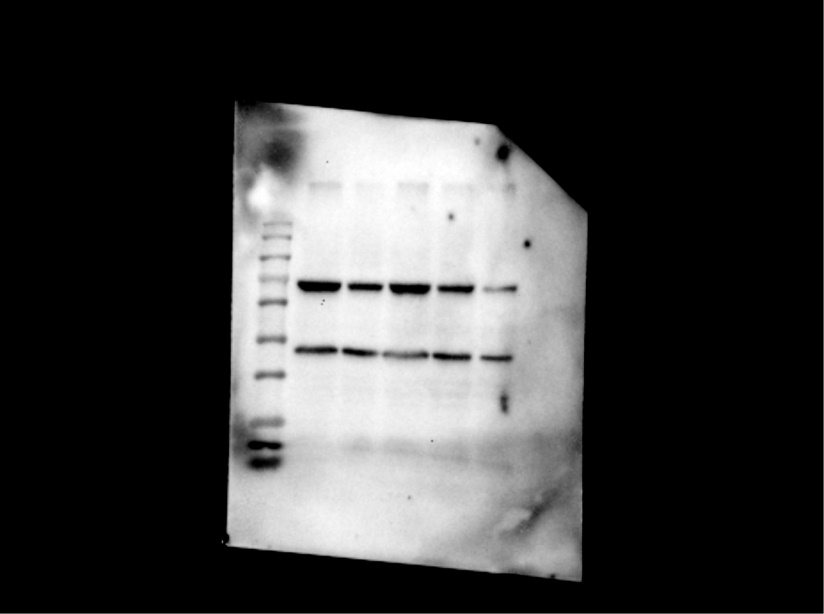


Full and uncropped western blots for Figure 8D-1 (1)


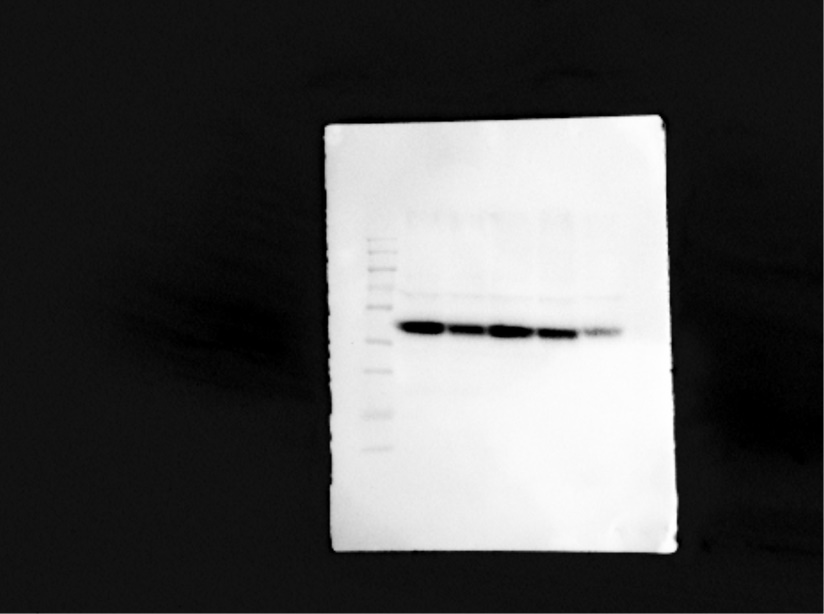


Full and uncropped western blots for Figure 8D-2 (1)


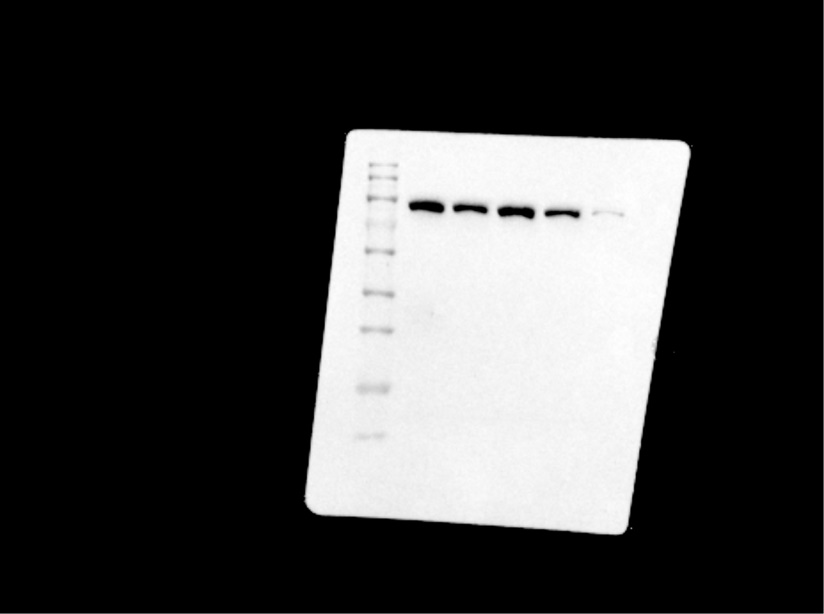


Full and uncropped western blots for Figure 8D-3 (1)


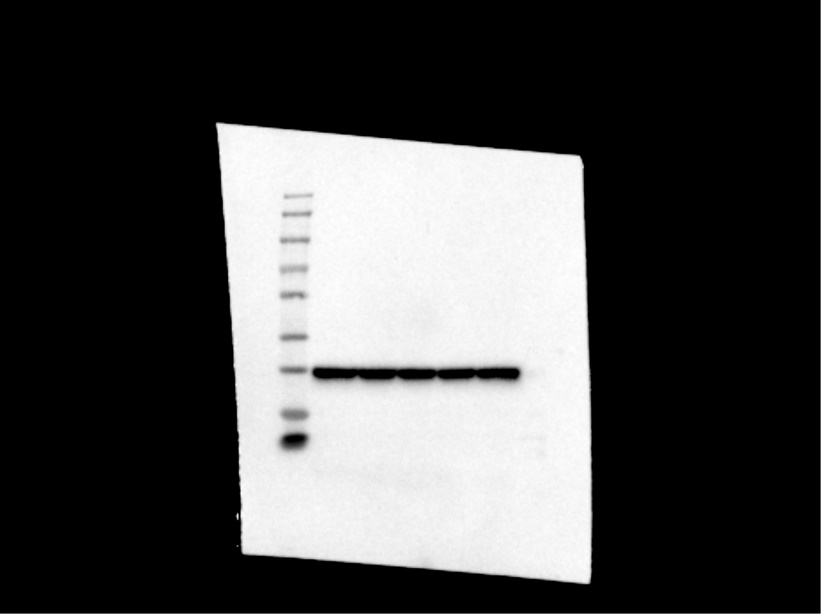


Full and uncropped western blots for Figure 8D-4 (1)


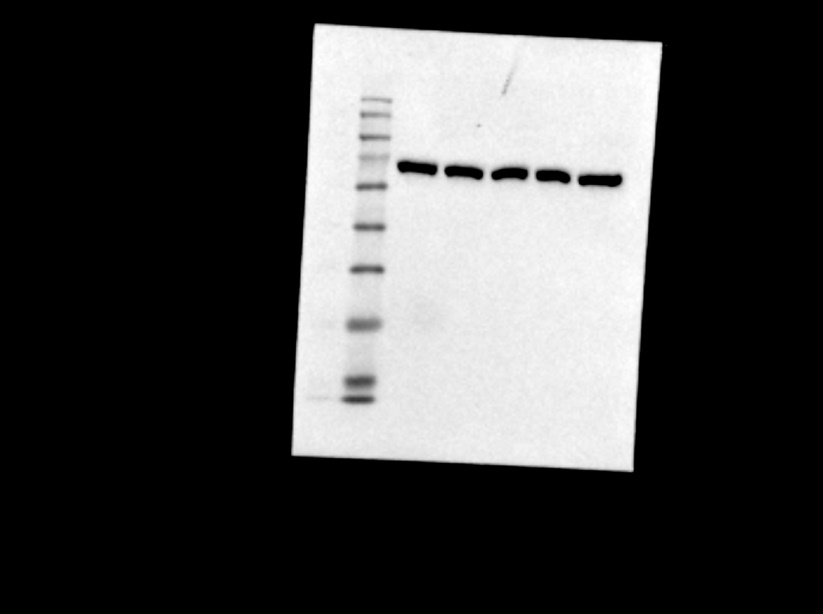


Full and uncropped western blots for Figure 8D-5


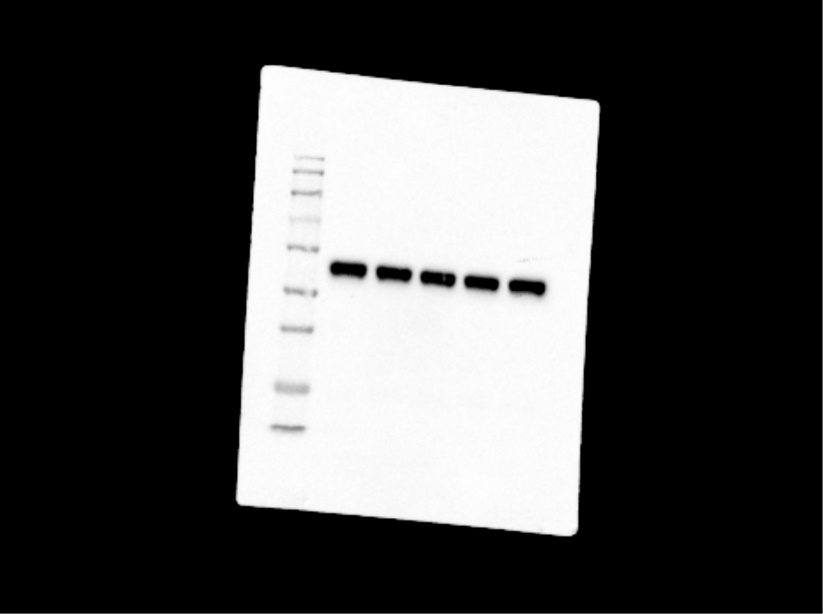


Full and uncropped western blots for Figure 8D-6


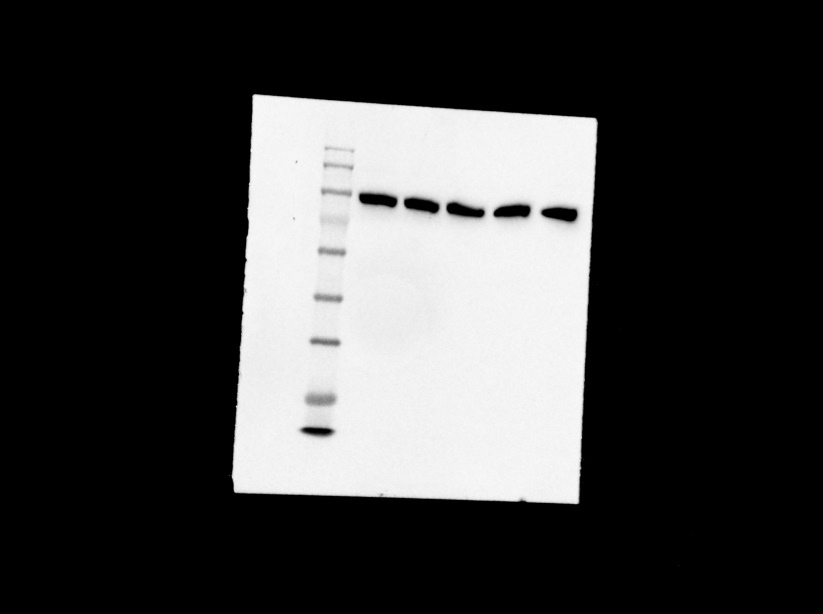


Full and uncropped western blots for Figure 8D-7


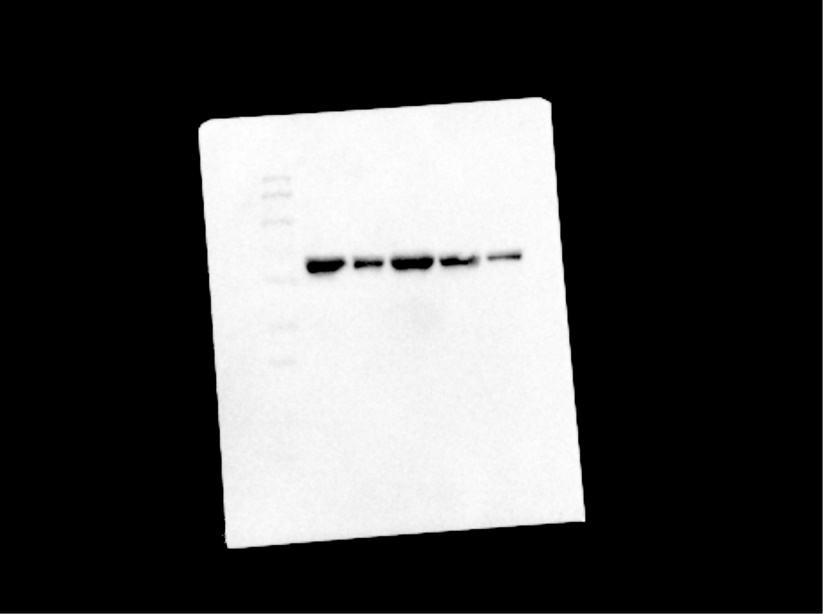


Full and uncropped western blots for Figure 8D-8


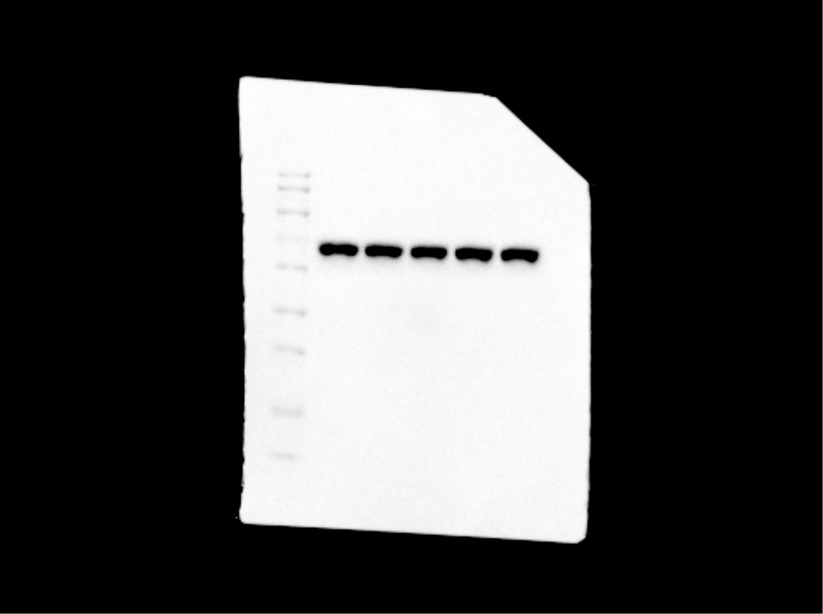


Full and uncropped western blots for Figure 8D-9


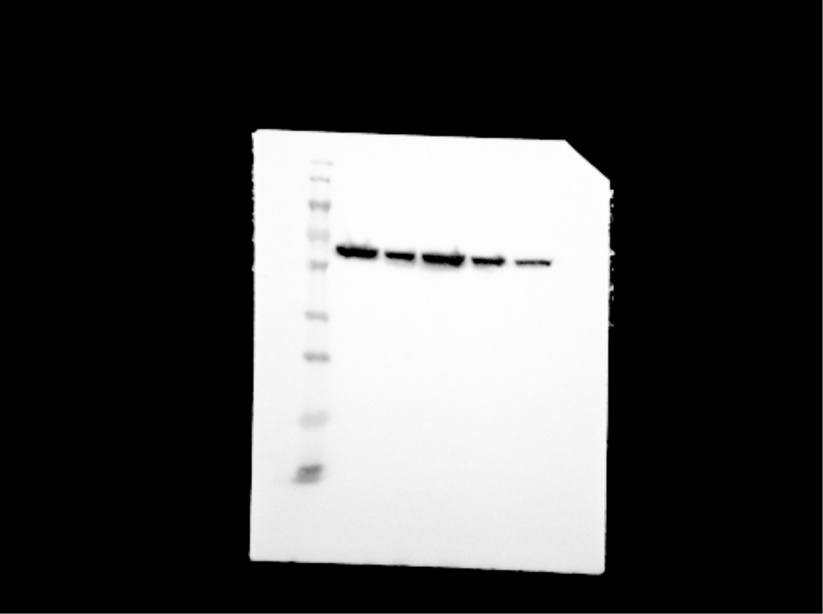


Full and uncropped western blots for Figure 8D-10


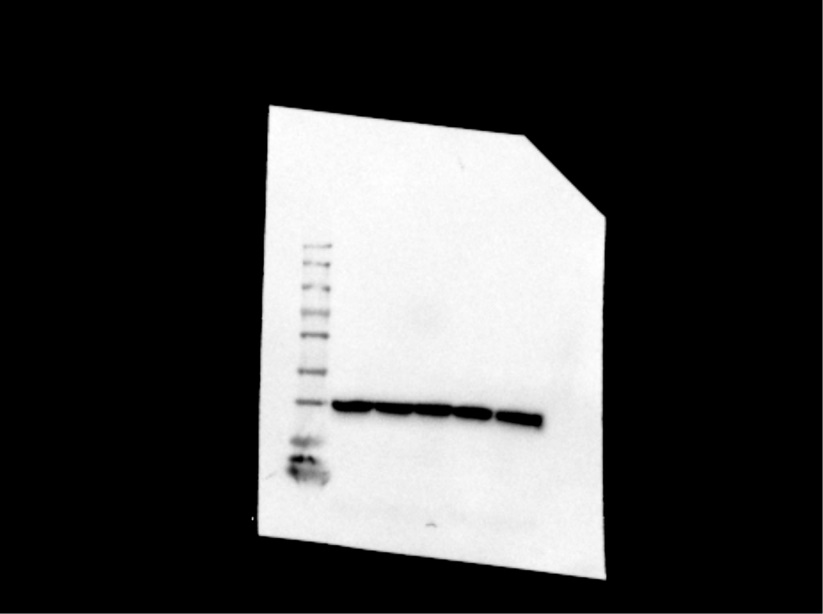


Full and uncropped western blots for Figure 8D-11


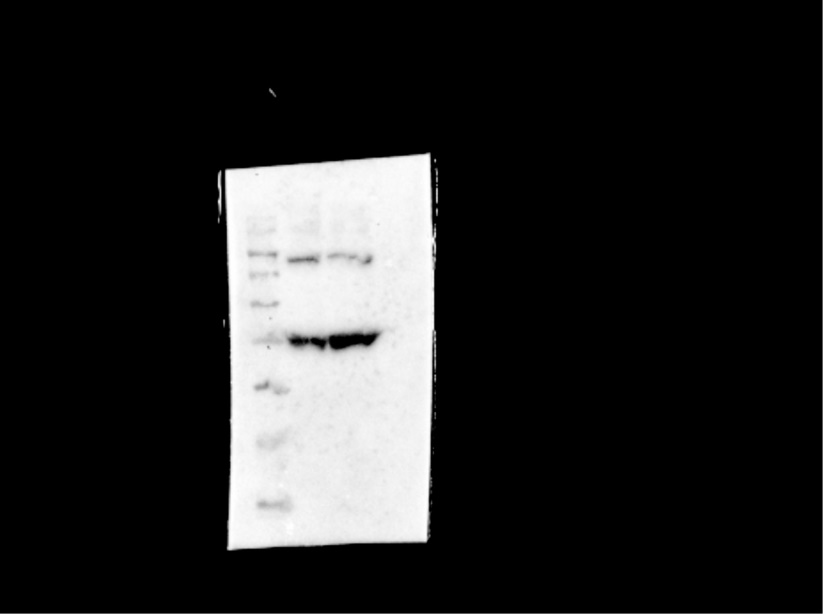


Full and uncropped western blots for Figure S6G-1


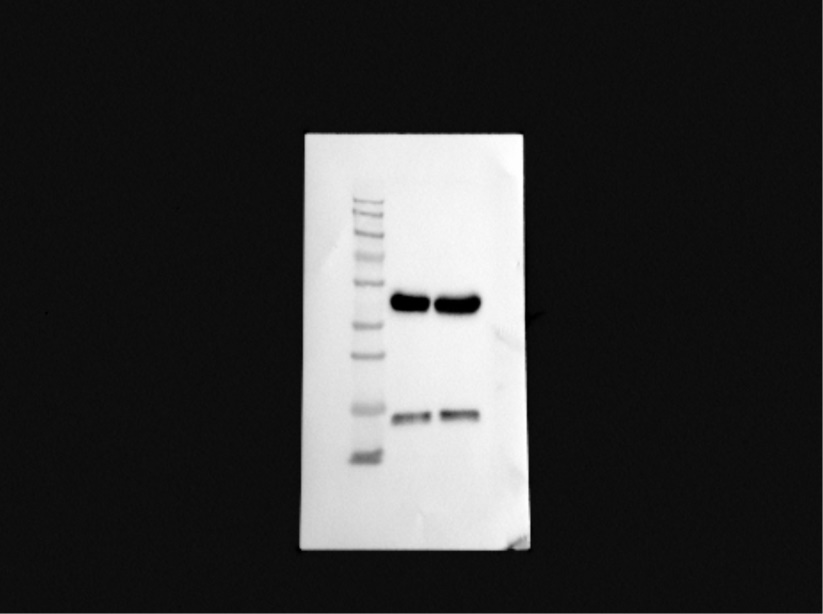


Full and uncropped western blots for Figure S6G-2


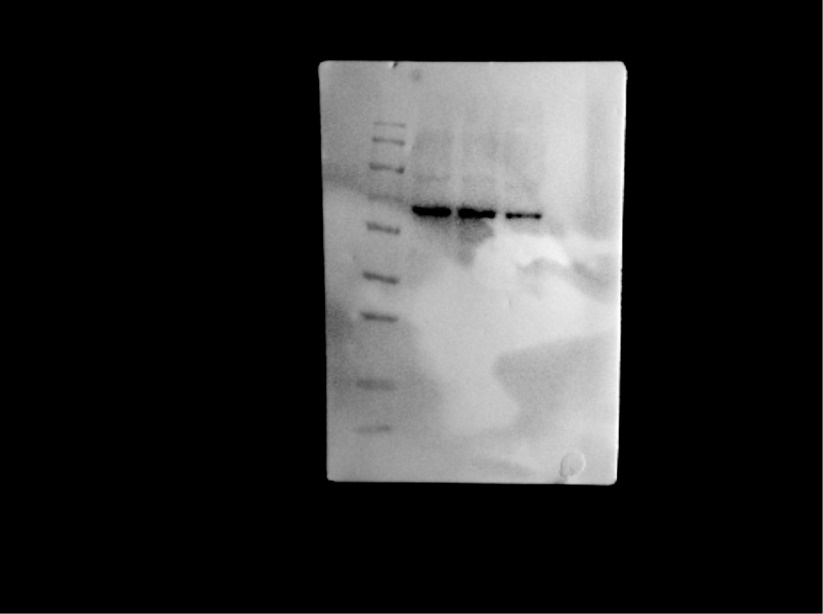


Full and uncropped western blots for Figure S9A-1 (1)


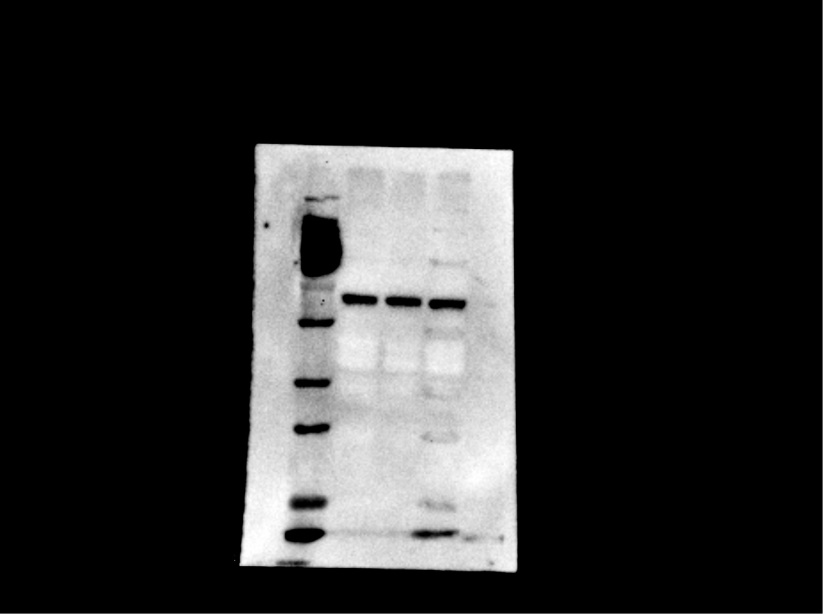


Full and uncropped western blots for Figure S9A-1 (2)


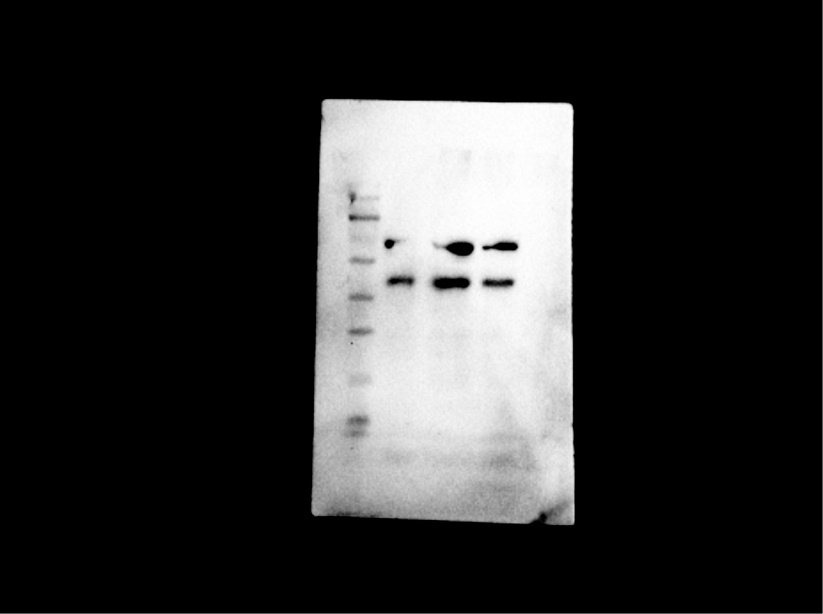


Full and uncropped western blots for Figure S9A-2 (1)


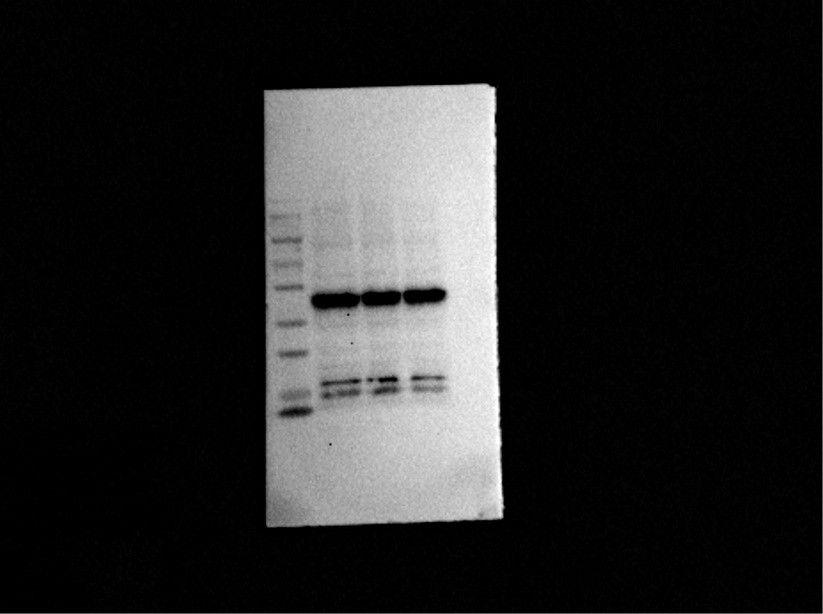


Full and uncropped western blots for Figure S9A-2 (2)


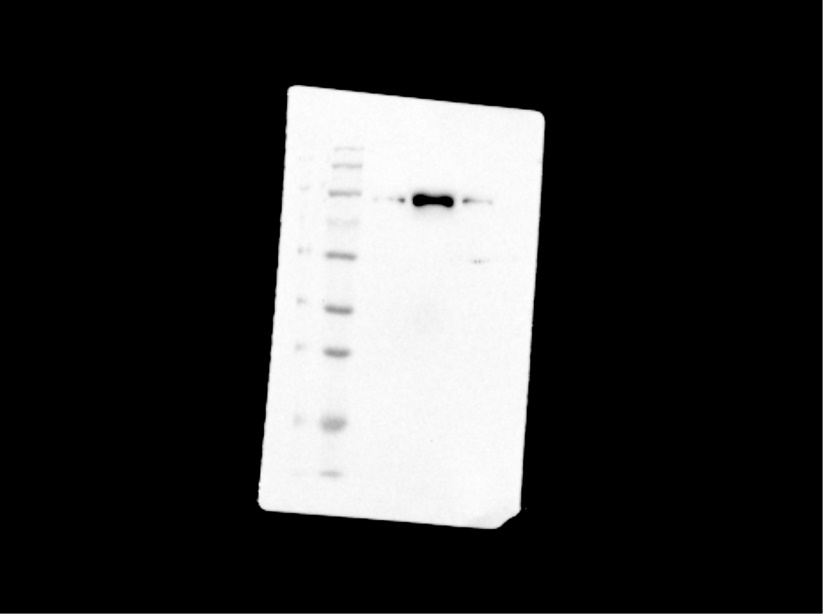


Full and uncropped western blots for Figure S9A-3 (1)


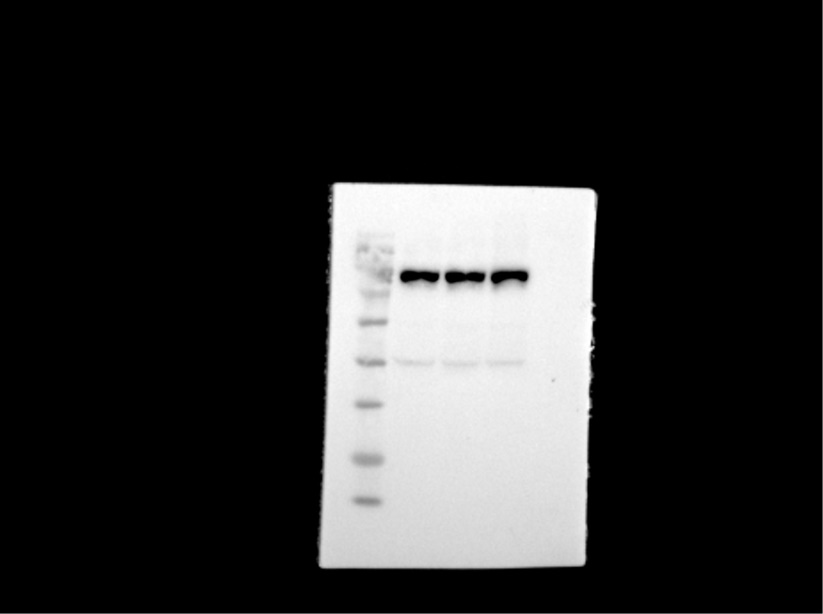


Full and uncropped western blots for Figure S9A-3 (2)


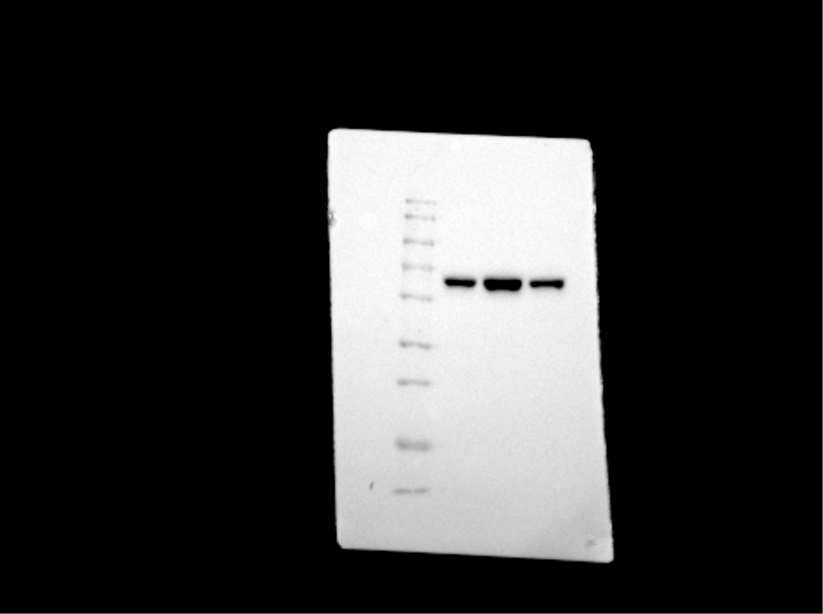


Full and uncropped western blots for Figure S9A-4-1


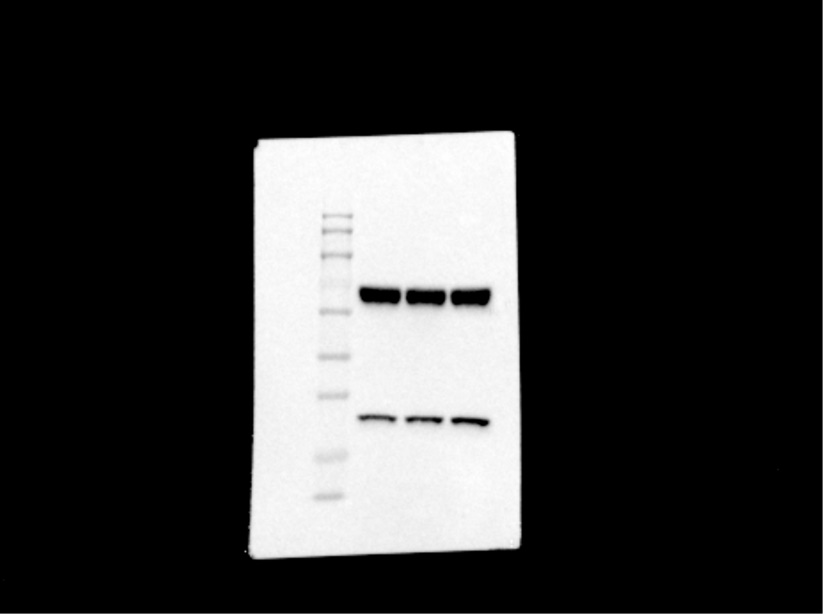


Full and uncropped western blots for Figure S9A-4-2


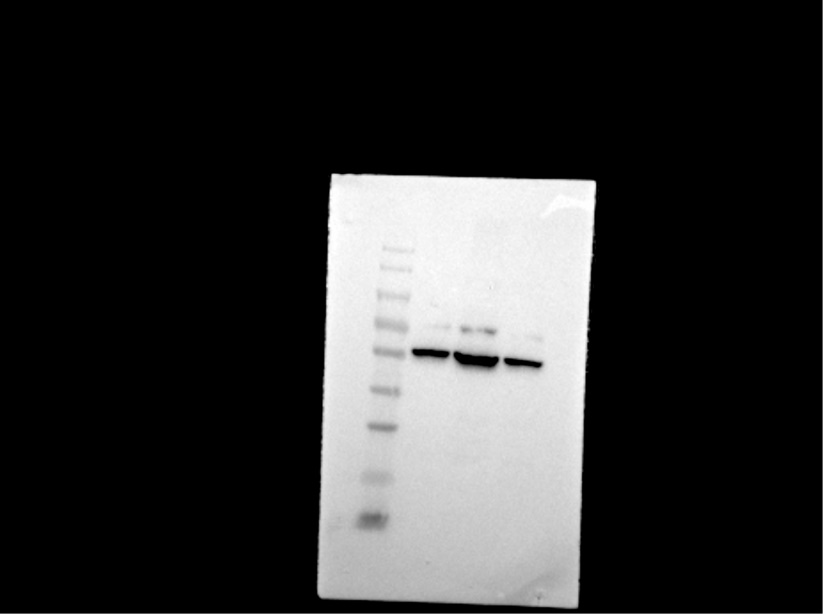


Full and uncropped western blots for Figure S9A-5


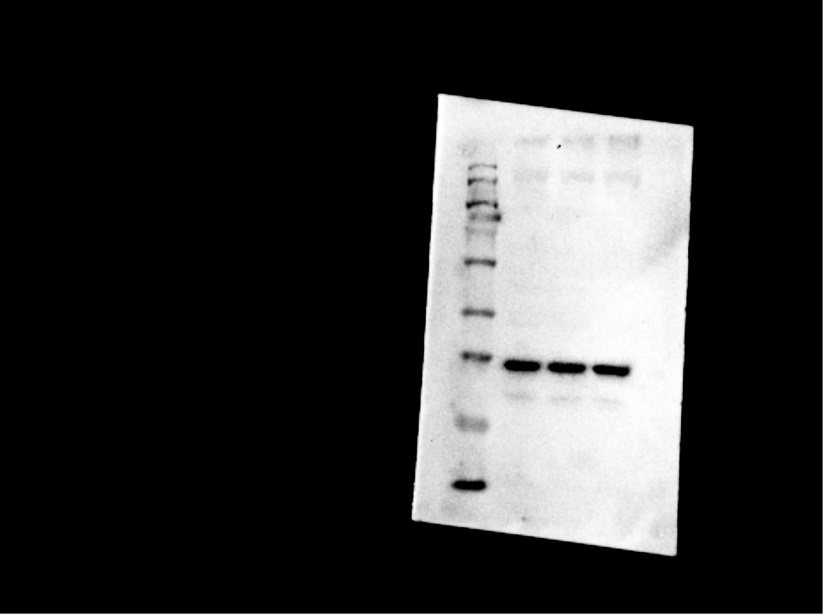


Full and uncropped western blots for Figure S9A-6 (1)


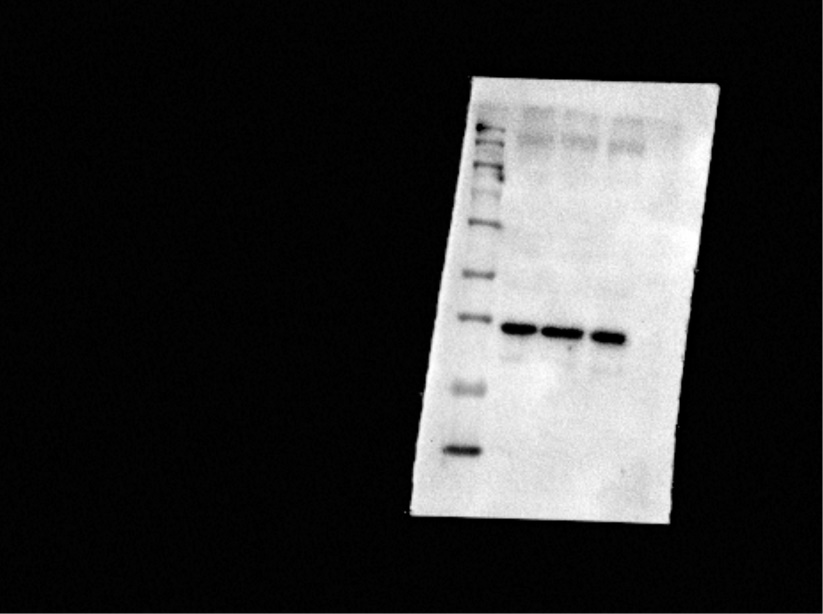


Full and uncropped western blots for Figure S9A-6 (2)


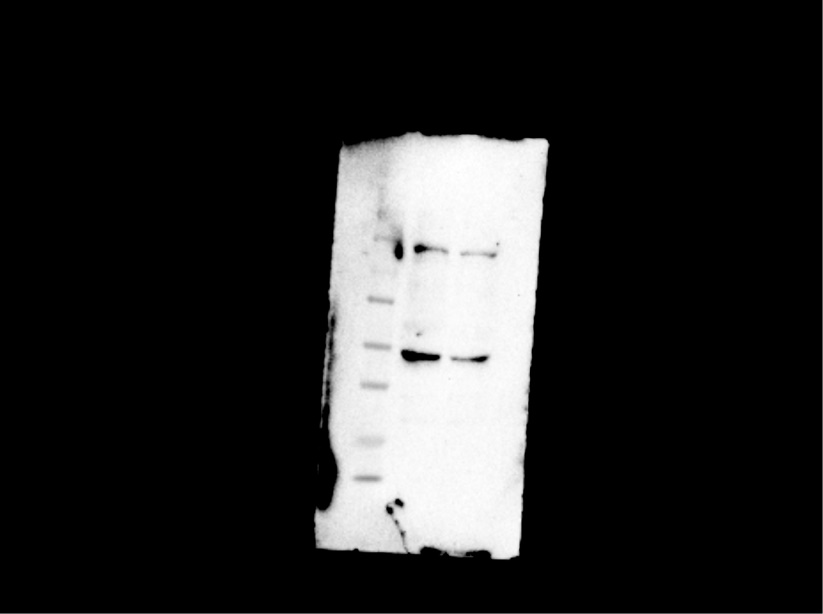


Full and uncropped western blots for Figure S11C-1


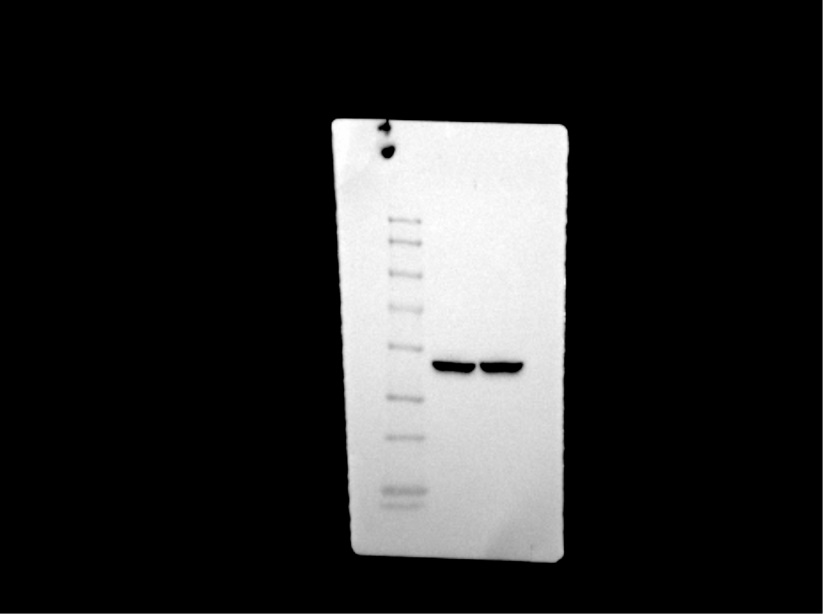


Full and uncropped western blots for Figure S11C-2


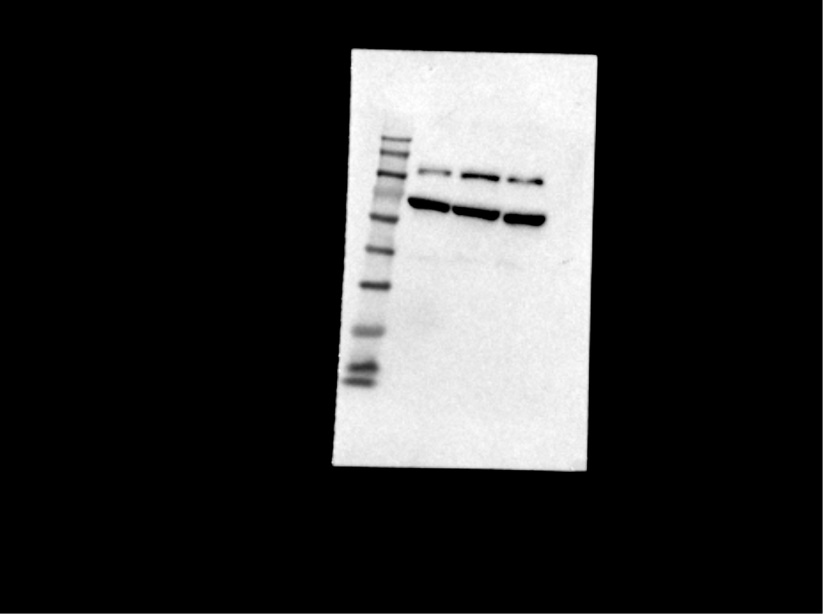


Full and uncropped western blots for Figure S11E-1 (2)


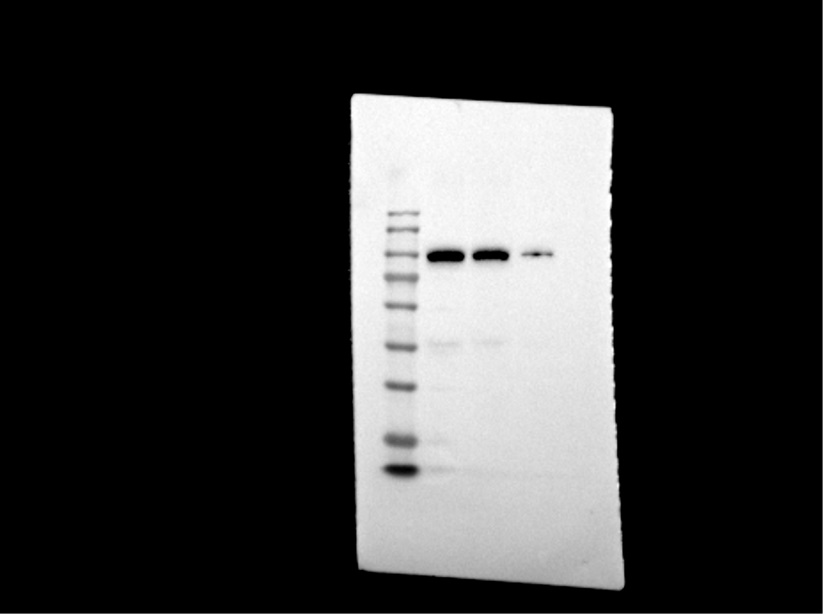


Full and uncropped western blots for Figure S11E-1


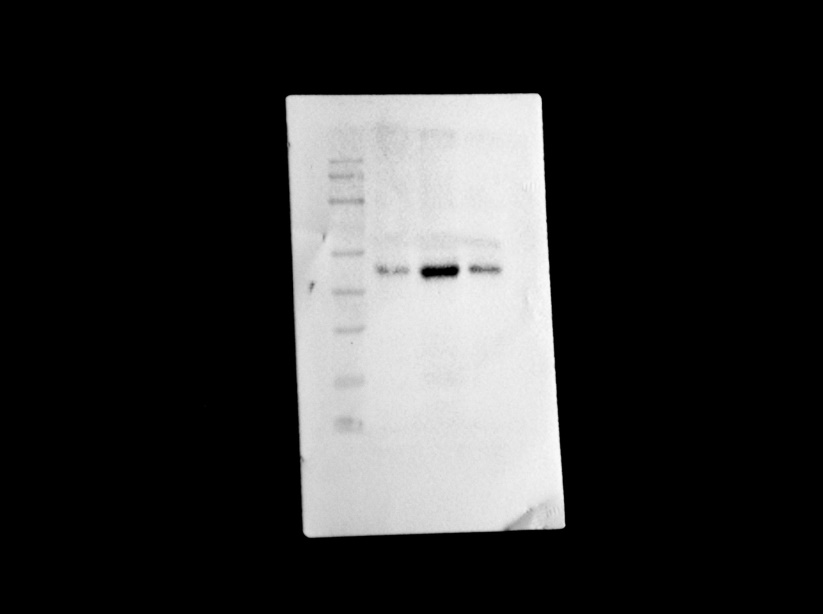


Full and uncropped western blots for Figure S11E-2 (1)


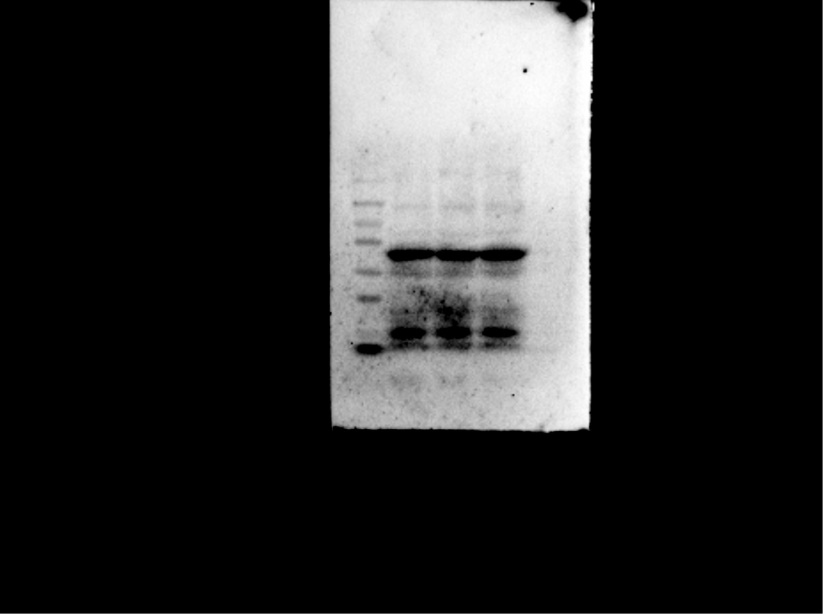


Full and uncropped western blots for Figure S11E-2 (2)


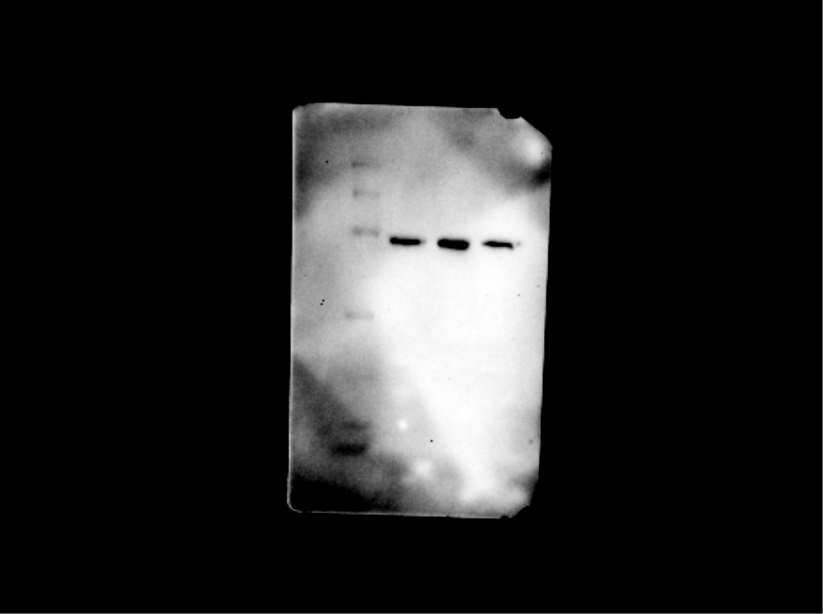


Full and uncropped western blots for Figure S11E-3 (1)


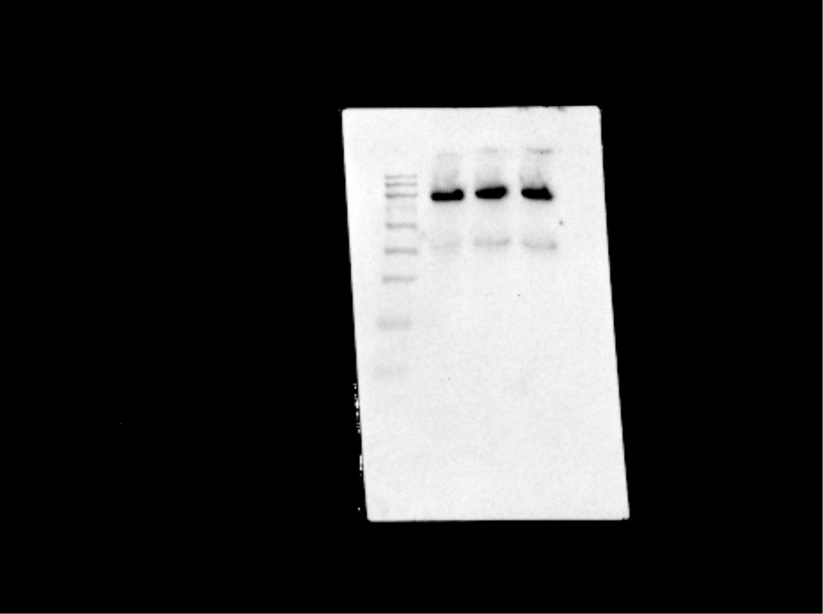


Full and uncropped western blots for Figure S11E-3 (2)


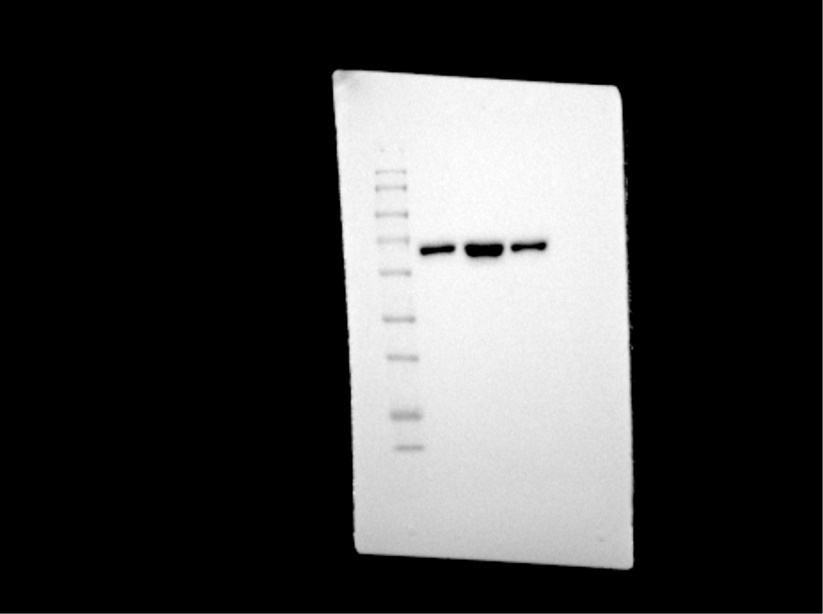


Full and uncropped western blots for Figure S11E-4-1


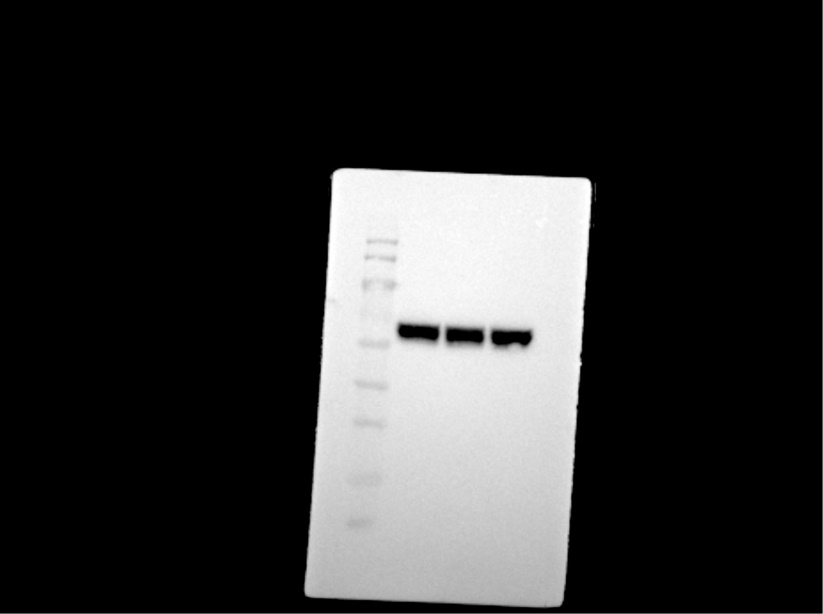


Full and uncropped western blots for Figure S11E-4-2


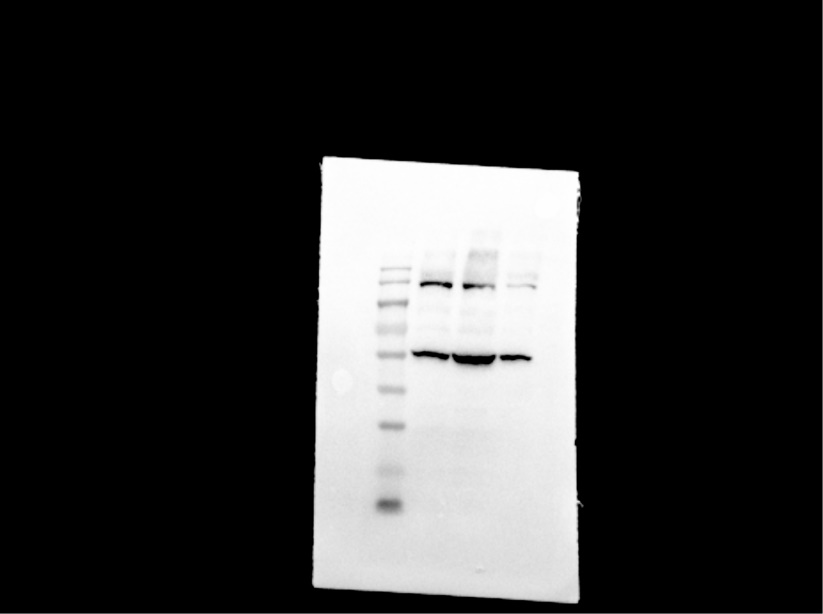


Full and uncropped western blots for Figure S11E-5


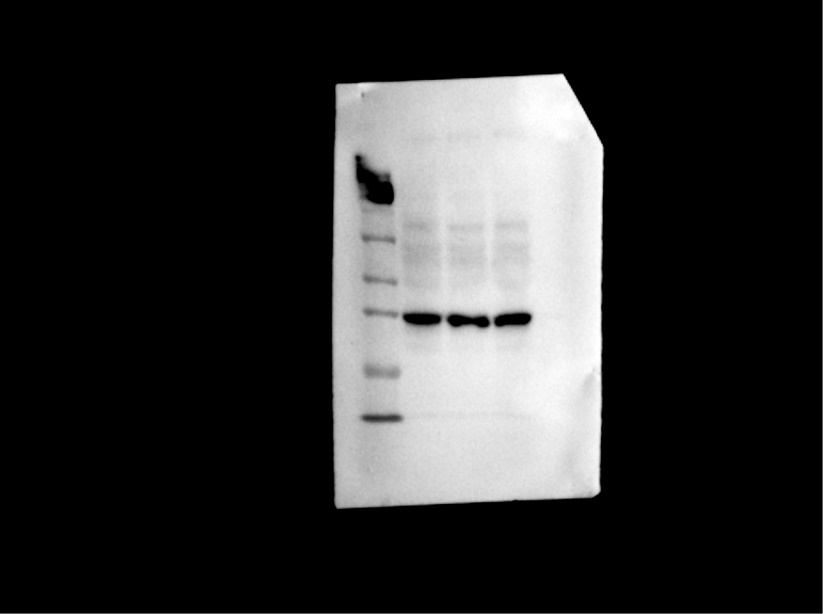


Full and uncropped western blots for Figure S11E-6 (1)


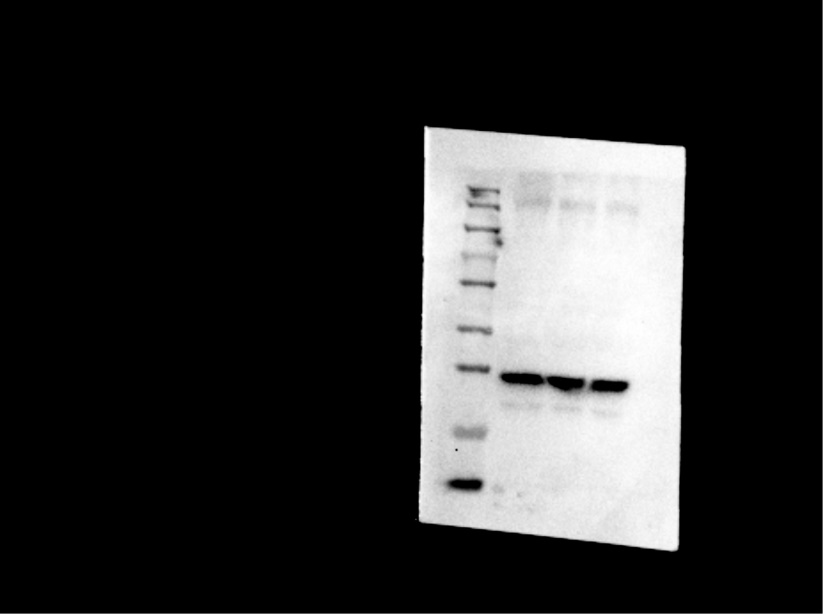


Full and uncropped western blots for Figure S11E-6 (2)


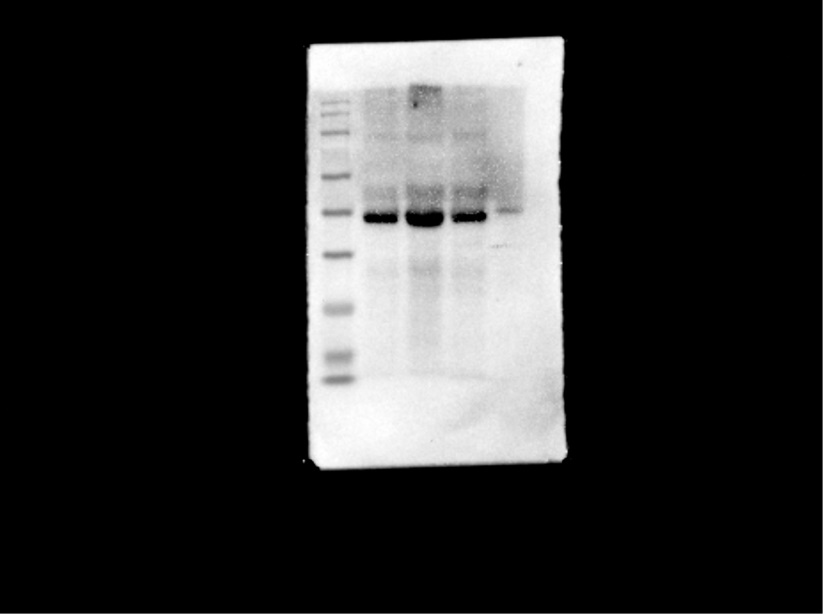


Full and uncropped western blots for Figure S11K-1


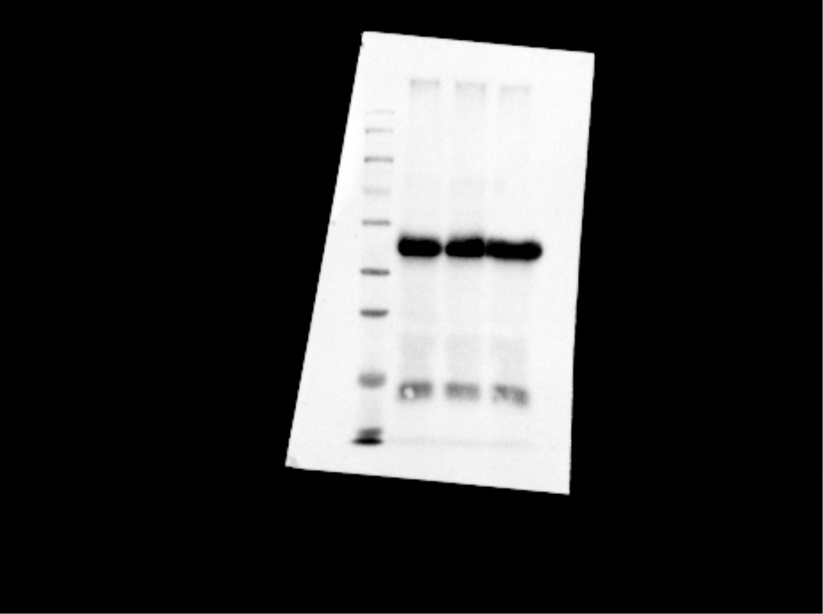


Full and uncropped western blots for Figure S11K-2


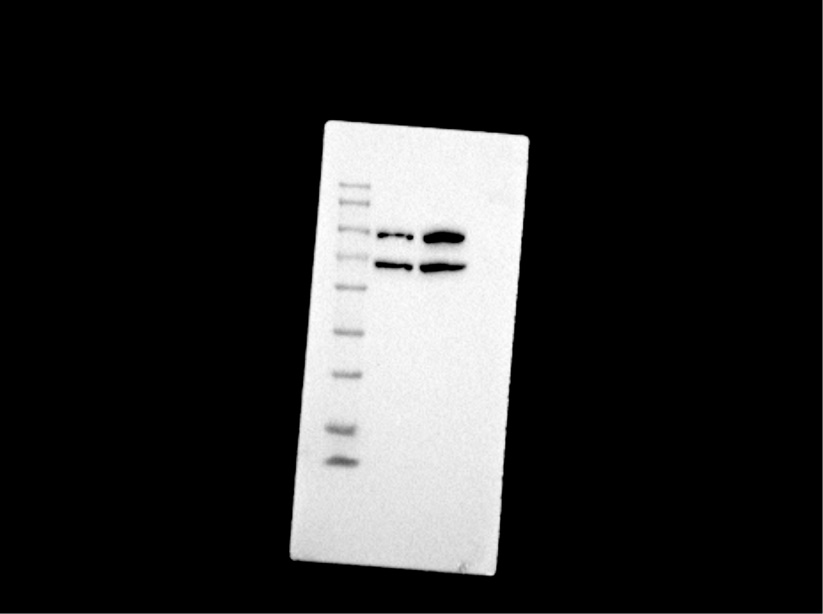


Full and uncropped western blots for Figure S12C-1


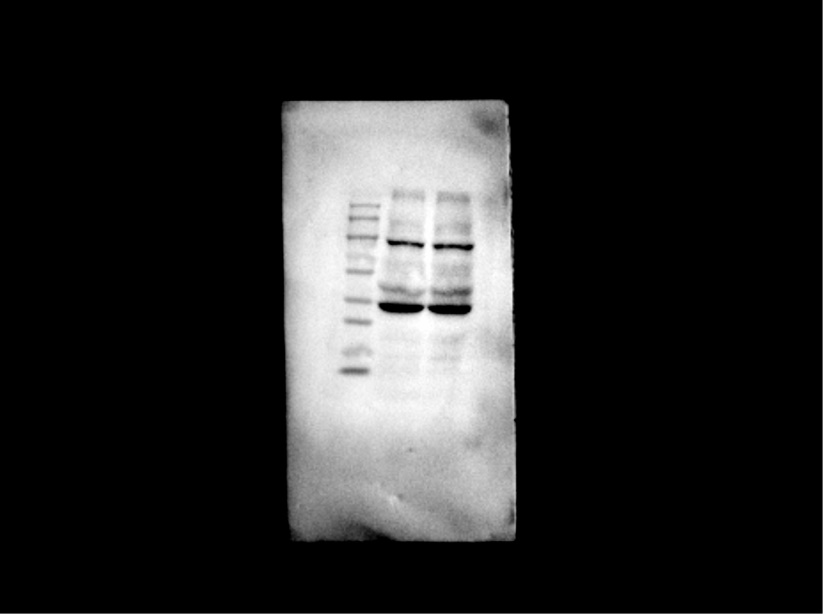


Full and uncropped western blots for Figure S12C-2


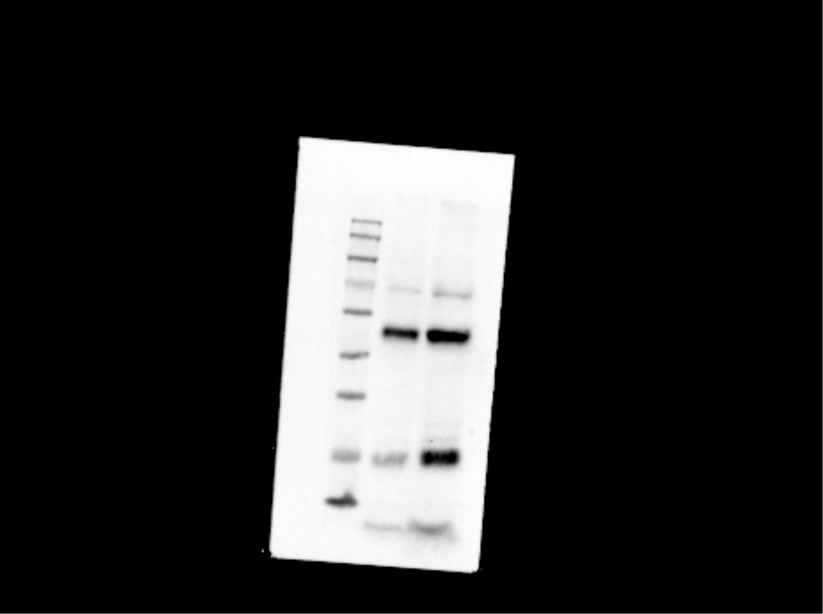


Full and uncropped western blots for Figure S12C-3


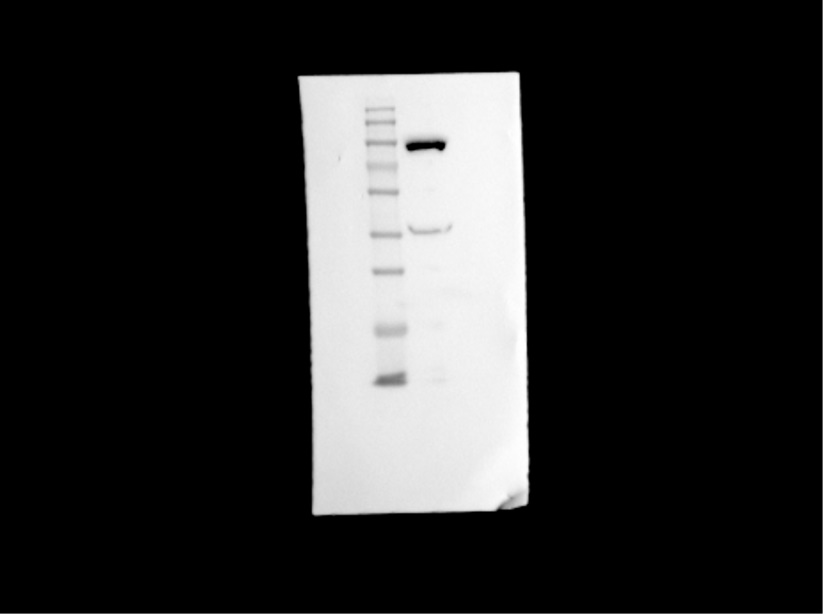


Full and uncropped western blots for Figure S12C-4

Full and uncropped western blots for Figure S13A-1 (1)

Full and uncropped western blots for Figure S13A-1 (2)

Full and uncropped western blots for Figure S13A-2 (1)

Full and uncropped western blots for Figure S13A-2 (2)

Full and uncropped western blots for Figure S13A-3 (1)

Full and uncropped western blots for Figure S13A-3 (2)

Full and uncropped western blots for Figure S13A-4-1

Full and uncropped western blots for Figure S13A-4-2

Full and uncropped western blots for Figure S13A-5

Full and uncropped western blots for Figure S13A-6 (1)

Full and uncropped western blots for Figure S13A-6 (2)

Full and uncropped western blots for Figure S13I-1

Full and uncropped western blots for Figure S14D-1 (1)

Full and uncropped western blots for Figure S14D-2

Full and uncropped western blots for Figure S14D-3 (2)

Full and uncropped western blots for Figure S14D-4 (1)

Full and uncropped western blots for Figure S14D-5

Full and uncropped western blots for Figure S14D-6

Full and uncropped western blots for Figure S14D-7

Full and uncropped western blots for Figure S14D-8

Full and uncropped western blots for Figure S14D-9

Full and uncropped western blots for Figure S14D-10

Full and uncropped western blots for Figure S14D-11
